# Effects of Dopants and Processing Parameters on the Properties of ZnO-V_2_O_5_-Based Varistors Prepared by Powder Metallurgy: A Review

**DOI:** 10.3390/ma16103725

**Published:** 2023-05-14

**Authors:** Magdalena Valentina Lungu

**Affiliations:** Metallic, Composite and Polymeric Materials Department, National Institute for Research and Development in Electrical Engineering ICPE-CA, 030138 Bucharest, Romania; magdalena.lungu@icpe-ca.ro; Tel.: +40-723-686-334

**Keywords:** metal oxides, ceramic materials, ZnO-V_2_O_5_-based varistors, powder metallurgy route, functional properties

## Abstract

This article reviews the progress in developing ZnO-V_2_O_5_-based metal oxide varistors (MOVs) using powder metallurgy (PM) techniques. The aim is to create new, advanced ceramic materials for MOVs with comparable or superior functional properties to ZnO-Bi_2_O_3_ varistors using fewer dopants. The survey emphasizes the importance of a homogeneous microstructure and desirable varistor properties, such as high nonlinearity (α), low leakage current density (J_L_), high energy absorption capability, reduced power loss, and stability for reliable MOVs. This study investigates the effect of V_2_O_5_ and MO additives on the microstructure, electrical and dielectric properties, and aging behavior of ZnO-based varistors. The findings show that MOVs with 0.25–2 mol.% V_2_O_5_ and MO additives sintered in air over 800 °C contain a primary phase of ZnO with a hexagonal wurtzite structure and several secondary phases that impact the MOV performance. The MO additives, such as Bi_2_O_3_, In_2_O_3_, Sb_2_O_3_, transition element oxides, and rare earth oxides, act as ZnO grain growth inhibitors and enhance the density, microstructure homogeneity, and nonlinearity. Refinement of the microstructure of MOVs and consolidation under appropriate PM conditions improve their electrical properties (J_L_ ≤ 0.2 mA/cm^2^, α of 22–153) and stability. The review recommends further developing and investigating large-sized MOVs from the ZnO-V_2_O_5_ systems using these techniques.

## 1. Introduction

Voltage surge protection devices (SPDs) or surge arresters rely on metal oxide varistors (MOVs) to safeguard electrical equipment in consumer electronics and industrial electric power systems against the destructive temporary overvoltages (TOVs) resulting from transient switching surges or lightning strikes [1,2]. The primary function of voltage-sensitive MOVs in SPDs is to prevent the damage caused by high-energy transients by clamping or eliminating them when a surge occurs. These MOVs are mounted in parallel with the components that they are designed to protect [3].

MOVs are sintered ceramic materials typically manufactured in a disc shape that resembles a right circular cylinder, although rectangular or hexagonal prism shapes with flat surfaces are reported less frequently [4]. MOVs are complex, multicomponent systems that consist of a semiconducting oxide [5] such as zinc (II) oxide (ZnO) [6,7,8,9], tin (IV) oxide (SnO_2_) [10,11], or titanium (IV) dioxide (TiO_2_) [12,13] as the main component (>90 mol.%), and multiple MO additives as minor components.

The role of MO additives is to enhance the performance of MOVs through a synergistic effect [14]. For example, Bi_2_O_3_ [15,16], V_2_O_5_ [17,18], and Pr_6_O_11_ [19] are varistor-forming oxides (VFOs) or nonlinearity inducers that can form solid solutions with other MOs. Varistor-enhancing dopants (VEDs) act as grain growth inhibitors and densification enhancers and are selected from rare earth oxides (REOs) such as Er_2_O_3_ [20,21,22], Gd_2_O_3_ [23], Dy_2_O_3_ [24], Sm_2_O_3_ [25], Y_2_O_3_ [25,26,27], La_2_O_3_, and CeO_2_ [28], among others.

Transition element oxides (TEOs) such as Co_2_O_3_ [29], Co_3_O_4_ [30], Cr_2_O_3_ [8,31], NiO [32,33], CuO [34], MnO [35], MnO_2_ [36,37], Mn_3_O_4_ [27,38], Nb_2_O_5_ [28,39], Fe_2_O_3_ [40], ZrO_2_ [41], WO_3_ [42], and other MOs (In_2_O_3_ [43,44], Al_2_O_3_ [43], Sb_2_O_3_ [6,30], B_2_O_3_ [45], SiO_2_ [46], etc.), as well as TiO_2_ [47] and SnO_2_ [48], are used as VEDs. However, it is a challenge to determine the distinct contribution of each MO dopant in multicomponent MOVs that exhibit complex structures [14]. Moreover, the properties of MOVs depend on the type and content of dopants, as well as the thermal history of the MOVs.

Small-sized MOV discs (cylindrical blocks) with a diameter × height of Ø8–20 mm × 1–4 mm are typically used in voltage SPDs for high-voltage applications in consumer electronics. MOV discs of larger sizes (diameter × height of Ø20–120 mm × 20–50 mm) can be used to equip surge arresters for electric power systems with switching voltages of a few kV [3]. Reference [49] illustrates some MOVs of different shapes and sizes, an SPD, and a silicon-housed gapless MOSA equipped with MOVs.

### 1.1. Specific Features of ZnO-Based Varistors

MOVs display distinctive characteristics, such as significant nonlinearity in the current–voltage (I–V) curves and the current density–electric field (J–E) curves, respectively.

The relationship between the current (I, in mA) passing through the MOV and the voltage (V, in volts) applied across the MOV is defined by Equation (1) [6]:

(1) $$I={\left(\frac{V}{C}\right)}^{\alpha }=kV^{\alpha }$$

where C is a constant related to the resistance of the MOV (C is the ratio between voltage and unit length (V/mm) when a current density of 1 mA/cm^2^ flows through the MOV), k is a constant equal to (1/C)^α^, and α is the nonlinear coefficient (α > 1 is necessary to diverge from the linearity of I–V or J–E, in agreement with Ohm’s law [50]).

The significant regions of the I–V curves of MOVs are as follows: (i) the prebreakdown or leakage region (I < 10 μA), (ii) the nonlinear region or normal varistor operation (10 μA < I < 1 kA), and (iii) the upturn region (I > 1 kA) [51,52]. At low currents, MOVs can reach very high resistance (≤10 GΩ) in the leakage region, which varies with temperature and frequency. At high currents, low resistance equal to the bulk resistance of ZnO grains (1–10 Ω) can occur in the upturn region [51,53]. In the nonlinear region (conduction mode), MOVs can achieve a high energy absorption capability.

The AC total leakage current (I_L_) in the prebreakdown region (I < 10 μA) is the sum of the resistive current (I_R_) and capacitive current (I_C_). The I_R_ is the ratio between the steady state voltage (V_SS_) and grain boundary resistance (R_gb_), producing Joule heating in the MOVs. The main parameters affecting the I_L_ are (i) the formulation of the ceramic materials, (ii) the applied voltage, and (iii) the duration of the applied voltage [54].

Normal varistor operation begins at a threshold or breakdown voltage (V_B_) at a current of 1 mA, denoted as V_1mA_, when α can reach a maximum value [52,53,55]. An α value greater than 30 suggests good contact between ZnO grains [53], while an α value less than 10 indicates poor boundary junctions between ZnO grains, resulting in unsatisfactory electrical (E–J) characteristics of MOVs [53,56]. Typical α values of ZnO-based varistors range from 20 to 70 [57].

MOVs are voltage-dependent resistors, meaning that their electrical resistance varies greatly depending on the applied voltages [54]. They are nonohmic resistors because their electrical resistance decreases as the applied voltage increases [6].

The microstructure of ZnO-based varistors must be dense, polycrystalline, homogeneous, and conductive, consisting of a matrix of ZnO grains (the primary phase) interconnected by resistive grain boundaries (GBs) comprising multiple MO additives. Each intergranular boundary depends on the grain size, creating a distinct voltage barrier that, when conducting, forms a low ohmic path capable of absorbing surge energy. Accordingly, these GBs created during sintering provide the specific p–n junction semiconductor features of MOVs, representing the fundamental conduction mechanism of MOVs [14,51,58].

Each active ZnO-ZnO GB can yield a charge barrier (V_B_) of about 3–3.5 V, acting as a switching diode [52,53,55,59]. The total breakdown voltage of an MOV depends on the number of active ZnO-ZnO GBs between two metal electrodes (e.g., Ag, Al, Pt, Pd, or their alloys, etc.) [60,61,62,63] formed on the top and bottom plane surfaces of a ceramic MOV disc.

In practical applications of ZnO-based varistors, the individual electrical barriers are typically assumed identical for theoretical purposes, even though ZnO grains can differ in size, shape, and orientation [64]. This is why researchers consider the mean ZnO grain size when determining the characteristics of MOVs.

Table 1 summarizes the critical parameters and functions of MOV devices, along with several typical values of the electrical parameters.

An increase in the leakage current (I_L_) or leakage current density (J_L_), along with a decrease in the nonlinear coefficient (α) and breakdown field (E_B_) near 1 mA/cm^2^, usually indicates the degradation phenomenon of ZnO-based varistors [67]. Many studies reported that α and I_L_ or J_L_ are more appropriate indicators for the degradation of MOVs than E_B_ [67,68]. Hence, obtaining high α and E_B_ and low I_L_ or J_L_ can enhance the protection of devices and ensure a longer lifetime of MOVs [6,31,54].

Other notable characteristics of MOVs are high energy absorption capability, reduced power loss, and good stability against aging (degradation) processes [69,70,71,72]. For instance, ZnO-based systems for use in 1000 kV surge arresters in ultra-HV grids have to exhibit a high voltage gradient (E_B_ > 4 kV/cm) and energy absorption capability > 300 J/cm^3^ to improve the performance of MOSAs with reduced height [69,73].

The technical properties of MOVs have a direct impact on the functional performance of the SPDs and MOSAs equipped with MOVs. The I–V characteristics, energy absorption capability, power losses, residual voltages, continuous voltage, and flashover behavior are among the main properties considerably determined by the MOV discs [66,74,75].

The diameter and height of MOV discs significantly affect the current and voltage rating [76]. The admitted leakage current (I_L_) of MOVs is a few hundred µA at the operating voltage. Common values for the DC leakage current (I_Ld_) are lower than 100 μA for small-sized MOV discs and 200 μA for large-sized MOV discs used in electric power and telecommunication SPDs. The MOV size and voltage rating influence the capacitance (C) and clamping voltages (V_c_) of MOVs, since typical C values can be from 3 pF to 0.03 μF, and V_c_ can be from 17 V to 4 kV. In addition, the increase in the diameter (surface area) of the MOV discs results in a higher energy absorption capability (E), and vice versa. A high value of the surface-to-volume ratio of the MOV discs leads to higher E values [66].

Reliable MOVs have to exhibit high nonlinearity (α) and energy absorption capability, a low leakage current (I_L_) or leakage current density (J_L_), and long-term stability [77]. Low I_L_ and J_L_ values can avoid thermal runaway, while satisfactory stability and aging behavior are preserved, besides reduced electric power consumption [8]. However, we do not expect MOVs with the highest nonlinearity to have the highest energy absorption capability [77]. Furthermore, high E_B_ can improve surge protection and ensure the reduction of the device size in HV power systems. Nonetheless, the E_B_ increase can decrease the nonlinearity (α) and increase I_L_ and J_L_. Therefore, state-of-the-art studies of ZnO-based varistors commonly report values of α < 60, J_L_ > 1 μA/cm^2^, and E_B_ of 2–5 kV/cm [8].

### 1.2. Brief History of ZnO-Based Varistors

The varistor effect of MOV ceramics (mol.%) composed of 97–99.5% ZnO, 0.5% Bi_2_O_3_, and MO additives (Sb_2_O_3_, CoO, MnO_2_, and Cr_2_O_3_) was discovered over five decades ago [6]. MOV discs were obtained via the conventional PM route, which involved pressing MOV powders at around 33 MPa in green compacts (Ø17.5 mm × 2 mm) and sintering in air at 950–1450 °C. The ZnO-0.5% Bi_2_O_3_-1% Sb_2_O_3_-0.5% CoO-0.5% MnO_2_-0.5% Cr_2_O_3_ discs sintered at 1350 °C exhibited the best nonlinearity (α = 50, E_B_ = 1.35 kV/cm) due to the formation of segregation layers at the GBs. However, the ZnO-0.5% Sb_2_O_3_ or Bi_2_O_3_ systems, when sintered at 1150 °C, yielded low nonlinear properties (α = 3.1–4, E_B_ = 0.1–0.65 kV/cm) due to the absence of segregation layers. When sintering was done at 1450 °C, Bi_2_O_3_ and Sb_2_O_3_ evaporated, and CoO, MnO_2_, and Cr_2_O_3_ dissolved, preventing the formation of segregation layers and the acquisition of nonlinear properties. All ZnO-based systems doped with 2–4 MOs (Bi_2_O_3_ + CoO/MnO_2_, Bi_2_O_3_ + CoO + MnO_2_, or Bi_2_O_3_ + CoO + MnO_2_ + Cr_2_O_3_) at a concentration of 0.5 mol.% for each MO, and sintered at 1250–1350 °C, resulted in low nonlinear properties (α = 13–22, E_B_ ≤ 0.5 kV/cm).

ZNR^TM^, produced by Matsushita Electric Industrial Co., Osaka, Japan (now Panasonic), in 1968, and GE-MOV™, produced by General Electric Co., Pittsfield, MA, USA in 1972, were among the first commercial MOVs based on ZnO, Bi_2_O_3_, and several MO dopants for use in the electronics industry [14,34,78]. The developed disc-shaped MOVs typically had a diameter of Ø7–20 mm and a thickness of 1–3 mm and exhibited high nonlinearity (α of 25–50), as well as increased energy absorbing capability (<200 J at a surge current of 6 kA).

The pioneering ZnO blocks used in gapless MOSAs were produced and commercialized by Meidensha Electric Manufacturing Co. Ltd., Numazu, Japan, and General Electric Co., Pittsfield, MA, USA in the mid-1970s [78,79]. Since then, these and other companies worldwide have developed and commercialized MOVs with different sizes and formulations and improved varistor characteristics for a broad range of applications. Moreover, technological advancements in MOVs are still ongoing today.

Most commercial ZnO-based varistors are composed of ZnO-Bi_2_O_3_ systems with up to 8–12 MO dopants and typically yield ZnO grain sizes (d_ZnO_) of 10–100 μm and a depletion length of the doped GBs of 50–100 nm, a breakdown field (E_B_) of 2–3 kV/cm, and a nonlinear coefficient of about 40–50 [52,56,59,80,81,82]. MOVs with coarse-grained microstructures (mean d_ZnO_ > 30 µm) and lower E_B_ values are used for low-voltage (LV) applications, while MOVs with fine-grained microstructures (mean d_ZnO_ < 10 µm) are required for high-voltage (HV) and medium-voltage (MV) applications [59,83].

The interest in developing ZnO-V_2_O_5_ systems as alternative solutions to ZnO-Bi_2_O_3_ and ZnO-Pr_6_O_11_ systems began approximately 30 years ago. In comparison, the first studies on ZnO-Bi_2_O_3_-based systems started around 50 years ago [6].

**Figure 1:**
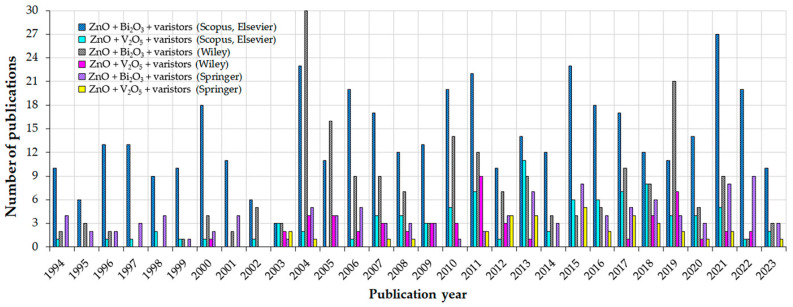
The number of publications on MOVs from ZnO-Bi_2_O_3_- and ZnO-V_2_O_5_-based systems published from January 1994 to April 2023 in Scopus, Elsevier, Wiley, and Springer databases. The data plotted in the figure were collected using keywords ZnO + Bi_2_O_3_ or V_2_O_5_ + varistors.

Many articles published in the last 30 years (Figure 1) confirm the increasing significance of MOVs from ZnO-Bi_2_O_3_- and ZnO-V_2_O_5_-based systems.

Different patent and trademark offices from the USA, Europe, and Japan, among others (e.g., USPTO, EPO, JPO, etc.), and intellectual property organizations (e.g., WIPO, CNIPA from China, etc.) protect hundreds of international patents on MOVs.

This article focuses on ZnO-V_2_O_5_-based varistors, which have garnered considerable attention in both academia and industry for their potential to achieve advanced ceramic materials with properties comparable to, or even superior to, those of ZnO-Bi_2_O_3_-based varistors. Interested researchers may refer to previous reviews on ZnO-Bi_2_O_3_-based varistors cited in references [54,56,65,84]. However, specific reviews on ZnO-V_2_O_5_-based varistors are scarce in the current literature. Therefore, this article summarizes the main findings of numerous studies on ZnO-0.25–4 mol.% V_2_O_5_ systems, which were developed using powder metallurgy (PM) routes and feature many different compositions. This review comprehensively describes how different dopants and processing parameters affect the microstructure and characteristics of ZnO-V_2_O_5_ systems. It also addresses the limitations of current research and suggests future directions for industrial applications. By exploring the complexity and challenges in this fascinating field, this review provides up-to-date and informative insights for further research.

## 2. Overview of the Development of ZnO-Based Varistors

### 2.1. Preparation Methods of Elemental MOs and Composite Powders for MOVs

**Figure 2:**
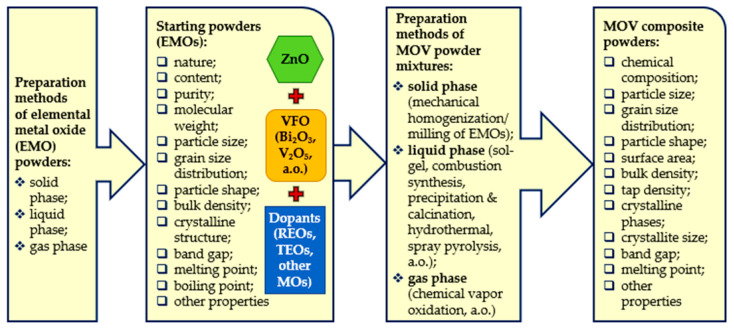
Schematic illustration of the dependence between the preparation methods and properties of elemental metal oxide (EMO) powders and MOV powder mixtures.

Elemental metal oxide (EMO) powders and MOV composite powders composed of EMOs (ZnO, VFO, and MO additives) are typically obtained by (i) solid-phase, (ii) liquid-phase, and (iii) gas-phase methods [54]. Each method has advantages and disadvantages (Table 2) that influence the characteristics of the developed powders (Figure 2).

The solid-state method (mechanical homogenization/milling of powders) is a classical method applied on a laboratory scale and in the industry [54,70]. However, producing MOV composite powders with high purity, uniformity, and a narrow grain size distribution is difficult.

The liquid-phase methods include sol–gel methods [85,86], the precipitation or coprecipitation and subsequent calcination of powders [70], hydrothermal methods [87,88], atomization and spray drying, and flame spray pyrolysis [89]. These methods can yield reasonable control of the content and shape of MOV powders. However, a challenge is to achieve mass production at low costs. Similar issues can occur in the gas-phase methods, including chemical vapor oxidation methods [54,90]. Combustion synthesis is an alternative technique with a simple and energy-efficient setup for producing doped ZnO ceramic powders with a controlled composition for the manufacturing of sintered MOVs [54,91].

### 2.2. Preparation Methods and Major Assessment Criteria of ZnO-Based MOVs

MOV ceramic materials can be prepared using various powder metallurgy (PM) techniques that involve pressing MOV powders and consolidating them by sintering.

**Figure 3:**
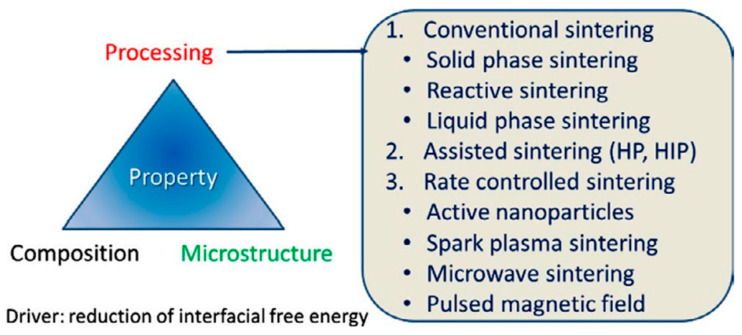
Schematic illustration of the dependence between the microstructure, composition, and other technical characteristics of MOVs and various PM processing techniques. Reprinted from reference [93], published under an open access Creative Commons Attribution (CC BY 4.0) license (https://creativecommons.org/licenses/by/4.0/ (accessed on 17 December 2022)). Copyright © 2019 The Authors.

The sintering process can be classified as conventional or non-conventional, as shown in Figure 3 [93]:

➢ conventional sintering (CS), which includes solid-phase sintering (CS-SPS), reactive sintering (CS-RS), and liquid-phase sintering (CS-LPS);
➢ assisted sintering involving hot pressing (HP) or hot isostatic pressing (HIP);
➢ rate-controlled sintering (RCS) by using (i) active nanoparticles (NPs); (ii) spark plasma sintering (SPS), which is an electric-field-assisted process; (iii) microwave sintering (MWS), or (iv) pulsed magnetic field (PMF) processes.

Each of these processes has its own advantages and disadvantages, and the choice of process depends on the available infrastructure and the production capacity of the manufacturers of MOVs.

Conventional PM techniques are widely used in both laboratory and industrial settings due to their high productivity in manufacturing a large number of MOVs. 

Non-conventional PM techniques, such as SPS [94,95,96,97] and MWS [98,99,100], are faster but have lower productivity and are mostly used on a laboratory scale. On the other hand, HP and HIP processes [97] consume a large amount of protective gas (argon or nitrogen) used as the pressurizing medium and have a longer overall duration, resulting in a limited number of MOVs.

The microstructure and technical characteristics of ZnO-based varistors heavily rely on several factors, such as the nature, content, grain size, and grain size distribution of ZnO, which is the principal component [101,102]. Additionally, the selected VFO and dopants [7,103,104,105,106,107] also play a crucial role. Furthermore, the techniques used for preparing MOV powder mixtures [108,109], as well as the consolidation (pressing of MOV powders) and densification techniques (sintering of MOV compacts) (Figure 3), including post-annealing and post-processing techniques of MOVs [13,76,77,94,110,111], have a significant impact on the properties of MOVs.

The primary assessment criteria of MOVs should consider several factors, including technical assessment criteria related to the microstructure and characteristics of MOVs in accordance with relevant standards. Additionally, it is important to consider the impact on the environment and human health caused by the raw materials and waste generated during the production, processing, utilization, and recycling of MOVs. Compliance with relevant regulations, such as the Restriction of Hazardous Substances (RoHS) in Electrical and Electronic Equipment (EEE) directives in the European Union (EU) and Waste EEE regulations, must also be considered. Lastly, economic assessment criteria should also be taken into account, including expenses for raw materials, consumables, auxiliary materials, labor, and energy consumption.

Hassazadeh et al. [112] proposed effective solutions to eliminate and reduce solid, liquid, and gaseous industrial wastes produced during the manufacture of MOV discs used in lighting surge arresters, resulting in environmental enhancements and improved electrical characteristics of MOVs.

### 2.3. Liquid-Phase Sintering (LPS) of ZnO-Bi_2_O_3_- and ZnO-V_2_O_5_-Based Systems

ZnO-Bi_2_O_3_-based systems are typically sintered at high temperatures, ranging from 1100 °C to 1400 °C, for up to 24 h. This is necessary to achieve a homogeneous microstructure and adequate varistor characteristics. Sintering below 1100 °C can lead to MOVs with an inhomogeneous microstructure, reduced and irregular grain growth of ZnO, a high breakdown field, low nonlinearity, and unsatisfactory repeatability of electrical characteristics, as reported in [113]. The non-ohmic behavior of MOVs is conferred by the electrostatic potential barriers, known as double Schottky barriers (DSBs), which are created during cooling at the GBs of ZnO [114,115,116,117].

The growth rate of ZnO grains during the sintering of ZnO-Bi_2_O_3_-based systems is highly dependent on the Bi_2_O_3_ content. Adding 0.05 mol.% Bi_2_O_3_ can lead to the abnormal growth of ZnO grains in direct contact with Bi_2_O_3_ due to incomplete wetting [59]. However, an amount of 0.05–1 mol.% Bi_2_O_3_ improves ZnO grain growth and enhances the densification of ZnO-Bi_2_O_3_-based systems by liquid-phase sintering (LPS). This is because Bi_2_O_3_ melts at 825 °C, and the eutectic temperature of the binary ZnO-Bi_2_O_3_ systems is around 740 °C [118,119]. When the Bi_2_O_3_ content exceeds 1 mol.%, diffusion paths are generated through the liquid phase, reducing ZnO grain growth [59]. Therefore, ZnO-Bi_2_O_3_-based systems have a specific content of 0.5–1 mol.% Bi_2_O_3_ in MOV formulations [6,59].

The liquid phase localized at the GBs improves diffusion development, and ZnO grain growth increases due to a solution-precipitation process governed by a phase-boundary reaction mechanism. However, a continuous Bi-rich skeleton adjoining ZnO grains can generate a supplementary current, leading to a higher leakage current [120]. Additionally, an increase in the mass loss of ZnO-Bi_2_O_3_-based varistors occurs with the formation of the eutectic liquid, suggesting the vaporization of Bi_2_O_3_, which increases with the sintering temperature and dwell time [119].

Bi_2_O_3_ has high volatility and considerable reactivity during LPS above 1000 °C, affecting the microstructure and performance of the ZnO-Bi_2_O_3_ systems [121,122]. When the volatilization of Bi_2_O_3_ is not prevented (e.g., by sealed sintering with Al_2_O_3_-covered crucibles), unsuitable GB layers can result [121]. The liquid phase can enhance GB mobility, but the formed intragrain pores can decrease the sintered density of MOVs [118]. Lower-density MOVs (RD < 95% of TD) can yield defects such as spherical holes, indicating inadequately joined grains, leading to a decrease in the mechanical strength of MOVs [111].

ZnO-Bi_2_O_3_ multilayer varistors are typically sintered at high temperatures (above 1000 °C) and co-fired with inner electrodes such as Ni [123], Pd, or Pt alloys [124]. The use of ZnO-V_2_O_5_ systems as an alternative to ZnO-Bi_2_O_3_ systems offers the advantage of sintering at lower temperatures (800−900 °C) to create DSBs, along with co-firing with an Ag inner electrode on each circular surface because the melting temperature of Ag is 960 °C [60,61,125].

An amount of V_2_O_5_ ≥ 0.5 mol.% improves the densification of ZnO-V_2_O_5_-based varistors by LPS, as V_2_O_5_ melts at 681 °C, and the eutectic temperature of the binary ZnO-V_2_O_5_ system is around 600 °C [126,127]. However, ZnO-V_2_O_5_-based systems with V_2_O_5_ content over 2 mol.% usually exhibit unsatisfactory varistor properties due to excessive secondary phases formed at the GBs between ZnO grains. On the other hand, V_2_O_5_ content of less than 0.1 mol.% does not promote the LPS mechanism at 825−900 °C, hindering the densification of MOVs [128].

The prominent roles of MO additives in the varistor characteristics consist of influencing the ZnO grain growth process during the sintering stage and the dewetting features of the liquid phase during the cooling stage. Furthermore, introducing dopants into the host lattice of the ZnO crystal structure changes the electronic defect states that govern the overall MOV features [14].

## 3. Considerations of the Crystalline Structure and Physics of ZnO-Based Varistors

### 3.1. Crystalline Structure of ZnO

**Figure 4:**
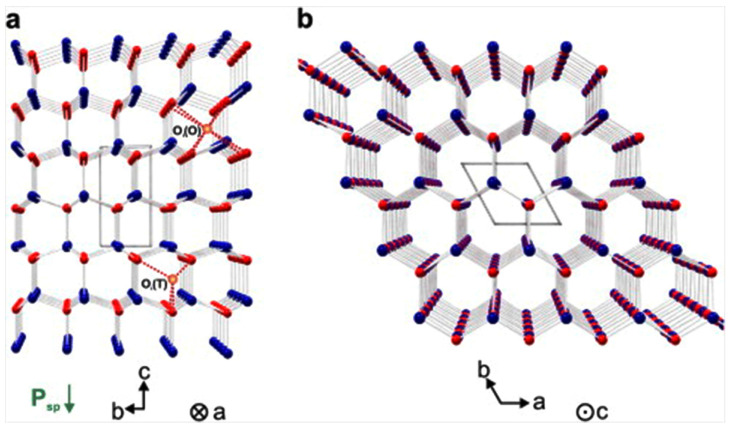
Schematic view of the wurtzite ZnO crystal structure along (**a**) the a-axis and (**b**) antiparallel to the c-axis (the red and blue dots represent the Zn and O lattice sites, respectively). Reprinted from reference [129] with permission from Elsevier Ltd. © 2009.

A ZnO semiconductor has a wurtzite crystal structure with a hexagonal P6_3_mc space group, and the lattice parameters a = b = 3.2490 Å and c = 5.2042 Å [129]. ZnO is greatly ionic, with a Zn^2+^ cation bonded to four O^2−^ anions in a tetragonal configuration. The crystal structure of wurtzite ZnO contains close-packed (cp) planes of Zn and O atoms, positioned alternatively in a hexagonal (hcp) stacking along the polar c-axis (Figure 4) [129].

The stacking of Zn and O layers along the c-axis generates a positive and a negative net charge on the (0001) and (0001¯) surface planes, respectively.

The orientation of the wurtzite ZnO crystal influences various material characteristics, such as the grain growth kinetics, incorporation of structural defects and metal impurities, piezoelectric behavior, etc. [129,130].

### 3.2. Polymorphisms of Bi_2_O_3_ and V_2_O_5_

The main polymorphisms of Bi_2_O_3_ are the stable phases α and δ, along with the metastable phases β and γ. The phase transitions in Bi_2_O_3_ depend on various factors, such as the heating temperature (720–900 °C), cooling rate (1–16 °C/min), heating time (2–4 h), and atmosphere (air, oxygen, or argon), leading to different oxygen content in Bi_2_O_3_ [131]. The α to δ transition occurs at 730 °C during the heating of Bi_2_O_3_ in air. The formation of δ- and β-Bi_2_O_3_ phases occurs at 745–840 °C and 633–637 °C, respectively, upon melt cooling with about 2.5–10 °C/min. The formation of γ- and α-Bi_2_O_3_ phases occurs at temperatures of 635–641 °C and 475–493 °C, respectively, upon melt cooling with 1–2 °C/min. At a heating temperature of 875–900 °C, the formation of the δ-Bi_2_O_3_ phase occurs at temperatures of 612–627 °C, which changes into the α-Bi_2_O_3_ phase during cooling. This investigation estimates the oxygen deficit (x) in the Bi_2_O_3–x_ system as x = 0–0.026 in the α phase, x = 0.023 in the β phase, x = 0.022 in the γ phase, and x = 0.022–0.33 in the δ phase [131].

Mielcarek et al. [132] reported on all the polymorphic phases of Bi_2_O_3_ in ZnO-Bi_2_O_3_-based systems. The formation of the α-Bi_2_O_3_ phase at GBs or triple grain junctions (TGJs) occurs upon cooling MOVs [84]. The α-Bi_2_O_3_ to γ-Bi_2_O_3_ phase transition occurs during the annealing of sintered MOVs, while the α-Bi_2_O_3_ to δ-Bi_2_O_3_ transition at the GBs or TGJs is produced by the presence of metal dopants (e.g., Al, Si, etc.) in MOVs, or by quenching.

The type and content of the polymorphs of Bi_2_O_3_ in MOVs depend highly on the sintering temperature, dwell time, heating/cooling rate, and the composition of ZnO-Bi_2_O_3_-based varistors [120,133]. The δ- and β-Bi_2_O_3_ phases are good ionic conductors, promoting the oxygen migration from the GBs, but the γ- and α-Bi_2_O_3_ phases are low ionic conductors. The α-Bi_2_O_3_ phase yields p-type electronic conductivity at room temperature (RT), while the δ-Bi_2_O_3_ phase has ionic conductivity with oxide ions as the majority charged carriers [121]. The polymorphs of Bi_2_O_3_ influence the existence and content of oxygen at the interface across ZnO grains, affecting the behavior of MOVs in service [132].

**Figure 5:**
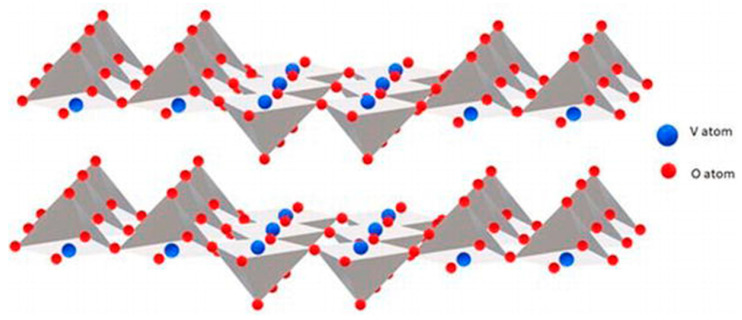
Schematic view of the crystalline structure (two layers) of α-V_2_O_5_ (the blue and red spheres represent V and O atoms, respectively). Reprinted from reference [135], published under an open access Creative Commons Attribution (CC BY 3.0) license (http://creativecommons.org/licenses/by/3.0 (accessed on 20 February 2023)). Copyright © 2021 The Authors.

V_2_O_5_ exhibits multiple polymorphic phases (α, β, γ, δ, β′ or ζ, γ’, δ′, ρ′, and ε′), from which the α phase is the most thermodynamically stable and commercialized phase [134]. Detailed information on the polymorphs of V_2_O_5_ is reported in references [134,135,136]. The metastable states of V_2_O_5_ result from the preparation conditions (temperature and pressure), along with a few limitations (surface confinement, tensile/compressive stress, electrochemical intercalation/deintercalation (reversible), etc.). Figure 5 illustrates the crystal structure of α, β, and γ polymorphs of V_2_O_5_ [135].

The polymorphs of V_2_O_5_ exhibit crystal structures with distinct atomic arrangements (V–O connectivity) and different bonding motifs (strength). Thus, the electronic structure and the energy band gap are variable for each polymorph of V_2_O_5_ and can be adjusted in the range of semiconductor materials [135] for the intended application. However, the reviewed literature reports on ZnO-V_2_O_5_-based systems did not address this issue.

### 3.3. Crystallographic Defects and Doping of ZnO

**Figure 6:**
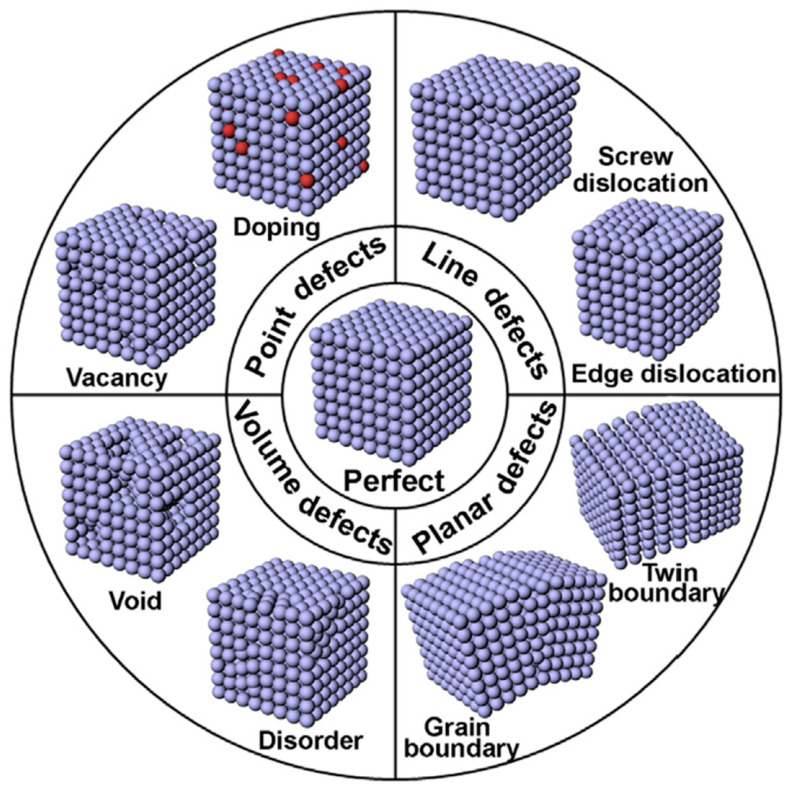
Schematic view of the main types of defects with various atomic structures in MO semiconductor photocatalysts such as ZnO, TiO_2_, SnO_2_, In_2_O_3_, etc. Reprinted from reference [137] with permission from Elsevier Ltd. © 2018.

Crystallographic defects (Figure 6) are essential characteristics in designing MO semiconductors, such as ZnO, TiO_2_, SnO_2_, In_2_O_3_, and so on [137,138]. The field of semiconductor physics, including defect physics, has been extensively studied and presented in many state-of-the-art publications [102,130,137,139,140,141,142].

In multicomponent ZnO-based varistors, bulk, surface, and interfacial defects can occur between ZnO and multiple MO additives. The interfacial defects are similar to the surface defects, and can be composed of point defects (vacancies and foreign metal doping), volume defects (voids and disorders), or end of line defects (screw or edge dislocations), as well as planar defects (GBs and twin boundaries) [137]. Grain boundaries are the most commonly reported planar defects in ZnO, along with point defects.

The primary native defects found in ZnO are Zn vacancies (V_ZnO_), O vacancies (V_O_), Zn interstitials (Zn_i_), O interstitials (O_i_), Zn antisites (Zn_O_), and O antisites (O_Zn_). In Zn antisites, Zn can substitute for O, while, in O antisites, O can substitute for Zn. Kröger–Vink notation is the standard notation for defects in ionic crystals, and an example of defect formation in ZnO-V_2_O_5_-based systems is given in reference [143].

A ZnO semiconductor usually has an n-type structure in which negatively charged electrons (e^−^) are the majority charge carriers, while positively charged holes (h^+^) are the minority charge carriers. The n-type conductivity of semiconductors is caused by structural point defects (vacancies and interstitials) and extended defects (surface conduction, dislocations, stacking faults, and grain boundaries) [130].

The origin of the n-type conductivity of ZnO is attributed to the presence of V_O_ and Zn_i_ defects in the ZnO lattice under O-rich and Zn-rich conditions. Both experimental works, such as IR spectroscopy, and theoretical studies, such as first-principles calculations, have disclosed that O vacancies behave as deep donors, while Zn interstitials are shallow donors providing electrons but are unstable at RT due to their high mobility [140].

The doping type of a semiconductor oxide is defined by the number of valence electrons of the dopant (metal impurity). Doping ZnO with a trivalent element (B, Al, Ga, In) produces p-type doping, while adding a pentavalent element (Bi, Sb, As, P) leads to extrinsic n-type doping. Moreover, doped ZnO is electrically neutral [141].

Theoretical calculations have shown that the acceptor defects (O_i_, V_Zn_, and O_Zn_) have higher formation enthalpies than those for the donor defects (Zn_i_, V_O_, and Zn_O_) [130].

The dominant native defects in p-type ZnO, where positive charge holes are the majority charge carriers, are the donor defects with low formation enthalpies that have a higher probability of generation at thermal equilibrium than those of the acceptor defects.

The factors related to the dopants, such as their formation and ionization energy, and the solubility of dopants, define the effects of MO doping on ZnO semiconductors [142].

It is possible to position foreign metal dopants (ions or atoms) in the ZnO host lattice either by substituting the existent ions or atoms or by placing them at the interstitial sites. In the first case, the radii of the metal dopants have to be comparable with those of ZnO, whereas, in the second case, the radii of interstitial metal dopants are much lower [137].

In multicomponent ZnO-based varistors, each introduced dopant in the host lattice of the ZnO crystal structure can act as a donor (e.g., Y ions from Y_2_O_3_, Mn ions from Mn_3_O_4_) [26,38,144] or an acceptor (e.g., Mn ions from MnO_2_) [144], or both (e.g., Mn ions with various valence states) [144], depending on the ionic radius and relative valency of the guest (dopant) metal ions and host (ZnO) ions [14].

Zn^2+^ with the coordination number (CN) of VI has an ionic radius (IR) of 0.06 nm and a stable electronic configuration (3d10) [145]. Metal cations with CN of VI used as common dopants of ZnO have IR of 0.103 nm (Bi^3+^), 0.1032 nm (La^3+^), 0.0983 nm (Nd^3+^), 0.0958 nm (Sm^3+^), 0.0938 nm (Gd^3+^), 0.0912 nm (Dy^3+^), 0.09 nm (Y^3+^), 0.089 nm (Er^3+^), 0.076 nm (Sb^3+^), 0.069 nm (Ni^2+^), 0.067 nm (Mn^2+^), 0.064 nm (Nb^5+^, Ta^5+^), 0.062 nm (In^3+^), 0.0615 nm (Cr_2_^3+^), 0.058 nm (Co^2+^, Mn^3+^), 0.054 nm (V^5+^), 0.053 nm (Mn^4+^), 0.047 nm (Ga^3+^), etc. [145].

Adding insoluble MOs such as Bi_2_O_3_, In_2_O_3_, Cr_2_O_3_, NiO, Nb_2_O_5_, and Ta_2_O_5_, among others, and REOs (e.g., Dy_2_O_3_, La_2_O_3_, Gd_2_O_3_, Nd_2_O_3_, Er_2_O_3_, Y_2_O_3_, Sm_2_O_3_, etc.) with a larger ionic radius than that of ZnO can generate varistor behavior in ZnO-based systems. This addition causes the metal oxides to segregate at the ZnO GB regions and create donor states to enhance electron conduction among n-type ZnO grains [146,147].

MOs with lower ionic radii than that of ZnO, such as CoO, Mn_3_O_4_, MnO_2_, V_2_O_5_, Ga_2_O_3_, and others, are also added to ZnO-based systems to improve their electrical properties. This is achieved by forming more donor states, which enhances electron conduction and increases the nonlinearity of the varistors. The reduction of the leakage current occurs by decreasing the number of defects in the MOVs caused by the reduced ionic radii of the MO dopants.

In ZnO-based MOVs, the nonlinear behavior is attributed to the presence of GBs in the polycrystalline ceramic materials. The GBs act as barriers for current flow and can be considered as a network of DSBs and/or ohmic contacts. The composition and distribution of metal impurities and dopants at the GBs affect the potential barrier height (Φ_B_) and width, which, in turn, determines the electrical properties of the varistors.

The difference between the ZnO-V_2_O_5_ and ZnO-Bi_2_O_3_ systems in GBs lies in the distribution of the dopant, either vanadium (V^5+^) or bismuth (Bi^3+^), within the ZnO-based varistor material. In the ZnO-V_2_O_5_ system, vanadium (V^5+^) is incorporated into the GBs in such a way that leads to the formation of donor states, since the ionic radius of V_2_O_5_ (0.054 nm) is lower than that of ZnO containing Zn^2+^ (0.06 nm) [145]. These donor states can trap electrons, leading to a nonlinear response of the ZnO-V_2_O_5_ varistor material to an applied voltage. On the other hand, in the ZnO-Bi_2_O_3_ system, bismuth (Bi^3+^) is distributed more uniformly throughout the GBs since the ionic radius of Bi_2_O_3_ (0.103 nm) is larger than that of ZnO containing Zn^2+^ (0.06 nm) [145]. Therefore, the resulting ZnO-Bi_2_O_3_ varistor material has a different nonlinear response.

The common GB structure observed in ZnO-Bi_2_O_3_-based varistors with 0.5–1 mol.% Bi_2_O_3_ content consists of a Bi-rich layer at the GBs, which represents the depletion layer (insulating layer) between the ZnO grains. The Bi-rich phases are located at the GBs and at the triple junctions of ZnO grains. The Bi-rich layer can increase the density of state at the GBs, thereby enhancing the nonlinearity of the ZnO-Bi_2_O_3_-based varistor ceramics [6,29,31,59,117,119,120,146,147]. Other GB structures that can be noticed in ZnO-Bi_2_O_3_-based varistors involve (i) the Bi_2_O_3_-ZnO eutectic structure observed at low Bi_2_O_3_ content, (ii) the ZnO-Bi_2_O_3_ solid solution observed at intermediate Bi_2_O_3_ content, and (iii) the ZnO-Bi_2_O_3_ peritectic structure noticed at high ZnO content [59]. These GB structures can act as barriers to the movement of charge carriers, resulting in an increase in the breakdown voltage (V_B_) of the varistors [114].

The regular GB structure observed in ZnO-V_2_O_5_ varistors is the zigzag structure, which consists of alternating planes of ZnO and V_2_O_5_ atoms, creating a zigzag pattern [134,135,136]. Other types of GB structures that can be observed in ZnO-V_2_O_5_-based varistors are (i) a disordered GB structure with a disordered arrangement of ZnO and V_2_O_5_ atoms, resulting in a lack of periodicity; (ii) an amorphous GB structure, where the atoms are arranged in a non-crystalline manner, and (iii) a segregated GB structure, where the ZnO and V_2_O_5_ atoms are separated into distinct regions, instead of being mixed together, resulting in a depletion layer at the GBs [27,36,56].

Depending on the structure and crystallographic orientation of grains, GBs that can be noticed in ZnO-based varistors, which are polycrystalline ceramic materials, can be planar (flat) GBs; symmetrical or asymmetrical tilt, twist, or mixed GBs, and faceted GBs [148,149], having different effects on the varistor properties. However, the nature and distribution of the GB structures mentioned above depend on the processing conditions and the specific dopants used. They can significantly impact the electrical properties of MOVs.

### 3.4. Inversion Boundary (IB)-Induced Grain Growth in ZnO-Based Systems

To control the ZnO grain growth during sintering and improve the performance of MOVs, binary ZnO-Bi_2_O_3_ systems are doped with grain growth modifiers or spinel-forming dopants such as Sb_2_O_3_, SnO_2_, TiO_2_, In_2_O_3_, Mn_3_O_4_, Al_2_O_3_, and Sb_2_O_5_, among others, with oxidation states (+III, +IV, +V) greater than +II of ZnO with Zn^2+^ ions [47,55,59,148,149,150]. These dopants enhance the donor states and reduce the barrier height at the GBs, which further improves the electrical performance of MOVs. The introduction of spinel-forming dopants into the ZnO crystal structure results in growth defects, such as inversion boundaries (IBs), which show inverted c-axis polarity across distinct crystal planes in ZnO grains [47,55,59,148,151]. Nonetheless, IBs do not form in pure ZnO crystal [55].

Conventionally, the positive polar [0001]-axis (c-axis) direction is from negative charges (O^2−^ ions) to positive charges (Zn^2+^ ions). The (0001) surface with an outer layer of Zn^2+^ ions is defined as the head termination, whereas the (0001¯) surface with an outer layer of O^2−^ ions is defined as the tail termination [151].

Inversion after ZnO crystal growth in the [0001] direction generates a head-to-head (H-H) IB, while that in the [0001¯] direction generates a tail-to-tail (T-T) IB [151].

H-H (→|←) (C^+^|C^+^) IBs and T-T (←|→) (C^−^|C^−^) IBs are mainly oriented across the basal planes (b-IBs) of the ZnO structure [151]. However, dopants such as Ga_2_O_3_ with Ga^3+^ ions [152] create inversion twins along the pyramidal planes (p-IB) of the wurtzite ZnO crystal structure.

**Figure 7:**
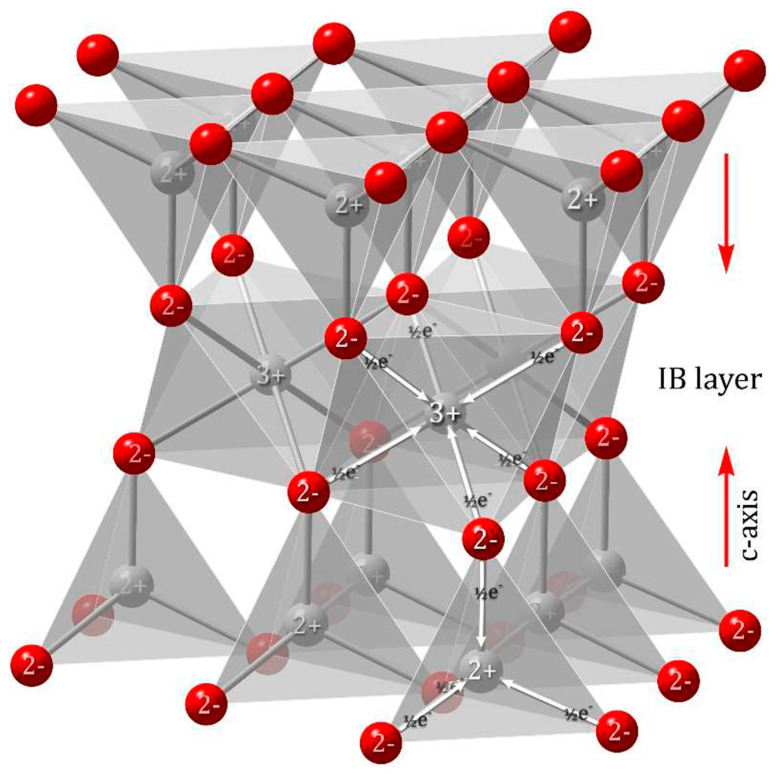
Schematic view of the charge balance model for an IB layer in the ZnO crystal. It illustrates the charge contributions from the O atoms (represented by red spheres) in tetrahedral and octahedral sites, resulting in +II and +III oxidation states, shown by white arrows. The red arrows indicate the polar c-axis orientations across the IB layer. Reprinted from reference [153], published under an open access Creative Commons Attribution (CC BY 4.0) license (https://creativecommons.org/licenses/by/4.0/ (accessed on 31 January 2023)). © 2021 The Authors.

H-H IBs are the most common type of IB found in ZnO and are formed by spinel-forming dopants. These dopants have an octahedral layer, an in-plane arrangement that complies with Pauling’s principle of electroneutrality in ionic crystals, where the mean charge of metal (M) cations is 3+ (Figure 7) [59,153]. The H-H-oriented IBs are related to the ionicity of the dopants and the electron counting in ZnO [151]. The formation of planar defects such as stacking faults (SFs) induced by the IB-forming dopants at high temperatures depends on the oxidation state of the dopants. To create an SF in an hcp lattice, one must insert or remove one close-packed plane into the ZnO structure while preserving the local charge balance.

Researchers have reported two main IB nucleation mechanisms for ZnO-based varistors doped with IB-forming dopants [59,148]. The first IB nucleation mechanism occurs for dopants with a +III oxidation state and is governed by Zn vacancy (V_Zn_) diffusion. On the other hand, all IB-forming dopants exhibit the second IB nucleation mechanism, which is based on surface nucleation and growth. This mechanism is governed by the chemisorption of the IB-forming dopants on Zn-deficient (0001) basal surfaces of the wurtzite ZnO crystal structure [59].

The cation diffusion mechanism is specific to all MO (II) dopants that develop a solid solution with ZnO while preserving the stable ionic structure of the wurtzite ZnO crystal. The assimilation of +II dopant ions balances the local charge deficiency generated by the Zn vacancies (V_Zn_) formed during sintering.

The diffusion of isovalent dopants into the wurtzite ZnO crystal structure is mainly attributed to the self-diffusion and evaporation of Zn atoms at high temperatures. The evaporation of Zn atoms from the (0001)-Zn surfaces generates stable clusters of V_Zn_ [59], which facilitate the mobility of +III dopant ions into the hcp basal planes of the ZnO structure [59]. The excess of Zn^2+^ ions from the adjacent ZnO layer relocates from the type-I to the type-II tetrahedral sites, resulting in the formation of H-H IBs. The propagation of +III dopant ions along the parallel basal planes can cause the production of multiple parallel IBs and the inversion of the neighboring ZnO domain, following the cation diffusion mechanism. These findings have been reported by various researchers [59].

The presence of +IV and +V dopant ions in ZnO yields a higher charge surplus of Zn^2+^ ions compared to +III dopant ions. As a result, the octahedral sites from the centers of the V_Zn_ clusters on the (0001)-Zn surfaces are more prone to chemisorption of the IB-forming dopants, leading to the formation of a stable 2D surface compound. Zn^2+^ ions can jump to adjacent octahedral interstices that align along the Zn cp layers, creating mixed octahedral interstices with +IV or +V dopant ions, while the O sublattice remains unchanged. After the octahedral interstices are occupied, the wurtzite ZnO structure on the outer surface of the IB layer begins to crystallize in an inverted orientation against the base crystal [59]. An IB plane is made up of a close-packed layer of MO_6_ octahedra that can assimilate metal (M) cations of different oxidation states, provided that their mean charge is 3+ [152].

The solid-state reaction between ZnO and IB-forming dopants generates IBs at lower temperatures and activation energies than those of the spinel phase origination. Low content (e.g., 0.1 mol.%) of IB-forming dopants creates a small number of IB nuclei and a coarse-grained microstructure. Conversely, high content (≥2 mol.%) of IB-forming dopants creates an increased number of IB nuclei and a fine-grained microstructure [59].

IBs do not yield a significant charge barrier [151,154], but H-H b-IBs modify the surface ionic structures of ZnO grains at O-terminated planes on each side of the IB grains. The formed GBs greatly influence the resulting electrical potential barriers and ZnO-ZnO GB resistance to enhance the nonlinearity of MOVs [148,151].

The existence of IBs in commercial ZnO-based varistors results in enhanced varistor properties [151]. These IBs cross virtually every ZnO grain feature, predominantly the C^−^ planes in the GBs, and improve the I-V characteristics. The barrier effect noticed in C^−^|C^−^ bicrystals doped with one or two additives (Mn, Co, Ni, and Bi) depended on the type and content of additives, but the doped C^+^|C^+^ bicrystals yielded negligible barrier effects [151].

Some studies [47,59,148] have shown that spinel grains in ZnO doped with Sb_2_O_3_, SnO_2_, TiO_2_, or In_2_O_3_ do not have significant effects on the microstructure and final ZnO grain size. However, each spinel phase can influence ZnO grain growth at high IB-forming dopant/Bi_2_O_3_ ratios (e.g., Sb_2_O_3_/Bi_2_O_3_ ratio > 1 and TiO_2_/Bi_2_O_3_ ratio > 1.5) [47]. In addition, a certain amount of MO dopant can cause excessive ZnO grain growth and overgrown spinel particles governed by the IBs in the abnormally large grains in the ZnO matrix.

The effect of various IB-forming dopants is not yet fully understood. Higher reactivity between the Bi_2_O_3_-rich liquid phase and the solid ZnO phase in the vicinity of the IB-forming dopant can improve ZnO grain growth in ZnO-Bi_2_O_3_-based systems. Furthermore, the IB-forming dopant/Bi_2_O_3_ ratio determines the phase equilibrium and the temperature of Bi_2_O_3_-rich liquid-phase generation.

Nevertheless, the reviewed literature reports scarcely address the effect of IB-forming dopants in ZnO-V_2_O_5_-based systems.

Guo et al. [155] prepared ZnO-0.5 mol.% V_2_O_5_ varistors doped with 2 mol.% MnO_2_-0.1 mol.% Nb_2_O_5_-0.5 mol.% Bi_2_O_3_-x mol.% SbVO_4_ (x = 0.1, 0.3, 0.5, 0.7). They obtained SbVO_4_ powder by mechanically mixing Sb_2_O_3_ and V_2_O_5_ powders in a molar ratio of 1:1. The obtained powder mixture was calcined in air at 650 °C for 1.5 h to decrease the sintering temperature and to avoid the inhibitory effect of the Sb_2_O_3_ amorphous thin film on the ZnO grain growth via the generation of the Zn_7_Sb_2_O_12_ spinel phase and IBs. The MOVs sintered at 940 °C for 4 h exhibited a main phase of ZnO and secondary phases of Zn_2.33_Sb_0.67_O_4_ spinel and BiVO_4_. The MOVs with 0.3 mol.% SbVO_4_ yielded enhanced nonlinearity (*α* = 51, J_L_ = 13.4 μA/mm^2^, and E_B_ = 4.16 kV/cm). Guo et al. [155] ascribed the inhibition of the ZnO grain growth and good varistor properties to the Zn_2.33_Sb_0.67_O_4_ spinel particle pinning mechanism [150], which depends on Zener’s drag (pinning pressure) phenomenon, as detailed elsewhere [156]. However, the authors did not investigate the formation of IBs in the ZnO grains.

### 3.5. Conduction Mechanisms in ZnO-Based Systems

Many state-of-the-art literature reports comprehensively describe the physics of MOVs, involving conduction mechanisms in ZnO-Bi_2_O_3_-based varistors and physical processes related to their microstructure and performance [6,56,65,84,157,158].

The proposed conduction mechanisms consider the following models over time:

▪ space-charge-limited current in the Bi_2_O_3_-rich intergranular thin layer;
▪ tunneling through a thin layer at the GBs or ZnO homojunctions;
▪ tunneling through DSBs without or with ZnO-Bi_2_O_3_ heterojunctions generated by interface states or with the creation of holes (minority carriers) at the GBs;
▪ hole-induced electrical breakdown (EBD) with DSBs created by interface states and bulk traps;
▪ space-charge-induced current or bypass effect at ZnO-Bi_2_O_3_ heterojunctions.

The main conduction mechanisms in the ohmic region in ZnO-Bi_2_O_3_ and ZnO-V_2_O_5_ systems depend on the thermionic emission in electrical potential barriers (DSBs) [84].

Distinct complex defects constituted of MO additives and native defects such as Zn vacancies (V_Zn_) and oxygen interstitials (O_i_) can generate acceptor states under electrical stress, along with the intergranular layer, segregation of MO additives, and oxygen-excess point defects during the degradation phenomenon of MOVs. The diffusion of interstitials close to the GBs can occur. On the contrary, the trapping away of interstitials far from the GBs can occur during the cooling of ZnO-based systems. This phenomenon can have a significant impact on the stability of MOVs [159].

In Gupta and Carlson’s GB defect model [158], used to study the stability of MOVs, the positively charged Zn_i_ is the primary metastable mobile ion able to enter the GB interface to neutralize the negatively charged interfacial states, such as the acceptor species of V_Zn_ involved in DSB degradation. When the oxygen-related point defects (O_i_) migrate across the GB, they reduce the complex defect density at the GB region and degrade the acceptor-like interfacial states, which disrupts and decreases the DSB.

In the extended Gupta and Carlson GB defect model with an external DC bias voltage, the charged particles inside the intergranular layer can be activated beside the metastable ions in the depletion layer, moving along or against the direction of the electric field, contributing to the degradation of DSBs [84]. The lowered barrier height (Φ_B_) was attributed to the decrease in the interface state density (N_s_) or the increase in the donor density (N_d_) since Φ_B_ is proportional to $N_{s}^{2}/N_{d}$. The anomalous degradation phenomenon does not constantly occur in each degradation test of MOVs. The reason is that the content of MO additives used to dope ZnO-based varistors is a crucial factor in the formation of intergranular phases [84].

## 4. Preparation of MOV Powders and Disc-Shaped MOVs from ZnO-V_2_O_5_ Systems

### 4.1. Preparation of MOV Powders from ZnO-V_2_O_5_-Based Systems

The starting powders in the achievement of ZnO-V_2_O_5_ systems with single- or multicomponent MO additives via a solid-state method are high-purity crystalline powders with fine or ultra-fine particle sizes. In addition to MOs, specific metallic salts (e.g., manganese carbonate (MnCO_3_), magnesium acetate (Mg(Ac)_2_), among others) can be used in preparing MOV powders [16,25,35,109] via a liquid-phase method that involves precipitation and calcination.

Common dopants in ZnO-V_2_O_5_-based systems include REOs, such as Er_2_O_3_, Gd_2_O_3_, Dy_2_O_3_, Sm_2_O_3_, Y_2_O_3_, La_2_O_3_, Ce_2_O_3_, Tb_4_O_7_, etc.; TEOs, such as Co_2_O_3_, Co_3_O_4_, Cr_2_O_3_, MnO, MnO_2_, Mn_3_O_4_, Nb_2_O_5_, TiO_2_, etc., and other MOs (In_2_O_3_, Bi_2_O_3_, Sb_2_O_3_, SnO_2_, etc.).

MOV powders are usually prepared by ball milling in a liquid medium (e.g., distilled or deionized water, acetone, ethanol, etc.) using a low- or high-energy planetary ball mill. The grinding media (GM) consist of polypropylene (PP) bottles and zirconia balls, agate vials and agate balls, or stainless steel (SS) vials and SS balls.

The grinding balls typically have a diameter in the range of 5–25 mm. The weight ratio of balls to MOV powders (BPR) is varied from 1:1 to 16:1, depending on the weight of the balls and MOV powders and the capacity of the vial. The milling duration (MD) and rotational speed (RS) are mainly established experimentally (MD from a few hours to over 40 h and RS of 100–400 rpm).

The milled slurry is dried in air or under a vacuum at low temperatures (80–120 °C) for up to 24 h, depending on the amount of MOV powder. The dried MOV powders are deagglomerated and sieved and can be subjected to calcination in air at 400–600 °C, for dwell times of several hours, and at low heating/cooling rates (e.g., HR = CR = 2−5 °C/min).

After mixing the MOV powders with a liquid medium and a small amount (e.g., 0.5–5 wt.%) of organic additive (binders, dispersing agents, among others), drying and sieving them with a typical mesh size of ≤149 μm, the granulation of the MOV composite powders takes place. Binders are selected from polyvinyl alcohol (PVA) [15,16,25,38,83,128,160], polyvinyl butyral (PVB) [27,35,36,124,161,162,163], ethyl cellulose (EC) [37], acrylic latex polymers [164,165], etc. Dispersing agents are chosen from polyurethane polymers [108,109], carboxylic anionic surfactants (sodium or ammonium polyacrylate polymers), phosphate anionic surfactants, cationic surfactants (quaternary ammonium salts), nonionic surfactants [166], etc.

Spray drying an aqueous slurry comprising milled MOV powders and organic additives such as binders, lubricants, plasticizers, and deflocculants, and subsequently sieving to the desired fractions, allows for the mass production of granulated MOV composite powders [112].

Tables S1 and S2 present the relevant parameters for the preparation of MOV composite powders from ZnO-V_2_O_5_ systems. However, most state-of-the-art literature reports do not provide sufficient data for all parameters and processing conditions.

Kelleher et al. [167] prepared MOV powders in a vibratory mill (a vertical mill with a chamber capacity of 15 L) filled with cylindrical zirconia media of approximately 19 mm size. They homogenized and milled an aqueous slurry containing large amounts of MOV powders (e.g., 5 kg of ZnO and MO additives in 5 L of deionized water), homogenized and milled for 1−18 h. After the first milling step, the authors performed several additional technological steps, including an initial spray drying step, calcination in air, a second milling step (e.g., 6 h), and a second spray drying step [168].

Manufacturers do not disclose the recipes and conditions for preparing MOV powders on a large industrial scale in the literature reports or technical documentation, due to the protection of their intellectual property rights (IPRs).

### 4.2. Preparation of MOV Discs from ZnO-V_2_O_5_ Systems by Conventional PM Routes

**Figure 8:**
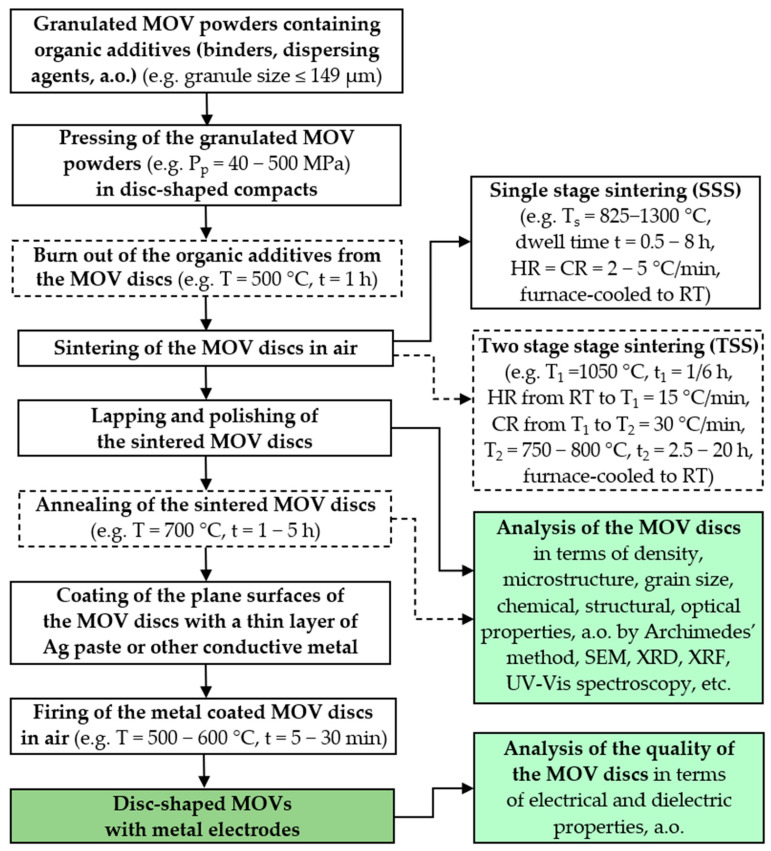
Technological flow chart for preparation of small-sized MOV discs by classical powder metallurgy (PM) methods.

Figure 8 illustrates the technological flow chart for the conventional preparation of small-sized MOV discs (Ø8–20 mm × 1–4 mm) for use in voltage SPDs.

The pressing pressures (P_p_) of the granulated MOV powders to obtain disc-shaped compacts typically range between 40 MPa and 500 MPa.

For sintering the green compacts, single-stage sintering (SSS) (Table S1) or two-stage sintering (TSS) (Table S2) is carried out in air, with a sintering temperature (T_s_) of 800–1300 °C for a dwell time of 0.5–8 h at T_s_, a low heating/cooling rate (HR/CR) of approximately 2–5 °C/min, and furnace cooling of the sintered MOVs to RT. Subsequently, the MOV discs are lapped and polished with SiC abrasive paper and diamond or Al_2_O_3_ suspensions containing fine abrasive particles and a cooling lubricant [35,63,169] to obtain flat surfaces with a parallel-plane configuration.

To prevent and reduce V_2_O_5_ volatilization loss during the sintering stage of ZnO-V_2_O_5_-based varistors, a specific quantity of V_2_O_5_ powder or a powder mixture of ZnO and V_2_O_5_ with slightly higher V_2_O_5_ content than that used for the MOV discs is placed in a double crucible inside the heat treatment furnace [37,170].

The TSS process follows similar steps to those of the SSS process. In the TSS process reported in [63], the first sintering temperature (T_1_ of 1050 °C) is higher than the second one (T_2_ = 750–800 °C), the dwell time (t_1_) at T_1_ is much lower (t_1_ = 1/6 h) than the dwell time (t_2_) at T_2_ (t_2_ = 2.5–40 h), the heating rate from RT to T_1_ is 15 °C/min, the cooling rate from T_1_ to T_2_ is 30 °C/min, and the sintered MOVs are furnace-cooled to RT. A TSS process with T_1_ < T_2_, or multistage sintering (MSS), is an alternative process for manufacturing MOVs.

The burnout of organic additives is typically performed during the sintering of the MOV discs or in a heat treatment carried out before sintering [25]. The temperature, dwell time, and heating rate to burn out the organic additives must be carefully selected and controlled since these parameters influence the densification, shrinkage rate, and the electrical performance (nonlinear coefficient, power loss, and energy absorption capability) of the MOVs [77,171]. Moreover, fast heating rates can affect the quality and functional performance of MOVs by potentially causing defects (microcracks, voids, and pores).

To improve the microstructure and electrical properties, annealing of the polished MOV discs is carried out (e.g., at 700 °C for 1–5 h) [35,125,170]. A conductive paste mainly based on Ag is used to cover the upper and lower plane surfaces of the MOV discs to metalize them. The coated MOVs are heated in air at 500–600 °C for 5–30 min to form Ag inner electrodes [63], which are necessary to assess the performance of MOVs. Each Ag electrode (top layer) can contain an intermediary Au layer deposited onto the plane surfaces of MOVs by magnetron sputtering [170]. Co-firing MOV discs from the ZnO-V_2_O_5_-based systems with Ag inner electrodes can be performed at a sintering temperature of 800–900 °C [60,61]. To prevent disruptive electric discharges, the plane surfaces of disc-shaped MOVs can be partially metallized, leaving a circular space around the edges left uncoated [3].

The main technological steps for conventional PM preparation of large-size MOV discs (diameter × height of Ø20–120 mm × 20–50 mm) for use in surge arresters for electric power systems are as follows [3,112]:

➢ Granulation of MOV powders containing organic additives (binders, lubricants, plasticizers, and deflocculants), which have been previously milled and spray-dried;
➢ Pressing of the granulated MOV powders into disc-shaped compacts;
➢ Burning out of the organic additives from the MOV discs;
➢ High-temperature sintering of the MOV discs in air;
➢ Glass coating on the cylindrical surfaces of the MOV discs for passivation;
➢ Glass firing of the coatings;
➢ Grinding of the contact surfaces of the MOV discs using a warm ultrasonic process and forced air convection;
➢ Metallization of the plane contact surfaces of the MOV discs with Al metal.

To achieve an accurate geometrical condition in the MOV discs, rigorous grinding of the contact surfaces of the MOV discs must be performed before the metallization stage to obtain flat surfaces with a parallel-plane configuration. If this condition is not fulfilled, various technical issues can occur during the service of gapless MO surge arresters (MOSAs) containing a stack of MOV discs mounted in series in a sealed housing made of porcelain or a silicone-rubber-based polymer. These issues include poor contact between two adjacent MOV discs and weak conduction paths across the stack of MOV discs.

## 5. Properties of Disc-Shaped MOVs from ZnO-V_2_O_5_ Systems

### 5.1. Structural Properties

The XRD analyses confirmed the crystalline nature of both the ball-milled MOV composite nanopowders and the conventionally sintered MOV discs selected for this review (see Tables S1 and S2). The XRD peak broadening of ZnO revealed the refinement of the ZnO grain size during the ball milling of the MOV composite powders for 8–35 h [169].

**Figure 9:**
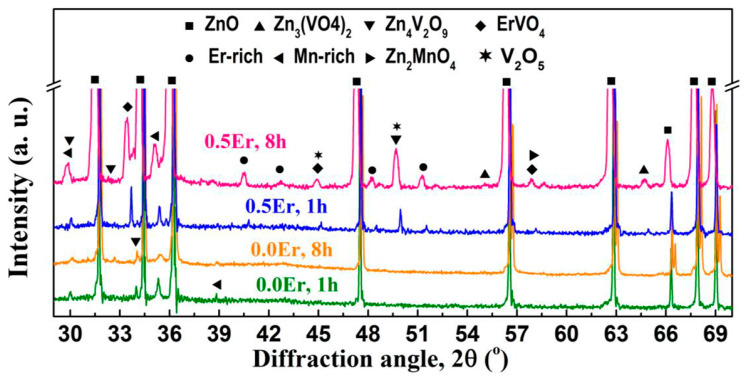
XRD patterns of MOVs (mol.%) from (97.4—x)% ZnO-0.5% V_2_O_5_-2% MnO_2_-0.1% Nb_2_O_5_, and x = 0 and 0.5 mol.% Er_2_O_3_ systems sintered at 1100 °C for 1 h and 8 h. Reprinted from reference [62], published under an open access Creative Commons Attribution (CC BY 3.0) license (https://creativecommons.org/licenses/by/3.0/ (accessed on 14 October 2022)) in IOP Conf. Ser. Mater. Sci. Eng. by IOP Publishing Ltd.

All the ball-milled MOV powders and disc-shaped MOVs sintered in air at different temperatures (825–1300 °C) for 0.5–8 h (in SSS) or ≤40 h (in TSS) contained a primary crystalline phase of ZnO with a hexagonal wurtzite structure and several secondary crystalline phases. The formation of secondary phases depended on the chemical composition (in mol.%) of the prepared MOVs and the variation in PM parameters, as follows:

➢ Zn_3_(VO_4_)_2_ (β phase or β + γ phases) [30,36,37,144] (chemical reaction (2) [124]) was observed in ZnO-V_2_O_5_ and ZnO-V_2_O_5_-MnO_2_ systems with 0.25–2% V_2_O_5_ and 0.5–2% MnO_2_ sintered at 900–1100 °C for 2–4 h:
  3ZnO + V_2_O_5_ → Zn_3_(VO_4_)_2_ (2)
➢ Zn_3_(VO_4_)_2_, ZnV_2_O_4_, and VO_2_ phases [27,38,50,124,144,162,172] (chemical reactions (3) and (4) [124]) were observed in ZnO-V_2_O_5_-Mn_3_O_4_ and ZnO-V_2_O_5_-MnO_2_-Nb_2_O_5_ systems with 0.5% V_2_O_5_, 0.5–2% Mn_3_O_4_, or MnO_2_ and 0.05–0.1% Nb_2_O_5_ sintered at 825–900 °C for up to 4 h. The MOVs sintered at 875 °C exhibited a supplementary Mn-rich phase [163]:
  ZnO + V_2_O_5_ → ZnV_2_O_4_ + O_2_ (3)
  V_2_O_5_ → 2VO_2_ + (1/2)O_2_ (4)
➢ Zn_3_(VO_4_)_2_, Zn_4_V_2_O_9_, or ZnV_2_O_4_, unreacted V_2_O_5_ or VO_2_, Mn-rich, and ErVO_4_, DyVO_4_, GdVO_4_, YbVO_4_, BiVO_4_, or TbVO_4_ phases (chemical reactions (2)–(7), where M = Er, Dy, Gd, Yb, or Bi), were observed in ZnO-V_2_O_5_-MnO_2_-Nb_2_O_5_ systems with 0.5% V_2_O_5_, 2% MnO_2_, and 0.05–0.1% Nb_2_O_5_ doped with up to 0.25% Er_2_O_3_ [124,163], Dy_2_O_3_ [24], Gd_2_O_3_ [173], Yb_2_O_3_ [27], Bi_2_O_3_ [172], or Tb_4_O_7_ [162] and sintered at 875–1300 °C for 0.5–8 h:
  4ZnO + V_2_O_5_ → Zn_4_V_2_O_9_ (5)
  V_2_O_5_ + M_2_O_3_ → 2MVO_4_ (6)
  2V_2_O_5_ + Tb_4_O_7_ → 4TbVO_4_ + (1/2)O_2_ (7)
➢ Zn_3_(VO_4_)_2_ and ErVO_4_ phases [161] were observed in ZnO-V_2_O_5_-Mn_3_O_4_-Nb_2_O_5_-Er_2_O_3_ systems with 0.5% V_2_O_5_, 0.5% Mn_3_O_4_, 0.05% Nb_2_O_5_, and 0.05% Er_2_O_3_ sintered at 925–950 °C for 3 h.
➢ Zn_3_(VO_4_)_2_, Zn_4_V_2_O_9_, V_2_O_5_, Mn-rich, Er-rich, and ErVO_4_ phases [62,63,169,174,175] were observed in ZnO-V_2_O_5_-MnO_2_-Nb_2_O_5_-Er_2_O_3_ (ZVMNE) systems with 0.5% V_2_O_5_, 2% MnO_2_, 0.05–0.1% Nb_2_O_5_, and 0.5–2% Er_2_O_3_ sintered at 900–1300 °C for 0.5–8 h. The MOVs sintered at 950–1100 °C for ≤8 h also yielded the Zn_2_MnO_4_ phase [62,175]. Figure 9 depicts the XRD patterns of the ZVMNE systems developed by Roy et al. [62].
➢ Zn_3_(VO_4_)_2_, Zn_2_V_2_O_7_, Zn(VO_3_)_2_, InVO_4_, and Zn_7_I_2_O_10_ phases (chemical reactions (6) and (8)–(10), where M = In) were observed in ZnO-V_2_O_5_-Nb_2_O_5_-In_2_O_3_ systems with 0.5% V_2_O_5_, 1% Nb_2_O_5_, and 0.1% In_2_O_3_ sintered at 875–900 °C for 1 h. No secondary phases related to Nb were found, which could be attributed to the possible incorporation of Nb into the ZnO solid solution [44].
  2ZnO + V_2_O_5_ → Zn_2_V_2_O_7_ (8)
  ZnO + V_2_O_5_ → Zn(VO_3_)_2_ (9)
  7ZnO + In_2_O_3_ → Zn_7_I_2_O_10_ (10)
➢ Zn_3_(VO_4_)_2_, ZnV_2_O_4_, and ZnMn_2_O_4_ phases were observed in ZnO-V_2_O_5_-MnCO_3_-Nb_2_O_5_ systems with 0.5% V_2_O_5_, 2% MnCO_3_, and 0.1% Nb_2_O_5_ sintered at 900–930 °C for 3 h. Doping this system with 0.05 mol.% Y_2_O_3_, Nd_2_O_3_, or Sm_2_O_3_ resulted in the formation of an additional phase (YVO_4_, NdVO_4_, or SmVO_4_) [25] (chemical reaction (6), where M = Y, Nd, or Sm).
➢ Zn_3_(VO_4_)_2_, Zn_4_V_2_O_9_, ZnV_2_O_4_, V_2_O_5_, and Zn_2_MnO_4_ phases were observed in ZnO-V_2_O_5_-MnO_2_-Nb_2_O_5_ systems with 0.5% V_2_O_5_, 2% MnO_2_, and 0.1% Nb_2_O_5_ sintered by SSS at 1100 °C for 0.5–8 h or TSS (T_1_ = 1050 °C, t_1_ = 1/6 h, T_2_ = 750–800 °C, t_2_ = 2.5–40 h). Additionally, an ErVO_4_ phase was formed in 0.5% Er_2_O_3_-doped systems [62,63].

The structure of the MOVs from the ZnO-0.5–2 mol.% V_2_O_5_-based systems doped with different MOs or their compounds consists of a primary ZnO phase with a hexagonal wurtzite structure and several secondary phases. The identified phases are highly dependent on the nature and content of each MO, as well as the sintering temperature and dwell time (Tables S1 and S2).

A few studies have reported three polymorphs (α, β, and γ) of the secondary Zn_3_(VO_4_)_2_ phase, which undergo a slow and reversible transformation at 795 °C between α and β, and a rapid and reversible transformation at 815 °C between β and γ [30,38]. However, Kurzawa et al. [126] reported that the Zn_3_(VO_4_)_2_ phase does not exhibit the polymorphism phenomenon, but instead, at 800 °C, it changes into a solid phase to produce β-Zn_2_V_2_O_7_ and Zn_4_V_2_O_9_. A reversible transformation between α-Zn_2_V_2_O_7_ and β-Zn_2_V_2_O_7_ occurs at approximately 590–620 °C, and Zn_2_V_2_O_7_ melts evenly at around 872–890 °C, while Zn_4_V_2_O_9_ melts unevenly at 910 °C [126].

Hng et al. [18] identified, by XRD analysis, α-, β-, and γ-Zn_3_(VO_4_)_2_ phases in the structure of MOVs from undoped or 1 mol.% MnO_2_ or Co_3_O_4_-doped ZnO-0.25 mol.% V_2_O_5_ systems sintered in air at 900 °C for 4 h. In the α-Zn_3_(VO_4_)_2_ phase, they determined through EDS analysis a proper Zn to V atomic ratio of 3:2. In the β-Zn_3_(VO_4_)_2_ phase, they found a Zn to V atomic ratio of approximately 2:1, which is similar to that of the metastable Zn_4_V_2_O_9_ phase [176]. The γ-Zn_3_(VO_4_)_2_ phase was considered an oxide comprising Zn, V, and Mn elements, or a zinc vanadate with a Zn to V atomic ratio of around 1:1, prone to developing a solid solution with Mn.

### 5.2. Grain Growth Behavior, Densification, and Microstructure of ZnO-V_2_O_5_-Based Systems

The ZnO grain growth behavior during liquid-temperature sintering (LPS) in air of the ZnO-V_2_O_5_-based systems at temperatures over 800 °C is highly dependent on the sintering temperature, dwell time, and the content of V_2_O_5_ and MO additives.

The grain growth kinetics during LPS of MOVs from the ZnO-based systems is determined by Equation (11) [21,83,177,178]:

(11) $$G^{n}-G_{0}^{n}=K_{0}\cdot t\cdot exp\left(-\frac{Q}{RT}\right)$$

where *G* is the mean ZnO grain size at the sintering time *t*, *G_0_* is the initial ZnO grain size, *n* is the kinetic grain growth exponent, *Q* is the apparent activation energy for grain growth, K_0_ is the preexponential constant of the ceramic material, *R* is the universal gas constant, and *T* is the absolute temperature (T = 300 K).

Usually, *G*_0_ = 0 when *G* >> *G*_0_, and the kinetic grain growth exponent (*n*) is estimated from the slope of the plots of lg*G* versus lg*t*, which is equal to 1/n [21,83,177,178]:

(12) $$\mathrm{lg}G=\frac{1}{n}\mathrm{lg}t+\frac{1}{n}\left[\mathrm{lg}K_{0}-0.434\left(\frac{Q}{RT}\right)\right]$$

The apparent activation energy for grain growth (*Q*) is set from Equation (13) [21,83,177,178] (*Q* is estimated from the slope of Arrhenius plots of lg(*G^n^/t*) versus 1/*T*):

(13) $$\mathrm{lg}\left(\frac{G^{n}}{t}\right)=\mathrm{lg}K_{0}-\frac{0.434Q}{R}\left(\frac{1}{T}\right)$$

The mean size of ZnO grains (referred to as d) in MOVs (Table S1) was determined by using the linear intercept method with SEM images, as shown in Equation (14) [38]:

(14) $$d=1.56L/\left(M\cdot N\right)\;$$

where L is the length of a random straight line drawn on an SEM image, M is the magnification of the SEM image, and N is the number of GBs intercepted by the straight line.

The density of disc-shaped MOVs was determined by all authors of the studies presented in this review using Archimedes’ method with a hydrostatic balance and distilled water as the immersion liquid, in accordance with standard ISO 18,754 specific to advanced ceramics. Relative density (RD) can be calculated as the ratio of achieved density to theoretical density (TD).

According to Hng et al. [177], the binary ZnO-V_2_O_5_ (ZV) systems with 0.5–2 mol.% V_2_O_5_ follow a grain growth mechanism that is governed by a phase-boundary reaction. Increasing the V_2_O_5_ content causes the ZnO grain size to increase, but the thickness of the liquid phase also affects grain growth. In the ZV systems with 4 mol.% V_2_O_5_, the grain size decreased due to the occurrence of diffusion through the intergranular liquid layer. Adding V_2_O_5_ to the ZnO matrix decreased the grain growth exponent (n) and apparent activation energy (Q) of the ZV systems, compared to those of pure ZnO ceramics (n = 3, and Q = 224 ± 16 kJ/mol). As the V_2_O_5_ content increased, the n value varied from 1.52 to 2.69, and the Q value was ~88 kJ/mol for ZV systems with 0.5–2 mol.% V_2_O_5_, and 115 ± 24 kJ/mol for ZV systems with 4 mol.% V_2_O_5_.

The above findings suggest a faster ZnO grain growth rate during the sintering of the ZnO-V_2_O_5_ systems compared to that of pure ZnO ceramics, where the grain growth mechanism involves the solid-state diffusion of Zn^2+^ ions into the ZnO lattice.

In a study by Tsai et al. [170], it was revealed that the grain growth of undoped ZnO changed from isotropic to anisotropic when 0.25–2 mol.% V_2_O_5_ was added to ZnO ceramics and sintered in air at 900 °C for 0.5–8 h. The growth rate of ZnO grains and the thickness of the secondary V_2_O_5_-related phase located at the GBs increased with the increase in V_2_O_5_ content due to the V_2_O_5_-rich liquid phase during sintering at 900 °C [170].

**Figure 10:**
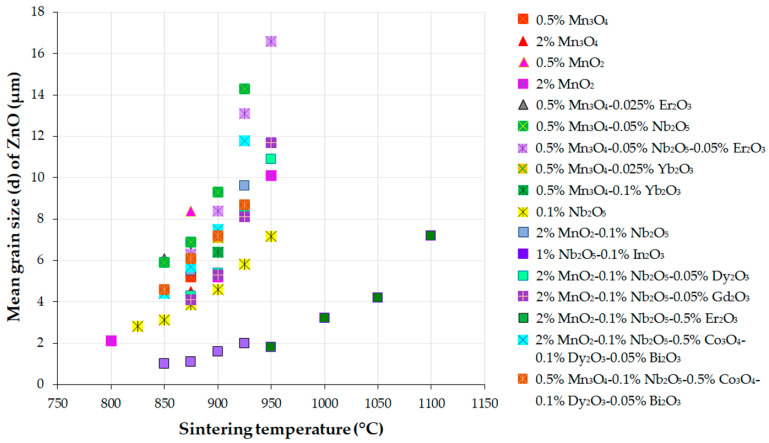
Variation in the mean ZnO grain size with the sintering temperature of MOVs from the ZnO-0.5 mol.% V_2_O_5_-based systems doped with various MOs (mol.%). The data plotted in the figure were collected from references [27,36,44,50,60,144,161,173,174,179,180,181,182,183].

**Figure 11:**
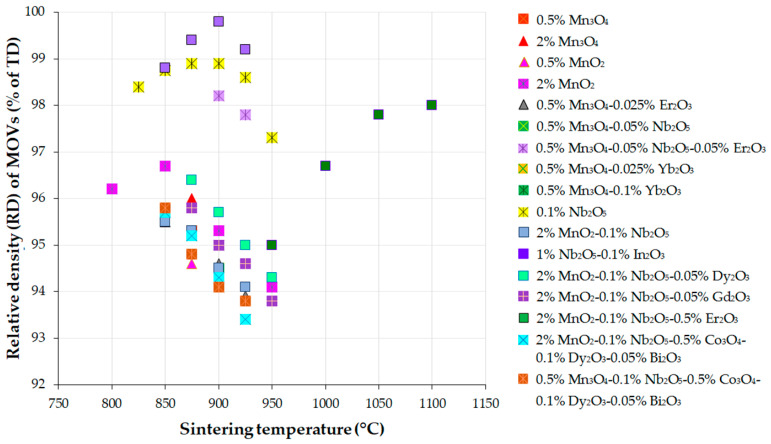
Variation in the relative density (RD) with the sintering temperature of MOVs from the ZnO-0.5 mol.% V_2_O_5_-based systems doped with various MOs (mol.%). The data plotted in the figure were collected from references [27,36,44,50,60,144,161,173,174,179,180,181,182,183].

The variation in mean ZnO grain size (d_ZnO_) and density with increasing sintering temperature was commonly reported for ZnO-V_2_O_5_-based systems (Figure 10 and Figure 11) [36,38,161,169,174], as well as for ZnO-Bi_2_O_3_-based systems [6] produced via the conventional powder metallurgy (PM) route.

Nahm [144] investigated the effect of Mn ions (Mn^2.66+^ from Mn_3_O_4_ and Mn^4+^ from MnO_2_) on ZnO-0.5 mol.% V_2_O_5_ systems doped with 0.5−2 mol.% Mn, which were sintered in air at 900 °C for 3 h. Low Mn content (0.5 mol.%) resulted in nonuniform ZnO grain growth, while higher Mn content (2 mol.%) improved the uniformity and decreased irregular grain growth. Mn doping reduced the mean ZnO grain size in all the ternary systems compared to undoped MOVs from ZnO-V_2_O_5_ systems, but the densification of the Mn_3_O_4_- or MnO_2_-doped MOVs was almost equal, with a relative density (RD) of 94.6–96% of theoretical density (TD). These densities comply with the accepted values for commercial MOVs, with RD greater than 95% of TD, where TD is around 5.6–5.7 g/cm^3^ [111].

The mean ZnO grain size of the 0.5 mol.% Mn_3_O_4_ or 2 mol.% MnO_2_-doped ZnO-0.5 mol.% V_2_O_5_ systems increased with an increase in sintering temperature from 800 °C to 950 °C, but decreased between 875 °C and 950 °C. MnO_2_-doped MOVs yielded a higher relative density (96.7% of TD) [36] than Mn_3_O_4_-doped MOVs (94.3% of TD) [38]) at lower sintering temperatures (800–850 °C). Nahm [38] attributed this finding to the Zn_3_(VO_4_)_2_ secondary phase formed in the ZnO matrix, which acted as a liquid-phase sintering aid from 800 °C to 850 °C. The decrease in the distribution of this secondary phase at GBs at higher sintering temperatures (875–950 °C) reduced the sintered density.

Several studies have shown that the ZnO grain size increases linearly with increasing sintering temperature for MOVs (mol.%) from ZVN systems doped with 0.1–1 mol.% Nb_2_O_5_ [28] sintered in air at 850–975 °C for 1 h, ZVN systems doped with 0.5 mol.% Mn_3_O_4_ [50], or 0.05 mol.% Er_2_O_3_ [161] sintered in air at 875–950 °C for 1 h, as well as for undoped ZVMN systems and those doped with 0.5–2 mol.% Er_2_O_3_ (ZVMNE) systems sintered in air at 900 °C to 1300 °C [169,174], where the Nb_2_O_5_ content was 0.05–0.1 mol.% and MnO_2_ content was 2 mol.%.

The mean ZnO grain size increase was approximately 3.4–11.8 times more significant for the ZVMN systems sintered at high temperatures (1100–1300 °C). The addition of 0.5 mol.% Er_2_O_3_ considerably decreased the mean ZnO grain size in ZVMNE systems by approximately 36–67% for MOVs sintered at 950–1100 °C. Both ZVMN- (RD of 97.5–99.4%) [174] and ZVMNE-based MOVs (RD of 95–98%) [169] exhibited improved densification. The highest densities were achieved at the optimal sintering temperature of 1100 °C.

The ZVN systems with 0.1–1 mol.% Nb_2_O_5_ [28] sintered at 850–975 °C yielded a high relative density (RD of 97.3–98.9%). MOV doping with Er_2_O_3_ exhibited a higher relative density (98.9%) [161] than Mn_3_O_4_-doped MOVs (95.8%) [50], but showed a decrease in density with increasing sintering temperature.

**Figure 12:**
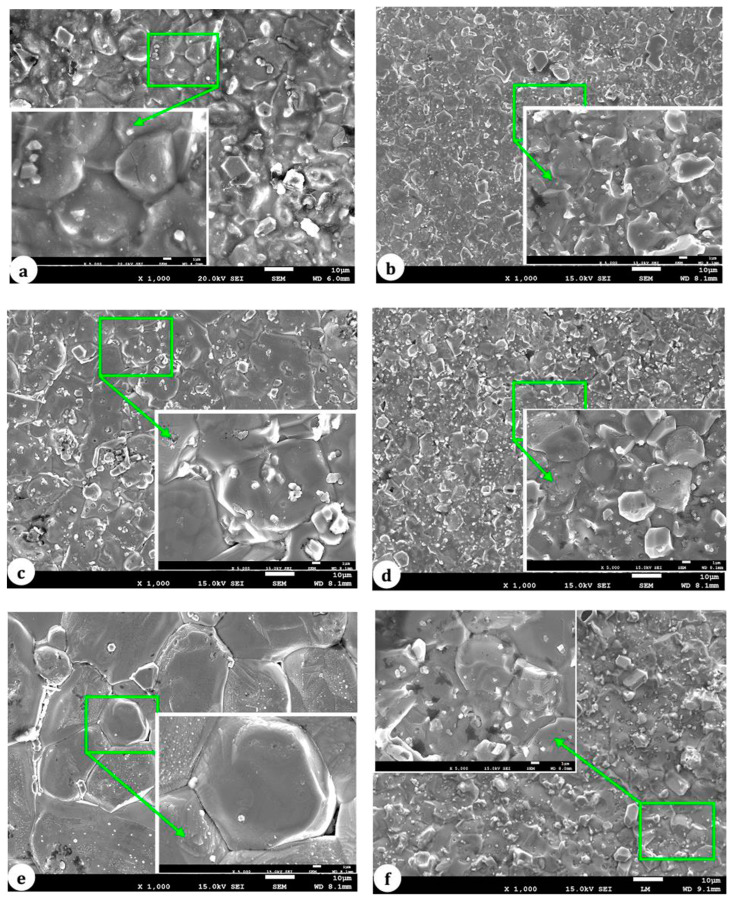
FESEM images (×1000) of MOVs (mol.%) from the ZVMN systems sintered at 1100 °C for (**a**) 0.5 h, (**c**) 2 h, (**e**) 8 h, and 0.5 mol.% Er_2_O_3_-doped ZVMN systems sintered at 1100 °C for (**b**) 0.5 h, (**d**) 2 h, (**f**) 8 h (inset images ×5000). Reprinted from reference [62], published under an open access Creative Commons Attribution (CC BY 3.0) license (https://creativecommons.org/licenses/by/3.0/ (accessed on 14 October 2022)) in IOP Conf. Ser. Mater. Sci. Eng. by IOP Publishing Ltd.

Figure 12 illustrates FESEM images of typical MOVs (mol.%) from the undoped ZnO-0.5% V_2_O_5_-2% MnO_2_-0.1% Nb_2_O_5_ (ZVMN systems) and 0.5 mol.% Er_2_O_3_-doped ZVMN systems sintered in air at 1100 °C for 0.5 h, 2 h, and 8 h [62]. The figure shows the gradual increase in grain growth with increasing dwell time from 0.5 h to 8 h, and a larger mean ZnO grain size for the undoped ZVMN systems (32.6·µm) compared to the Er_2_O_3_-doped ones (10.8·µm).

The boundary areas, as well as the triple or multiple grain junctions (TGJs or MGJs), mostly contain the Zn_3_(VO_4_)_2_, Zn_2_V_2_O_7_, Zn_4_V_2_O_9_, Mn-, and RE-related phases, as shown in Figure 12 and confirmed by EDS analysis of MOVs [25,63,169].

Doping ZVN systems with 0.1 mol.% In_2_O_3_ (ZVNI systems) inhibited grain growth at sintering temperatures of 850–925 °C [44] and improved the relative density of MOVs to 98.8–99.8%. The mean ZnO grain size of In_2_O_3_-doped MOVs decreased by around 35–43% compared to undoped MOVs. The volumetric shrinkage of green compacts increased linearly from approximately 30% to 35% for MOVs sintered at 850–925 °C, achieving nearly fully dense MOVs (RD ≥ 98.9%) [44]. Sintering at higher temperatures (925–975 °C) led to a decrease in shrinkage from 35% to 31% with increasing sintering temperature, but hindered the densification of MOVs. The volatility of V-related species resulted in material loss and reduced density [25,28,36,44,160,169].

Usually, during conventional sintering in air at 800–1300 °C, MOVs experience dimensional shrinkage of 10–35%, which increases as the sintering temperature rises [28]. One practical approach to reduce the shrinkage rate is to use rate-controlled sintering (RCS) to burn out the organic additives used to prepare granulated MOV powders [77].

RCS can also modify the phase distribution in the microstructure of MOVs, reduce their porosity, and change the pore distribution to improve the nonlinear coefficient, power loss, and energy absorption capability [77].

**Figure 13:**
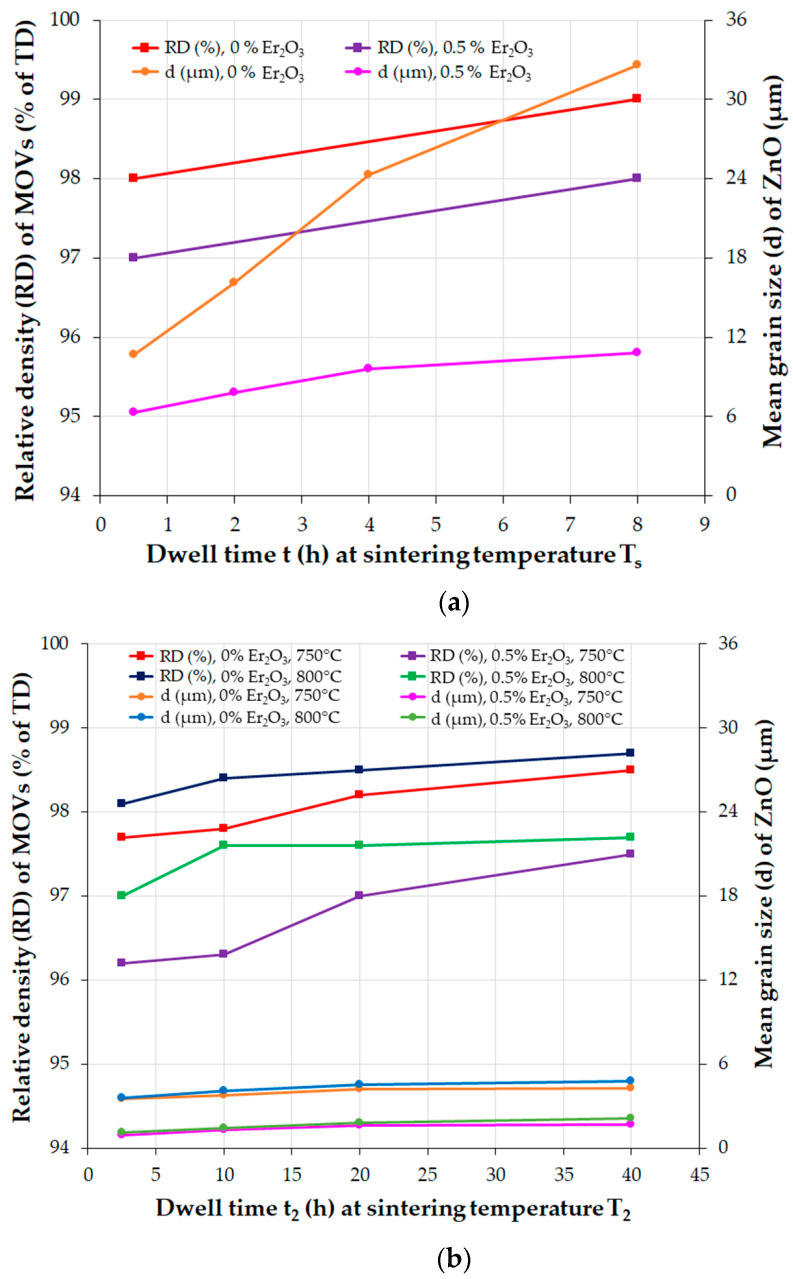
Variation in the relative density (RD) and mean ZnO grain size (d) of the MOVs (mol.%) from the ZnO-0.5% V_2_O_5_-2% MnO_2_-0.1% Nb_2_O_5_ systems with 0-0.5% Er_2_O_3_ prepared by (**a**) SSS process (T_s_ = 1100 °C, t = 0.5–8 h); (**b**) TSS process (T_1_ = 1050 °C, t_1_ = 1/6 h, T_2_ = 750 °C or 800 °C, t_2_ = 2.5–40 h). The data plotted in the figure were collected from references [62,63].

Roy et al. [63] used a TSS process in air (T_1_ = 1050 °C, t_1_ = 1/6 h, T_2_ = 750 °C or 800 °C, t_2_ = 2.5–40 h) to prepare ZnO-0.5 mol.% V_2_O_5_-2 mol.% MnO_2_-0.1 mol.% Nb_2_O_5_ (ZVMN) systems with or without 0.5 mol.% Er_2_O_3_ doping. The mean ZnO grain size slightly increased for undoped MOVs, while the Er_2_O_3_-doped MOVs (ZVMNE system) exhibited a reduction in the mean ZnO grain size of about 26–40% at T_2_ = 750 °C, and 31–44% at T_2_ = 800 °C (Figure 13). Nevertheless, increasing the second-stage sintering parameters (T_2_ and t_2_) had an insignificant effect on the density of all the synthesized MOVs.

The TSS process was found to yield high-density MOVs from both undoped and Er_2_O_3_-doped ZVMN systems [63] (RD of 97.7–98.7%, as shown in Figure 13). The microstructure of these MOVs was fine-grained, with a lower ZnO grain size compared to those of MOVs with the same formulation achieved by SSS [62] (Tables S1 and S2). However, a drawback of the utilized TSS process is the extended duration required for the second dwell time (t_2_ = 10–40 h), which could increase the production costs of MOVs due to higher energy consumption than with a sintering time of less than 10 h.

**Figure 14:**
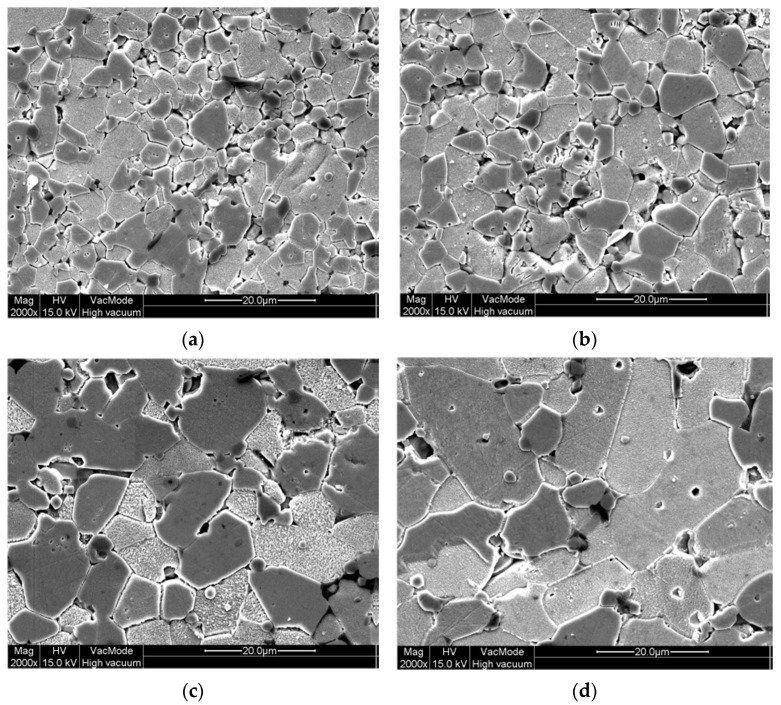
SEM images (×2000) of MOVs (mol.%) from the ZVMND systems sintered at (**a**) 875 °C, (**b**) 900 °C, (**c**) 925 °C, and (**d**) 950 °C, etched in a HClO_4_-H_2_O solution (1:1000, *v*/*v*) for 25 s at RT. Reprinted from reference [181], published under an open access Creative Commons Attribution (CC BY 3.0) license (https://creativecommons.org/licenses/by-nc/3.0/ (accessed on 18 October 2022)). Copyright © 2015 KIEEME.

Nahm [181] observed an increase in ZnO grain growth with an increase in sintering temperature for the ZnO-0.5% V_2_O_5_-2% MnO_2_-0.1% Nb_2_O_5_-0.05% Dy_2_O_3_ (ZVMND) systems (mol.%) sintered in air at 875–950 °C. The SEM images of the ZVMND systems (Figure 14) display the typical fine-grained microstructures of MOVs for HV applications, with comparable surface morphologies to other ZnO-V_2_O_5_-based varistors [24,27,180]. The microstructures of all MOVs showed clear GBs and irregular grains of various sizes. The increase in temperature caused a gradual change in the ZnO grain size, with the grain sizes significantly increasing when the sintering temperature exceeded 925 °C.

Zhao et al. [160] investigated the effect of 0.1 mol.% Ce–La doping on the ZnO-0.5% V_2_O_5_-2% MnCO_3_-0.1% Nb_2_O_5_ (ZnVMnNbO) systems sintered in air at 850–925 °C. The Ce-La-doped systems showed a slight decrease in the mean ZnO grain size (d_ZnO_ of 3.8–7.3 µm) with the increase in sintering temperature and higher densities (RD of 97.5–97.6%) compared to the undoped MOVs (d_ZnO_ of 4.3–7.4 µm). The MOVs sintered at temperatures of 900–925 °C achieved the highest densities.

The sintering mechanism of the undoped ZnVMnNbO varistors is based on the reaction at the phase boundaries among ZnO grains (solid state) and liquid phases related to V-rich and MnZn_2_Nb_2_O_8_, promoting the sintering of MOVs, combined with the pinning of ZnO grain growth by the ZnMn_2_O_4_ spinel particle phase [160]. This finding is also reported in ZnO-Bi_2_O_3_-based varistors containing spinel-forming dopants [28,30,169].

The Ce-La-doped ZnVMnNbO varistors followed a sintering mechanism equivalent to the undoped ones. The formed Ce(La)VO_4_ intergranular particle phase and the decrease in the V-rich phase also inhibited ZnO grain growth. The kinetics study [160] showed that the grain growth exponent (n) and apparent activation energy (Q) of the undoped MOVs (n = 2.39, Q = 186 KJ/mol) increased for the Ce-La-doped ZnVMnNbO varistors (n = 2.95, Q = 263 KJ/mol), making it more difficult to sinter the Ce-La-doped MOVs.

In the binary ZnO-0.5 mol.% V_2_O_5_ systems doped with 0.05–0.5 mol.% Nb_2_O_5_ or 0.1 mol.% Nb_2_O_5_ + 2 mol.% MnCO_3_ [125] sintered in air at 900 °C for 3 h, anisotropic ZnO grain growth was observed, which increased with increasing Nb content. The density of the doped MOVs (RD of 94.7–96.84%) decreased with increasing Nb content.

The addition of up to 0.25 mol.% REO (Er_2_O_3_ or Yb_2_O_3_) to ZnO-0.5 mol.% V_2_O_5_-0.5 mol.% Mn_3_O_4_ (ZVM*) systems sintered in air at 900 °C for 3 h hindered grain growth and improved densification with increasing REO content [27,184]. The selected REOs acted as efficient grain growth inhibitors and densification enhancers.

The introduction of up to 0.25 mol.% Bi_2_O_3_ [172] or an REO (Dy_2_O_3_ or Tb_4_O_7_) [27,162] into ZnO-0.5 mol.% V_2_O_5_-2 mol.% MnO_2_-0.1 mol.% Nb_2_O_5_ (ZVMN) systems sintered in air at 900 °C for 3 h contributed to a slight variation in the mean ZnO grain size, compared to the undoped MOVs, and a low variation in density (RD of 93.9–96.5%). The density of the resulting MOVs generally increased with increasing dopant content.

Binary ZnO-0.25–2 mol.% V_2_O_5_ systems sintered in air at a temperature of 900 °C for 0.5–8 h usually exhibit an inhomogeneous microstructure with a fine-grained ZnO matrix but with oblong-shaped grains, indicating anisotropic grain growth influenced by the sintering time [28,170].

An effective method to achieve a uniform and homogeneous microstructure with evenly distributed ZnO grains throughout the MOV disc and well-defined GBs is by doping binary ZnO-0.5 mol.% V_2_O_5_ systems with 0.1 mol.% Nb_2_O_5_ [28]. Another approach is doping ternary ZnO-0.5 mol.% V_2_O_5_-1 mol.% Nb_2_O_5_ (ZVN) systems [44] with 0.1 mol.% In_2_O_3_, followed by sintering in air at 850–925 °C.

The fine-grained microstructure of the ZnO-V_2_O_5_-Nb_2_O_5_-In_2_O_3_ (ZVNI) systems is mainly ascribed to the spinel particle pinning mechanism [150], which is generated by the secondary spinel phases, such as Zn_3_(VO_4_)_2_ and InVO_4_, localized at the edges of the ZnO grains. The segregation at the ZnO GB regions is explained by the fact that both Nb_2_O_5_ and In_2_O_3_ are insoluble MO dopants with larger ionic radii than that of ZnO [146,147].

The Zn_3_(VO_4_)_2_ grains observed in the ZnO-0.25–2 mol.% V_2_O_5_ (ZV) systems, as well as ZV doped with 1 mol.% MnO_2_ or Co_3_O_4_ with a lower ionic radius than that of ZnO, were found to be enclosed within the ZnO grains. In contrast, the Zn_7_Sb_2_O_12_ spinel grains identified in ZnO-0.25 mol.% V_2_O_5_-2 mol.% Sb_2_O_3_ (ZVS) systems, and ZVS varistors doped with 1 mol.% MnO_2_ + 1 mol.% Co_3_O_4_, were located either individually or in clusters among the ZnO grains at TGJs [30].

The increase in the mean ZnO grain size of the ZnO-0.5–4 mol.% V_2_O_5_-based systems sintered in air by SSS (Table S1) or by TSS (Table S2) was attributed to the presence of a V_2_O_5_-rich liquid phase. This is due to the low melting temperature of V_2_O_5_ (681 °C), which acts as a sintering aid [50]. In all cases, the LPS contributed to the consolidation and densification of the sintered MOVs since the eutectic temperature of the binary ZnO-V_2_O_5_ system (~600 °C) was lower than the sintering temperature of MOVs (800–1300 °C) [125].

The secondary phases (e.g., Zn_3_(VO_4_)_2_, ZnV_2_O_4_, ErVO_4_, and Er-rich phases) formed at the GBs contributed to slower grain coarsening and generally enhanced the densification of the ZnO matrix [36] at lower sintering temperatures (800–900 °C). The grain growth was faster for the MOVs sintered over 900 °C, while the density decreased with increasing sintering temperature from 900 °C to 1300 °C due to the volatility of the V-related species [25,28,36,38,44,50,125,169,172]. In addition, a eutectic reaction occurs between ZnO and Zn_3_(VO_4_)_2_ at around 890 °C. Consequently, processing MOVs via the powder metallurgy (PM) route at temperatures equal to or greater than 900 °C involves the V-rich liquid phase of Zn_3_(VO_4_)_2_, contributing to the improved densification of MOVs through the solution and reprecipitation of ZnO [38,185].

The addition of up to 2 mol.% Er_2_O_3_ to the ZnO-V_2_O_5_ systems sintered in air at temperatures ranging from 875 °C to 950 °C resulted in the formation of Er-rich and ErVO_4_ spinel phases at the GBs and triple points, which hindered ZnO grain growth [28,169,174]. As a result, a fine-grained microstructure was achieved due to the considerable number of spinel grains that acted as ZnO grain growth inhibitors. However, the grain coarsening increased with an increase in sintering temperature, while the decrease in MOV densification was proportional to the increase in Er_2_O_3_ content. Similar findings were observed in ZnO-V_2_O_5_-based systems doped with other REOs, such as Y_2_O_3_, Sm_2_O_3_, Nd_2_O_3_, Dy_2_O_3_, Yb_2_O_3_, Gd_2_O_3_, etc. [27,44,87,173,181], sintered under comparable conditions. All REOs gradually inhibited the growth of ZnO grains during sintering and hindered the densification of MOVs with an increase in the content of REOs and RE-related secondary phases, as well as with an increase in sintering temperature.

Nahm [27,172] noticed that the effect of 0.05–0.25 mol.% Dy_2_O_3_ or Bi_2_O_3_ doping on the sintered densities of ZnO-0.5 mol.% V_2_O_5_-2 mol.% MnO_2_-0.1 mol.% Nb_2_O_5_ (ZVMN) systems sintered in air at 900 °C was different. This was evident from a decrease in density for 0.05 mol.% Dy_2_O_3_ or Bi_2_O_3_-doped MOVs compared to undoped ZVMN systems due to the volatility of V-related species, and an increase in density for 0.05–0.25 mol.% Dy_2_O_3_ or Bi_2_O_3_-doped MOVs due to a higher amount of DyVO_4_ or BiVO_4_ phase.

### 5.3. Electrical and Dielectric Properties of MOV Discs from ZnO-V_2_O_5_-Based Systems

#### 5.3.1. Electrical Properties of MOV Discs from ZnO-V_2_O_5_-Based Systems

The I–V or J–E electrical characteristics of the metallized MOV discs are typically measured below 100 mA/cm^2^ using a DC or 60-Hz AC source measure unit. However, for the characteristics above 1 A/cm^2^, an impulse current generator with a standard 8/20 µs pulse current waveform (width of 20 µs and rise time of 8 µs) is used [65].

In most studies related to ZnO-based systems, critical parameters are determined and further presented. The breakdown field (E_B_) is the electric field measured at 1 mA/cm^2^ [36,50,179], while the leakage current density (J_L_) is commonly measured at 75% or 80% of the E_B_, which corresponds to 0.75 × E_B_ [35,37,44,169] or 0.8 × E_B_ [50,160,162,183], respectively. Alternatively, J_L_ can be determined at the nominal voltage [186].

The nonlinear coefficient (α), denoted also as α_1_ in the low-current region, is calculated from one of the equations shown below [6,16,35,37,44,50,160,162,169,183,187]:

(15) $$\alpha =\left[\log_{10}\left(I_{2}/I_{1}\right)\right]/\left[\log_{10}\left(V_{2}/V_{1}\right)\right]=\left(\mathrm{lg}I_{2}-\mathrm{lg}I_{1}\right)/\left(\mathrm{lg}V_{2}-\mathrm{lg}V_{1}\right)$$

(16) $$\alpha =\left[\log_{10}\left(J_{2}/J_{1}\right)\right]/\left[\log_{10}\left(E_{2}/E_{1}\right)\right]=\left(\mathrm{lg}J_{2}-\mathrm{lg}J_{1}\right)/\left(\mathrm{lg}E_{2}-\mathrm{lg}E_{1}\right)$$

where *V_1_* and *V_2_* are the voltages corresponding to the currents *I_1_* and *I_2_*, while *E_1_* and *E_2_* are the applied electric fields corresponding to the current densities *J_1_* and *J_2_* (typical values: *J_1_* = 1 mA/cm^2^, *J_2_* = 10 mA/cm^2^ [16,44,50], or *J_1_* = 0.1 mA/cm^2^, *J_2_* = 1 mA/cm^2^ [16,37,187]).

Researchers typically use a GB defect model to determine the GB parameters, which attributes electric conduction in the linear (ohmic) region to thermionic emission in DSBs, thus influencing the basic conduction mechanism [84]. Moreover, the interface charge in the GBs is dependent on the electric field, and electron–hole (e^−^–h^+^) creation and recombination lead to electrical breakdown (EBD) [84]. When the electric field is over 40 MV/m and the doping density (N_d_) in ZnO is higher than 10^17^ cm^−3^, e^−^–h^+^ pairs can be produced through hot-electron impact ionization in the depletion regions at the GBs [84].

The critical field strength (40 MV/m) and N_d_ (10^17^ cm^−3^) can be achieved if the sum of Φ_B_ and e·U exceeds 4.5 eV, where Φ_B_ is the Schottky barrier height in the absence of an external electric field across the MOV, e is the elementary charge (proton charge), and U is the applied voltage (i.e., the electric potential difference) between two ZnO grains. Hole creation in MOVs results in an EBD at the GBs if the applied voltage exceeds approximately 3.5 V [14,84,188].

Equation (17) defines the relationship between the current density (J) and Schottky barrier height (Φ_B_) [25,38]:

(17) $$J=A^{*}\cdot T^{2}\cdot \exp\left(\frac{\beta \cdot E^{1/2}-\Phi_{B}}{k_{B}\cdot T}\right)$$

where A* is the Richardson constant for thermionic emission in DSBs (A* = 30 A/cm^2^K^2^ for ZnO), *T* is the absolute temperature (300 K), *E* is the electric field, *k_B_* is the Boltzmann constant (*k_B_* = 8.617 × 10^−5^ eV/K), and *β* is a constant deduced from Equation (18) [16]:

(18) $$\beta ={\left(\frac{1}{\gamma \cdot \omega }\;\cdot \frac{2e^{3}}{4\pi \cdot \varepsilon_{0}\cdot \varepsilon_{r}}\right)}^{1/2}$$

where *γ* is the number of grains per unit length, *ω* is the barrier width (depletion layer width) at either side of the GBs, e is the elementary charge (e = 1.602 × 10^−19^ C), ε_0_ is the vacuum dielectric constant (ε_0_ = 8.85 × 10^−14^ F/cm), and ε_r_ is the relative dielectric constant (ε_r_ = 8.5 for ZnO) [188]. The deduction of the Φ_B_ and β is described elsewhere [16].

The barrier width (ω) is determined from Equation (19) [63]:

(19) $$\omega =\frac{1}{\beta^{2}\cdot \gamma }\cdot K^{\prime }=\frac{1}{\beta^{2}\cdot \gamma }\cdot \frac{e^{3}}{2\pi \varepsilon }$$

where *β* is a constant obtained from the slope of the ln*J* versus *E*^1/2^, considering the data from the ohmic region; γ is the number of grains per unit length; *K′* = e^3^/(2πε) is a constant dependent on the elementary charge (e) and the dielectric constant (ε).

The donor density (N_d_) and the interface state density (N_s_) at the GBs are expressed in Equations (20) and (21), where *K*″ is a constant (*K*″ = e/(π *K*′) = 2ε/e^2^) [63]:

(20) $$N_{d}=\frac{\Phi_{B}}{\omega^{2}}\cdot K^{\prime \prime }=\frac{\Phi_{B}}{\omega^{2}}\cdot \frac{2\varepsilon }{e^{2}}$$

(21) $$N_{s}=N_{d}\cdot \omega$$

**Figure 15:**
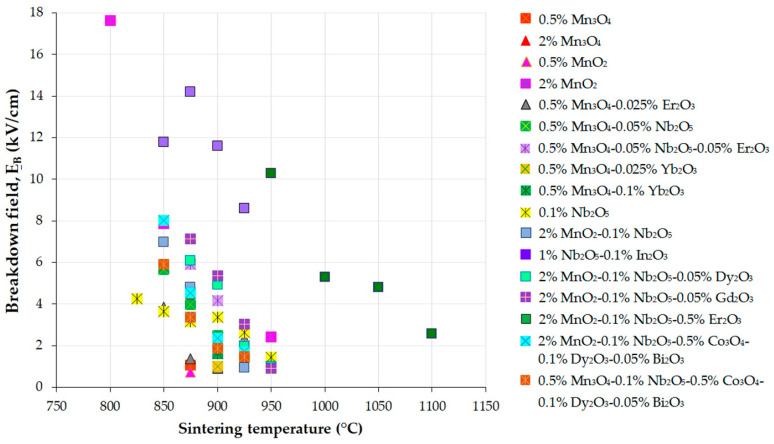
Variation in the breakdown field (E_B_) with the sintering temperature of MOVs from the ZnO-0.5 mol.% V_2_O_5_-based systems doped with various MOs (mol.%). The data plotted in the figure were collected from references [27,36,44,50,60,144,161,173,174,179,180,181,182,183].

**Figure 16:**
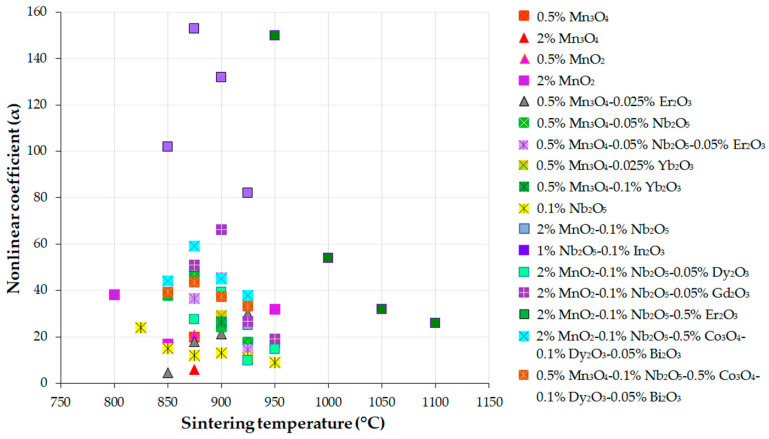
Variation in the nonlinear coefficient (α) in the low-current region with the sintering temperature of MOVs from the ZnO-0.5 mol.% V_2_O_5_-based systems doped with various MOs (mol.%). The data plotted in the figure were collected from references [27,36,44,50,60,144,161,173,174,179,180,181,182,183].

**Figure 17:**
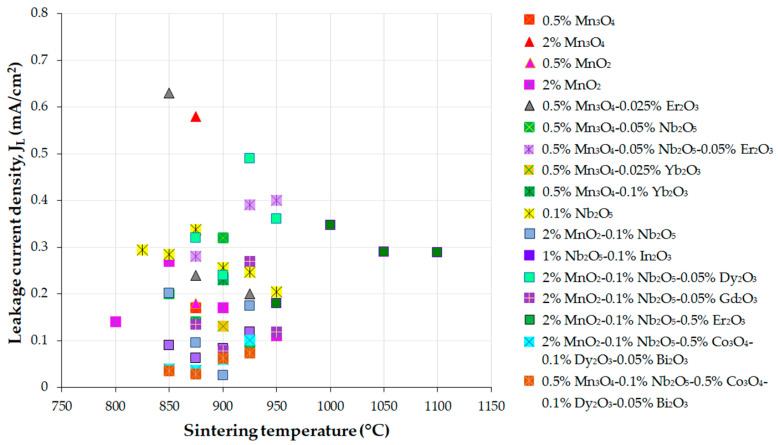
Variation in the leakage current density (J_L_) with the sintering temperature of MOVs from the ZnO-0.5 mol.% V_2_O_5_-based systems doped with various MOs (mol.%). The data plotted in the figure were collected from references [27,36,44,50,60,144,161,173,174,179,180,181,182,183].

Figure 15, Figure 16 and Figure 17 present the variations in the breakdown field (E_B_), nonlinear coefficient (α) in the low-current region, and leakage current density (J_L_) with the sintering temperature of MOVs from ZnO-0.5 mol.% V_2_O_5_-based systems.

The decrease in E_B_ with an increase in sintering temperature (Figure 15) is attributed to the reduction in the number of GBs (*N_gb_*) across the thickness (*t*) of the MOV disc due to the increase in the mean ZnO grain size (*d*) and the corresponding decrease in the breakdown voltage drop per GB (*V_gb_*), as shown in Equations (22) and (23), where *V_B_* is the total voltage drop [36,38,44,62,124,161,188]. In contrast, the increase in E_B_ is caused by the decrease in the mean ZnO grain size, leading to an increase in the number of GBs [184].

(22) $$V_{B}=N_{gb}\cdot V_{gb}=\frac{t}{d}\cdot V_{gb}$$

(23) $$E_{B}=\frac{V_{B}}{t}=\frac{V_{gb}}{d}$$

The nonlinearity (α) of the MOVs from the ZnO-based systems is greatly dependent on the electrical potential barrier height (Φ_B_) at the GBs. This is expressed as the absolute value of the difference between the Fermi energy levels in the GB (E_FB_) and the ZnO grains (E_FG_), where E_FB_ < E_FG_, as shown in Equation (24) [8]:

(24) $$\Phi_{B}=\left|E_{FB}-E_{FG}\right|=E_{FG}-E_{FB}$$

During the sintering process of ZnO-V_2_O_5_- and ZnO-Bi_2_O_3_-based systems, point defects occur at the GBs, which relocate the *E_FB_* to a higher position in the band gap energy (*E_g_*). This shift in *E_FB_* creates DSBs (depletion zones at either side of the GBs), resulting in the formation of potential barriers [3,8]. Furthermore, changes in the sintering temperature can modify the interface state density (N_s_) at the GBs, causing the defect ions (native or additive donors) to move toward the GBs and generate more active GBs [180].

Doping ZnO grains with small amounts of metal oxides, such as MnO_2_, Mn_2_O_3_, In_2_O_3_, Sb_2_O_3_, Cr_2_O_3_, and others, in ZnO-V_2_O_5_- and ZnO-Bi_2_O_3_-based systems, can increase the Fermi energy levels in the ZnO grains (*E_FG_*), causing a shift in the band gap energy (E_g_). This leads to an improvement in Φ_B_, resulting in better nonlinearity (higher α) and a lower leakage current (I_L_) or leakage current density (J_L_) compared to pure n-type ZnO semiconductors with a direct band gap energy of ~3.37–3.4 eV at RT [5,138,152], which exhibit very low nonlinearity and almost equal Fermi energy levels in the GBs and ZnO grains [8].

The redshift in the band gap energy towards lower energies can indicate the incorporation of different metal ions from the MO additives into the Zn^2+^ lattice during the LPS process [189]. Adjusting the band gap energy (*E_g_*) of doped wurtzite ZnO-based systems in the range of 3–4.5 eV can improve their varistor properties [129].

The enhancement in nonlinearity (α) with increasing sintering temperature (as shown in Figure 16) was attributed to the variation in the Schottky barrier height, which changes with the electronic states at the GBs during sintering when defect ions are promoted near the GBs [179]. Therefore, the decrease in the potential barrier height (Φ_B_) at the GBs led to the reduction in the α values as the sintering temperature varied [180].

Table 3 summarizes the electrical properties (E_B_, α, and J_L_) of selected ZnO-0.5 mol.% V_2_O_5_-based systems before and after conducting DC-accelerated aging stress. The values of ΔE_B_/E_B_ (%), Δα/α (%), and ΔJ_L_/J_L_ (%) were determined using Equations (25)–(27), while the degradation rate coefficient (K_T_) was determined using Equation (28) [27]:

ΔE_B_/E_B_ = [(E_Bstressed_ − E_Binitial_)/(E_Binitial_] × 100 (25)

Δα/α = [(α_stressed_ − α_initial_)/α_initial_] × 100 (26)

ΔJ_L_/J_L_ = [(J_Lstressed_ − J_Linitial_)/J_Linitial_] × 100 (27)

K_T_ = (I_L_ − I_L0_)/t^1/2^ (28)

where I_L_ is the leakage current corresponding to the stress time t, and I_L0_ is I_L_ at t = 0. Furthermore, the stability of MOVs is higher as the K_T_ and J_L_ values are lower [144].

Manganese (Mn) doping at levels of 0.5–2 mol.% had a significant impact on the electrical properties of ZnO-0.5 mol.% V_2_O_5_ (ZV) systems sintered in air at 900 °C for 3 h [144]. MOVs doped with 2 mol.% MnO_2_ (ZVM systems) or 0.5 mol.% Mn_3_O_4_ (ZVM* systems) exhibited superior varistor properties. The ZVM-2 mol.% MnO_2_ systems produced similar electrical breakdown (E_B_) values (~1 kV/cm), better nonlinearity in the initial and stressed states (α = 20–27), and low leakage current density (J_L_) values (0.042–0.21 mA/cm^2^). These systems also demonstrated higher stability against DC aging stress, with the lowest degradation rate coefficient (K_T_ = 3.8 µA h^−1/2^) compared to the ZVM*-0.5 mol.% Mn_3_O_4_ systems (α = 11–20, J_L_ = 0.17–0.48 mA/cm^2^, K_T_ = 12 µA h^−1/2^). In contrast, the ZVM-0.5 mol.% MnO_2_ systems [144] exhibited the highest electrical degradation (K_T_ = 35.3 µA h^−1/2^), while the ZVM*-2 mol.% Mn_3_O_4_ systems showed high E_B_ values (~2.6–4.4 kV/cm), low nonlinearity (α = 3–6), high J_L_ values (0.58–0.74 mA/cm^2^), and thermal runaway during the stress time (Table 3). The poor electrical behavior in this instance was caused by the high leakage current and poor GBs, resulting in low electrical potential barriers (DSBs).

According to a study presented in [144], the capacitance–voltage (C–V) characteristics, specifically the donor density (N_d_) and barrier height (Φ_B_) determined by Equations (29) and (30) [180,183], were found to be dependent on the content and valences of Mn (Mn^4+^ in MnO_2_, and Mn^2.66+^ in Mn_3_O_4_). The Φ_B_ of the Mn_3_O_4_-doped ZVM* systems decreased from 2.66 eV to 1.32 eV as the dopant content increased from 0.5 mol.% to 2 mol.%, while the Φ_B_ of the MnO_2_-doped ZVM systems increased from 1.47 eV to 1.99 eV. On the other hand, the N_d_ variation showed the opposite tendency to that of the Φ_B_. This behavior was influenced by the change in the partial pressure of oxygen (O_2_) in the doped MOVs. The higher Φ_B_ led to better nonlinearity, indicated by higher α values, which is associated with the conduction mechanism [144].

(29) $${\left[1/C_{b}-1/\left(2C_{b0}\right)\right]}^{2}=2\left(\Phi_{B}+V_{gb}\right)/\left(q\cdot \varepsilon \cdot N_{d}\right)$$

where *C_b_* denotes the capacitance per unit area of a GB, *C_b0_* is the *C_b_* corresponding to the *V_gb_* = 0 V, *V_gb_* is the applied voltage per GB, q is the proton charge or elementary charge (*q* = *e* = 1.602 × 10^−19^ C), and *ε* is the permittivity of ZnO (*ε* = 8.5 × *ε*_0_). The Φ_B_ and N_d_ values are computed via the intercept, and, respectively, the slope of the line on the V_gb_ axis from the ${\left[1/C_{b}-1/\left(2C_{b0}\right)\right]}^{2}$ versus *V_gb_* plot. The *Φ_B_* can also be expressed as follows [49]:

(30) $$\Phi_{B}=\left(e^{2}\cdot N_{s}^{2}\right)/(2N_{d}\cdot \varepsilon )=\left(e^{2}\cdot N_{s}^{2}\right)/(2N_{d}\cdot \varepsilon_{0}\cdot \varepsilon_{r})$$

Nahm [36] investigated the effect of the sintering temperature (800–950 °C) on the electrical properties and aging characteristics of ternary ZnO-0.5 mol.% V_2_O_5_-2 mol.% MnO_2_ (ZVM) systems. The MOVs sintered at 800–900 °C showed a decrease in E_B_ from 17.64 kV/cm to 0.99 kV/cm in the initial state, due to an increase in mean ZnO grain size from 2.1 μm to 5.2 μm, and a decrease in V_gb_ from 3.7 V/GB to 0.5 V/GB with increasing sintering temperature. Despite being dense (RD of 96.2–96.7%) and having good nonlinear properties (α = 17–38, J_L_ = 0.11–0.27 mA/cm^2^) in the initial state, the MOVs sintered at lower temperatures (800–850 °C) displayed shallow stability after conducting DC-accelerated aging stress and thermal runaway during the stress duration. However, the stress time increase progressively degraded the E–J characteristics. This study attributed the poor behavior to the uneven distribution and content increase in the Zn_3_(VO_4_)_2_ phase, along with a decrease in the number of conduction paths at the GBs.

The ZVM systems sintered at 900–950 °C [36] exhibited good varistor properties both in the initial and stressed states (E_B_ ≅ 1–2.4 kV/cm, α = 20.1–32) and did not experience thermal runaway during the DC aging stress duration. The MOVs sintered at 900 °C exhibited a low degradation rate coefficient (K_T_) of 3.8 µA h^−1/2^ and demonstrated the best electrical stability, with ΔE_B_/E_B_ of 0.6% and Δα/α of -26.1% (Table 3). In contrast, the MOVs sintered at 950 °C exhibited a higher K_T_ of 9 µA h^−1/2^ (Table 3). The good electrical behavior of the MOVs was attributed to the maintenance of a consistent ZnO grain size and depletion layer width (ω) after DC aging stress.

Park et al. [50] investigated the effect of the sintering temperature on the electrical properties of 0.05 mol.% Nb_2_O_5_-doped ZVM* (referred to as ZVM*N) systems. The ceramics were sintered at temperatures ranging from 875 °C to 950 °C for 3 h before and after DC aging stress. All ceramic materials exhibited good varistor properties, with the breakdown voltage (E_B_) ranging from 1.4 kV/cm to 5.7 kV/cm, nonlinearity (α) ranging from 17.8 to 47, and leakage current density (J_L_) ranging from 0.09 mA/cm^2^ to 0.32 mA/cm^2^. As the sintering temperature increased from 875 °C to 950 °C, E_B_ decreased, and the MOVs sintered at 900 °C achieved the highest α. However, the MOVs sintered at 950 °C showed the lowest J_L_ and almost the same nonlinearity in the low-current region (α_initial_ = 17.8, and α_stressed_ = 17.7), as well as the highest stability (ΔE_B_/E_B_ = 0.4%, Δα = −0.6%, and ΔJ_L_/J_L_ = 22.2%) after conducting DC-accelerated aging stress (Table 3). The aging mechanism of the ZVM*N varistors was attributed to the variation in the Schottky barrier combined with alternative cycles between Joule heating and the leakage current [50].

The ZVN [28], ZVMN [180], ZVMNG [173], ZVMND [181], and ZVNI [44] systems (mol.%) containing 0.5 mol.% V_2_O_5_, 0.1 mol.% Nb_2_O_5_, 2 mol.% MnO_2_, 0.05 mol.% Gd_2_O_3_, or Dy_2_O_3_ and 0.1 mol.% In_2_O_3_ sintered in air at temperatures between 800 °C and 975 °C for 1–3 h yielded an E_B_ of approximately 0.9–7.2 kV/cm for most MOV systems and higher E_B_ (8.6–14.2 kV/cm) for the ZVNI systems. In general, the trend for the E_B_ was to decrease with the increase in sintering temperature, but the MOVs sintered at 850–900 °C achieved the highest E_B_.

The ZVNI systems doped with 0.1 mol.% In_2_O_3_ and sintered in air at 850–925 °C for 1 h exhibited superior nonlinearity (α = 76–160) and lower values of J_L_ (0.06–0.113 mA/cm^2^) compared to those of ZVN (α = 9–24, J_L_ = 0.204–0.294 mA/cm^2^), ZVMN (α = 25–50, J_L_ = 0.026–0.201 mA/cm^2^), ZVMNG (α = 19–66.1, J_L_ = 0.077–0.27 mA/cm^2^), and ZVMND systems (α = 9.9–39.2, J_L_ = 0.24–0.49 mA/cm^2^). Generally, the MOVs sintered at 900 °C achieved the highest nonlinearity (α) in the low-current region. Despite the promising results, the stability of the developed MOVs, including the ZVN, ZVNI, ZVMNG, and ZVMND systems [28,44,173,181], against DC aging stress or pulse surge current aging stress is uncertain, but some authors [44] plan to address this issue in further studies.

According to [180], ZVMN systems sintered at 925 °C showed the highest electrical stability without thermal runaway (ΔE_B_/E_B_ = 1.5%, Δα/α = 13.2%, and ΔJ_L_/J_L_ = 112.4%) and with very low degradation (K_T_ = 0.38 µA h^−1/2^) after DC-accelerated aging stress (Table 3). Furthermore, an enhancement in nonlinearity and very low leakage current density were observed both in the initial state (α = 38, J_L_ ≅ 0.026 mA/cm^2^) and in the stressed state (α = 43, J_L_ ≅ 0.055 mA/cm^2^). Good electrical stability without thermal runaway during the stress time was demonstrated by the MOVs sintered at 900 °C, which exhibited the best nonlinearity in the initial state (α = 50) and the maximum barrier height (Φ_B_ of 1.07 eV). However, the nonlinear coefficient decreased by 60% after DC aging stress. MOVs subjected to sintering at lower temperatures (875–900 °C) or higher temperatures (950 °C) resulted in failure or high degradation (K_T_ of 20.8–27.6 µA h^−1/2^). The leakage current density of the MOVs increased the most (0.51–0.72 mA/cm^2^) in the stressed state for those sintered at 875 °C and 950 °C. The GB parameters, including the donor concentration (N_d_), showed an increasing trend in the range of (3.33–7.64) × 10^17^ cm^−3^ with increasing sintering temperature due to ZnO dissociation.

The study of Nahm [27] revealed the effect of 0.025–0.25% Yb_2_O_3_ doping on the stability of ZVM* systems sintered at 900 °C for 3 h against DC aging. The Yb_2_O_3_-doped ZVM* systems exhibited an initial breakdown voltage (E_B_) of 1–3.8 kV/cm, nonlinearity (α) of 5.7–29.2, and a leakage current density (J_L_) of 0.13–0.60 mA/cm^2^ in the initial state. The E_B_ and J_L_ increased with the Yb_2_O_3_ content increase, as the doping led to a decrease in d_ZnO_ (Table S1), resulting in an increase in the N_gb_. MOVs doped with 0.025 mol.% Yb_2_O_3_ attained the highest nonlinearity (α = 29.2), but the most increased stability against DC aging stress (ΔE_B_/E_B_ = 0.7%, Δα = −4.2%, and K_T_ = 5.5 µA h^−1/2^) was exhibited by those doped with 0.1 mol.% Yb_2_O_3_ (Table 3). The lack of the YbVO_4_ secondary phase in the undoped MOVs or the high content of YbVO_4_ in the case of 0.25 mol.% Yb_2_O_3_-doped MOVs worsened their stability against DC aging stress (K_T_ = −19.4 µA h^−1/2^), as confirmed by this study. It was observed that MOVs with a low nonlinear coefficient (α < 10) typically exhibited a negative K_T_ value.

The ZVM-2 mol.% MnO_2_ and ZVM*-0.5 mol.% Mn_3_O_4_ systems doped with 0.1% Nb_2_O_5_-0.5% Co_3_O_4_-0.1% Dy_2_O_3_-0.05% Bi_2_O_3_ (mol.%) [183] and sintered at 850–900 °C for 3 h showed excellent varistor properties in the initial and DC stressed states (Table 3). The ZVMNCDB systems with 2 mol.% MnO_2_ exhibited better electrical properties (E_B_ ≅ 2.4–8 kV/cm, α = 21–59, and J_L_ = 0.036–0.239 mA/cm^2^) compared to the ZVM*NCDB systems with 0.5 mol.% Mn_3_O_4_ (E_B_ ≅ 1.6–5.9 kV/cm, α = 13.2–43.6, and J_L_ = 0.028–0.402 mA/cm^2^). The ZVMNCDB systems sintered at 900 °C achieved the highest stability (ΔE_B_/E_B_ = 0.8%, Δα/α = 0%). In contrast, the ZVM*NCDB systems sintered at 850 °C were more stable (ΔE_B_/E_B_ = −2%, Δα/α = −23.3%) than the analogous MOVs sintered at higher temperatures.

El-Rabaie et al. [38] investigated the electrical behavior of ZVM*-0.5 mol.% Mn_3_O_4_ systems sintered in air at various temperatures (825–950 °C) for 3 h. The MOVs sintered at 875 °C showed the best E–J characteristics (α = 19.81, J_L_ = 0.261 mA/cm^2^). An E_B_ decrease from 2.11 kV/cm to 1.43 kV/cm was observed due to an increase in mean ZnO grain size from 20.55 μm to 24.23 μm, as well as a decrease in V_gb_ from 4.34 V/GB to 3.18 V/GB with the increase in sintering temperature from 825 °C to 950 °C. The Schottky barrier height (Φ_B_) in the GBs (0.894–0.928 eV) and the barrier width (ω) at either side of the GBs (30.53–55.28 nm) varied with the change in sintering temperature, similarly to the α variation.

The ZVMN systems (mol.%) doped with 2% MnO_2_ and 0.1% Nb_2_O_5_, prepared using the powder metallurgy (PM) route at a lower pressing pressure (100 MPa) and sintering temperatures of 875–950 °C for 3 h [27,162,180], exhibited better E–J characteristics (E_B_ ≅ 0.9–7 kV/cm, α = 25–50, J_L_ = 0.026–0.3 mA/cm^2^) than comparable MOV ceramics that were pressed at a higher pressure (500 MPa) and sintered at temperatures of 900–1300 °C for 0.5–8 h [62,174]. The latter exhibited lower E_B_ values (0.8–3.6 kV/cm) and α values (8–17.5).

The undoped and 0.05 mol.% REO (Y_2_O_3_, Nd_2_O_3_, or Sm_2_O_3_) doped ZnO-0.5 mol.% V_2_O_5_-2 mol.% MnCO_3_ systems were pressed at low pressure (100 MPa) and sintered in air at 930 °C for 3 h [25]. These MOV systems also exhibited good varistor properties, with E_B_ ranging from 2.8 kV/cm to 3 kV/cm, α from 25.9 to 36.5, and J_L_ from 0.039 mA/cm^2^ to 0.188 mA/cm^2^. Among the REO-doped MOVs, Sm_2_O_3_-doped ones had the lowest J_L_, whereas Y_2_O_3_-doped MOVs showed the highest nonlinearity, with α at 36.5.

In a study carried out by Zhao et al. [160], the effect of 0.1 mol.% Ce-La doping on the electrical behavior of MOVs (mol.%) from the ZnO-0.5% V_2_O_5_-2% MnCO_3_-0.1% Nb_2_O_5_ (ZnVMnNbO) systems was investigated. The samples were obtained by pressing at 100 MPa and sintering at temperatures ranging from 850 °C to 925 °C for 3 h. The results showed that the optimum sintering temperature was 875 °C, at which the Ce-La-doped MOVs exhibited better E–J characteristics (E_B_ = 5.54 kV/cm, α = 48.4, J_L_ = 0.12 mA/cm^2^) compared to those sintered at 850 °C or 925 °C. The 0.1 mol.% Ce-La-doped MOVs had lower nonlinearity (α of 48.4) than MOV systems doped with 0.05 mol.% REs (Er, Gd, or Tb) (α of 63–67.9), but higher than that of several commercial ZnO-based MOVs.

Many studies have reported on the variation in electrical properties with a change in dopant content for ZnO-0.5% V_2_O_5_-2% MnO_2_-0.1% Nb_2_O_5_ (ZVMN) systems (mol.%) doped with 0.05–0.1 mol.% Dy_2_O_3_ [24], 0.025–0.1 mol.% Tb_4_O_7_ [162], or 0.025–0.25 mol.% Bi_2_O_3_ [172], which were pressed at 100 MPa and sintered at 900 °C for 3 h. All the doped MOVs yielded good electrical properties (E_B_ ≅ 2.2–5.1 kV/cm, α = 31–65.5, J_L_ = 0.02–0.25 mA/cm^2^). Generally, the values of E_B_ and α decreased, and J_L_ increased with the increase in dopant content. The Bi_2_O_3_-doped MOVs exhibited the lowest J_L_ (0.02–0.055 mA/cm^2^), followed by Tb_4_O_7_-doped MOVs (J_L_ = 0.072–0.172 mA/cm^2^). The 0.025 mol.% Tb_4_O_7_-doped MOVs achieved the best nonlinearity (α = 65.5), followed by 0.025 mol.% Bi_2_O_3_-doped MOVs (α = 60) and 0.1 mol.% Dy_2_O_3_-doped MOVs (α = 53.3). The 0.025 mol.% Bi_2_O_3_-doped MOVs reached the highest barrier height (1.12 eV), suggesting superior varistor properties [172] compared to Dy_2_O_3_- [24] or Tb_4_O_7_-doped MOVs [162].

**Figure 18:**
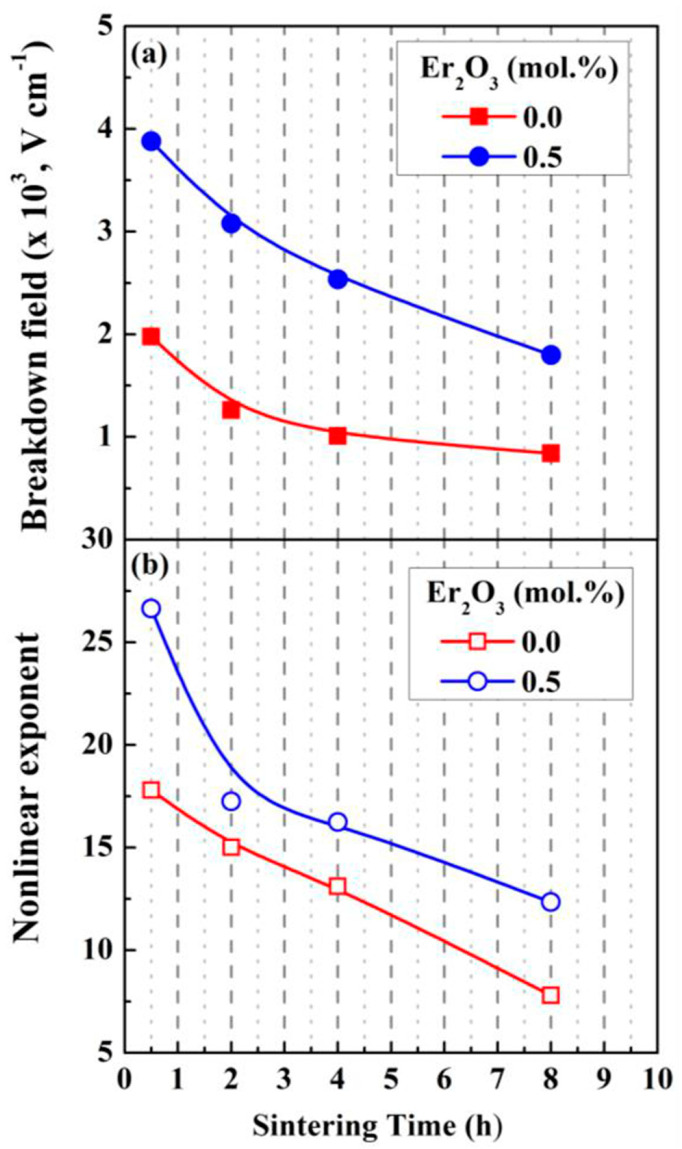
Variation in (**a**) the breakdown field (E_B_) and (**b**) the nonlinear coefficient (α) with the sintering time of the ZVMN systems with 0–0.5 mol.% Er_2_O_3_ sintered at 1100 °C for 0.5–8 h. Reprinted from reference [62], published under an open access Creative Commons Attribution (CC BY 3.0) license (https://creativecommons.org/licenses/by/3.0/ (accessed on 14 October 2022)) in IOP Conf. Ser. Mater. Sci. Eng. by IOP Publishing Ltd.

**Figure 19:**
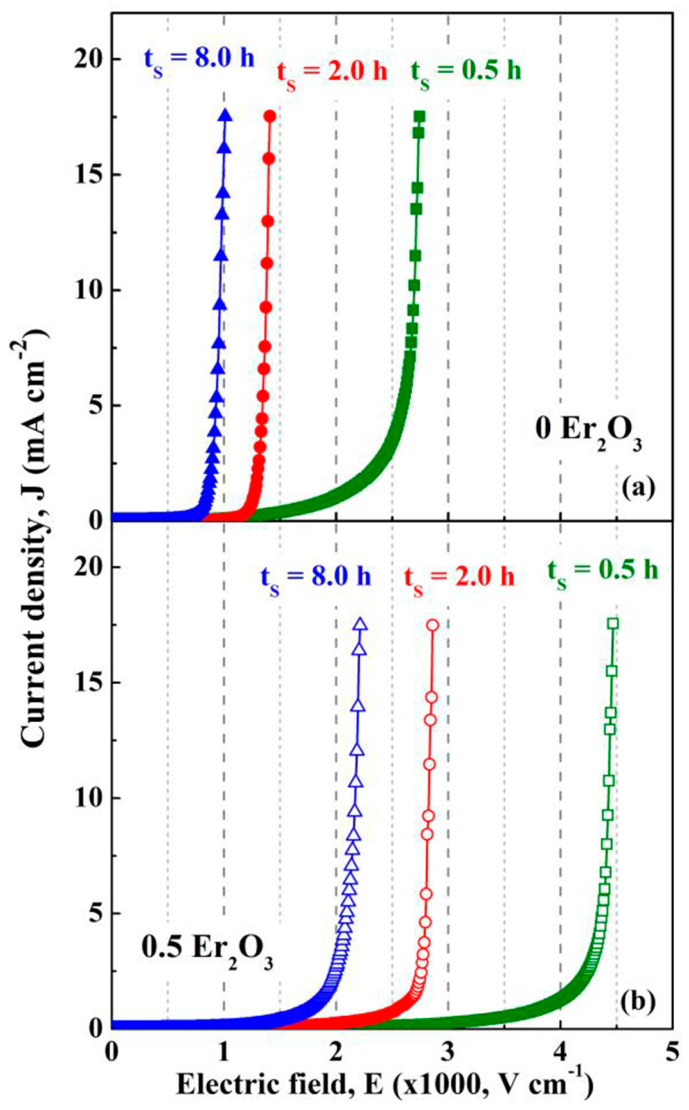
Variation in the current density (J) with the electric field (E) of (**a**) undoped and (**b**) 0.5 mol.% Er_2_O_3_-doped ZVMN systems sintered at 1100 °C for 0.5–8 h. Reprinted from reference [62], published under an open access Creative Commons Attribution (CC BY 3.0) license (https://creativecommons.org/licenses/by/3.0/ (accessed on 14 October 2022)) in IOP Conf. Ser. Mater. Sci. Eng. by IOP Publishing Ltd.

Roy et al. [62] investigated the properties of ZVMN systems doped with 0.5 mol.% Er_2_O_3_ (referred to as ZVMNE systems), which were pressed at 500 MPa and sintered at 1100 °C for dwell times ranging from 0.5 h to 8 h. The ZVMNE varistors showed a decrease in E_B_ from 3.9 kV/cm to 1.8 kV/cm, and a decrease in α from 27 to 12.5 with increasing dwell time from 0.5 h to 8 h (Figure 18 and Figure 19). Similarly, in another study using the same ZVMNE formulation as reference [62], but with MOV pressing at 500 MPa and sintering at 1050 °C for 1 h [169], good varistor characteristics were obtained (E_B_ = 4.8 ± 0.1 kV/cm, α = 32 ± 2, J_L_ = 0.29 ± 0.03 mA/cm^2^). The Er_2_O_3_-doped MOVs demonstrated superior electrical properties compared to those of undoped MOVs, as expected.

Several authors [63,163] have studied the effect of 0.05–0.1 mol.% Er_2_O_3_ doping on ZVMN systems that were pressed at lower pressure (100 MPa) and sintered at a temperature of 875 °C for 3 h. These studies showed that Er_2_O_3_-doped ZVMN systems yielded superior E–J characteristics (E_B_ ≅ 7–7.4 kV/cm, α = 50–55, J_L_ = 0.094–0.128 mA/cm^2^) compared to undoped ZVMN systems (E_B_ ≅ 7 kV/cm, α = 44, J_L_ = 0.201 mA/cm^2^). The values of E_B_, α, and J_L_ increased with the increase in Er_2_O_3_ content from 0.05 mol.% to 0.1 mol.%.

Roy et al. [174] investigated the electrical behavior of MOVs (mol.%) in 0.5–1 mol.% Er_2_O_3_-doped ZVMN systems obtained at a higher pressing pressure (P_p_ of 500 MPa) and sintered in air at temperatures ranging from 1100 °C to 1300 °C for 1 h. This study revealed a significant decrease in both the E_B_ parameter, from ~2.6 kV/cm to 0.5 kV/cm, and the α parameter, from 26 (high nonlinearity) to 3 (low nonlinearity), with an increase in sintering temperature from 1100 °C to 1300 °C, as well as an increase in Er_2_O_3_ content from 0.5 mol.% to 1 mol.%.

A few studies [169,174] have investigated the influence of the sintering temperature on the electrical characteristics of 0.5 mol.% Er_2_O_3_-doped ZVMN (ZVMNE) systems that were pressed at 500 MPa and sintered at temperatures ranging from 950 °C to 1100 °C for 1 h. The results showed that the Er_2_O_3_-doped ZVMN systems exhibited a decrease in E_B_ from 10.3 kV/cm to 2.5 kV/cm and a significant decrease in α from 150 to 26 with an increase in sintering temperature. The varistors sintered at 950 °C exhibited the lowest J_L_ (0.18 mA/cm^2^), while the varistors sintered at 1000 °C had the highest J_L_ (0.347 mA/cm^2^).

**Figure 20:**
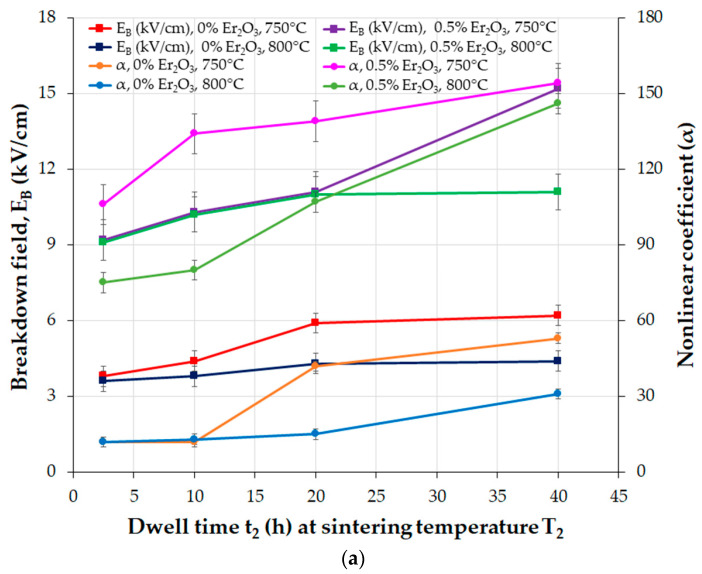

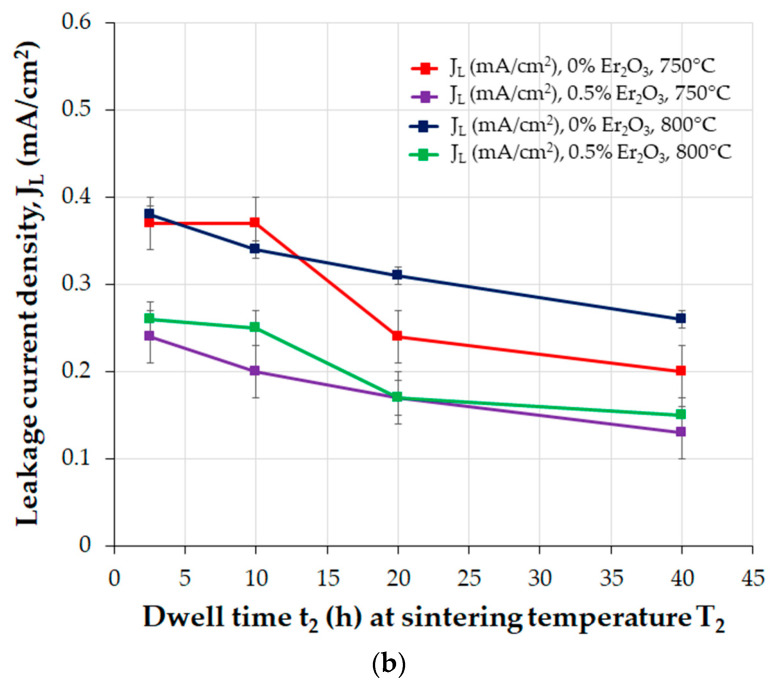
Variation in (**a**) the breakdown field (E_B_) and the nonlinear coefficient (α) and (**b**) the leakage current density (J_L_) of MOVs (mol.%) from the ZnO-0.5% V_2_O_5_-2% MnO_2_-0.1% Nb_2_O_5_ (ZVMN) systems with 0–0.5 mol.% Er_2_O_3_ prepared by a TSS process (T_1_ = 1050 °C, t_1_ = 1/6 h, and T_2_ = 750 °C or 800 °C, t_2_ = 2.5–40 h). The data plotted in the figure were collected from reference [63].

Roy et al. [63] developed MOVs (mol.%) from ZnO-0.5% V_2_O_5_-2% MnO_2_-0.1% Nb_2_O_5_ (ZVMN) systems with 0–0.5 mol.% Er_2_O_3_ via the powder metallurgy (PM) route. The samples were pressed at 500 MPa and subjected to two-stage sintering (TSS) at T_1_ = 1050 °C for t_1_ = 1/6 h and T_2_ = 750 °C or 800 °C for t_2_ = 2.5–40 h. This study showed that the Er_2_O_3_-doped MOVs (ZVMNE systems) exhibited improved electrical properties (E_B_ ≅ 8.4–16.3 kV/cm, α = 72–172, and J_L_ = 0.11–0.29 mA/cm^2^) compared to those of the undoped ZVMN systems (E_B_ ≅ 3.4–6.6 kV/cm, α = 10–56, and J_L_ = 0.17–0.39 mA/cm^2^), as shown in Figure 20. The TSS process resulted in a synergistic effect that hindered the ZnO grain growth and enhanced the electrical properties of the MOVs [63], compared to the MOVs prepared by single-stage sintering at 1050 °C for 1 h [169,174].

In Figure 20, it can be observed that the ZVMNE systems sintered by a TSS process at T_1_ = 1050 °C for t_1_ = 1/6 h and T_2_ = 750 °C for t_2_ = 40 h exhibited superior varistor properties (E_B_ = 15.2 ± 1.1 kV/cm, α = 154 ± 18, J_L_ = 0.13 ± 0.02 mA/cm^2^) [63], along with a fine-grained microstructure having a mean ZnO grain size of 1.7 μm and high relative density (RD of 97.5%). Although these sintering conditions were deemed optimal, using a prolonged dwell time at T_2_ of 40 h is not energy-efficient for industrial applications of MOVs.

**Figure 21:**
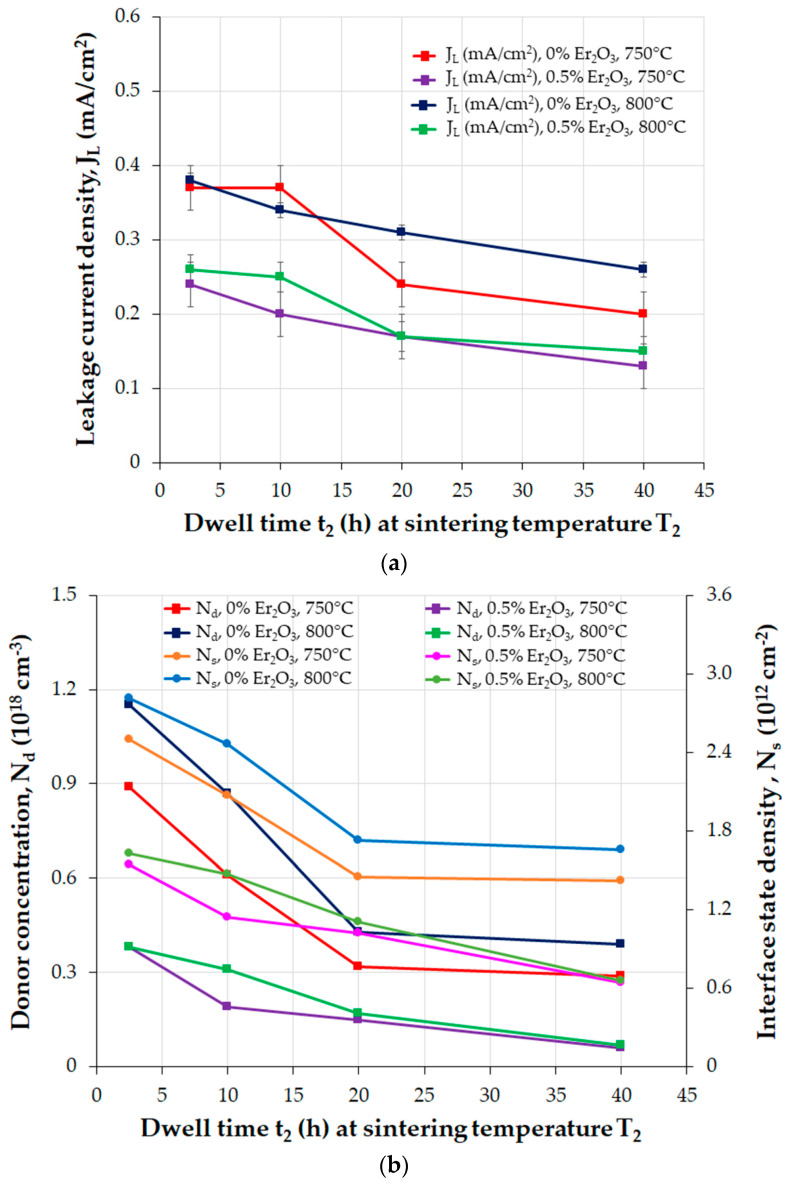
Variation in (**a**) the barrier height (Φ_B_) and barrier width (ω) and (**b**) donor concentration (N_d_) and interface state density (N_s_) of MOVs (mol.%) from the ZnO-0.5% V_2_O_5_-2% MnO_2_-0.1% Nb_2_O_5_ (ZVMN) systems with 0–0.5% Er_2_O_3_ prepared by a TSS process (T_1_ = 1050 °C, t_1_ = 1/6 h, and T_2_ = 750 °C or 800 °C, t_2_ = 2.5–40 h). The data plotted in the figure were collected from reference [63].

The nonlinearity of MOVs was influenced by the Schottky barrier height (Φ_B_) at the grain boundaries (GBs), which increased with increasing the sintering time t_2_ and the addition of Er_2_O_3_ to the ZVMN varistors. However, Φ_B_ slightly decreased with increasing T_2_ from 750 °C to 800 °C. The Φ_B_ varied within a narrow range (0.72–0.77 eV) and was dependent on the donor concentration (N_d_), the surface state density (N_s_), and the barrier width (ω) at either side of the GBs. Undoped MOVs had a ω of 24.4–49.5 nm, while Er_2_O_3_-doped MOVs had a broader ω of 42.6–113 nm (as shown in Figure 21). The Er_2_O_3_-doped MOVs prepared by TSS had the highest Φ_B_ (0.77 eV), broadest barrier width (ω = 113 nm), and the lowest N_d_ (0.06 × 10^18^ cm^−3^) and N_s_ (0.64 × 10^12^ cm^−2^). These MOVs exhibited the highest nonlinearity (α = 154 ± 18) [63]. This behavior occurred due to the increased production of minority carriers (holes) by the impact ionization of the valence and acceptor states in the depletion regions in ZnO grains, followed by a decrease in Φ_B_ at the GBs [190].

The tendency to improve the nonlinearity behavior of 0.5 mol.% Er_2_O_3_-doped ZVMN varistors prepared by a SSS process [169,174] was similar to that observed in the TSS process [63]. The Er_2_O_3_-doped ZVMN varistors showed a higher barrier height (Φ_B_ = 0.73 eV), broader barrier width (ω = 41.9 nm), and lower N_d_ (0.4 × 10^18^ cm^−3^) and N_s_ (1.64 × 10^12^ cm^−2^) compared to undoped ZVMN varistors (Φ_B_ = 0.71 eV, ω = 20.3 nm, N_d_ = 1.62 × 10^18^ cm^−3^, and N_s_ = 3.29 × 10^12^ cm^−2^). However, these studies [63,169,174] did not assess the aging behavior of the synthesized MOVs, which is necessary to determine their electrical stability under specific stress conditions.

The ZnO-V_2_O_5_-based varistors obtained from the ZVMN systems doped with 0.5 mol.% Er_2_O_3_ that were prepared by Roy et al. [63] using a TSS process exhibited superior electrical properties compared to those of the ZnO-Bi_2_O_3_-based varistors prepared by Gunnewiek et al. [100] using a microwave two-step sintering (MW-TSS) fast process (T_1_ = 1100 °C, t_1_ = 1 min, and T_2_ = 850 °C, t_2_ = 1 h). The magnetron of the MW oven was operated at a frequency of 2.45 GHz and a power of 2 kW. The designed composition of MOVs (mol.%) was the same as Matsuoka’s formulation [6] (ZnO-0.5% Bi_2_O_3_-1% Sb_2_O_3_-0.5% CoO-0.5% MnO-0.5% Cr_2_O_3_). The fine-grained microstructure with mean ZnO grains ≤ 2.1 μm and high relative density (RD of 96%) contributed to the achievement of good varistor properties (E_B_ = 10.7 kV/cm, α = 40, I_L_ = 58 µA). Although MOVs obtained by MW-TSS processes have shown promising results, they are generally applied on a laboratory scale.

The study in [124] investigated the impact of 0.025–0.1 mol.% Bi_2_O_3_ doping on the electrical properties of ZVM* systems sintered in air at 825 °C for 3 h. The results showed that all Bi_2_O_3_-doped ZVM* varistors demonstrated superior E–J characteristics (E_B_ ≅ 3.4–10.3 kV/cm, α = 24.9–46.6, and J_L_ = 0.043–0.098 mA/cm^2^) compared to undoped ZVM* systems (E_B_ ≅ 4.8 kV/cm, α = 5.4, and J_L_ = 0.611 mA/cm^2^). As the Bi_2_O_3_ content increased from 0.025 mol.% to 0.1 mol.%, the E_B_ and α values of the doped ZVM* systems decreased, with the lowest J_L_ observed at 0.05 mol.% Bi_2_O_3_ doping. The highest E_B_ and α values were attained at 0.025 mol.% Bi_2_O_3_ doping.

Table 4 summarizes the electrical properties (E_B_, α, and J_L_) of selected ZnO-0.5 mol.% V_2_O_5_-based varistors before and after pulse aging stress. The aging was induced by applying a multi-pulse surge current (I_s_) of 10–200 A with an 8/20 μs pulse current waveform.

The study described in [161] investigated the effect of sintering temperatures ranging from 875 °C to 950 °C on the electrical properties of ZVM*NE systems, which are ZVM* systems doped with 0.05 mol.% Nb_2_O_5_ and 0.05 mol.% Er_2_O_3_. The investigation was conducted both before and after pulse degradation caused by surge currents (I_s_) ranging from 10 A to 200 A. The MOVs were subjected to surge currents with a standard 8/20 μs pulse current waveform. The surge currents were gradually applied three times to the same MOVs, with a 10-min interval between each application, using a surge generator.

The clamping voltage ratio (*K*) is determined as the ratio of the clamping voltage (*V_c_*) to *V_1mA_* (the voltage of MOV at 1 mA), as shown in Equation (31) [60,161,184]. 

The nonlinear coefficient in the high-current region (α_2_) is computed using Equation (32), where *V_c1_* and *V_c2_* are the clamping voltages corresponding to the peak currents I_p1_ = 1 A and I_p2_ = 10 A [161]:

(31) $$K=V_{c}/V_{1mA}$$

(32) $$\alpha_{2}=1/\left(\mathrm{lg}V_{c2}-\mathrm{lg}V_{c1}\right)$$

The study in [161] observed a nearly linear decrease in E_B_ with increasing sintering temperature from 875 °C to 950 °C, which was attributed to the lower number of generated grain boundaries (N_gb_) caused by the increase in the mean ZnO grain size from 6.3 µm to 16.6 µm (Table S1). The ZVMNE-based varistors sintered at 900 °C exhibited the best E–J characteristics in the low-current region, with E_B_ ≅ 4.2 kV/cm, α_1_ = 45.6, and J_L_ = 0.24 mA/cm^2^ (Table 4). These ceramic varistors demonstrated good clamp characteristics in the nonlinear region (V_c_ = 800–1060 V, and K = 1.84–2.44) at surge currents of 1–25 A, indicating a high impulse absorption capability [161].

The ZVM*NE ceramic varistors sintered in air at 925 °C exhibited the best electrical stability, with ΔE_B_/E_B_ of −10.6%, Δα_1_/α_1_ of −37.2%, and ΔJ_L_/J_L_ of 10.2%, even after applying a surge current of 200 A, without damaging the varistors (Table 4). However, the MOVs sintered at 900 °C and 950 °C were damaged after applying a surge current of 50 A, while the MOVs sintered at 875 °C were damaged after applying a surge current of 100 A [161]. Therefore, the sintering temperature variations had a significant impact on the surge stability of the synthesized MOVs.

A study conducted on ZVM* systems doped with 0.025 mol.% Er_2_O_3_ (ZVM*E systems) and sintered at 850–925 °C for 3 h reported similar results [179]. The decrease in E_B_ with an increase in sintering temperature was attributed to the reduction in GB, resulting from an increase in the mean ZnO grain size from 6.1 µm to 8.7 µm (Table S1) and a decrease in breakdown voltage drop per GB (V_gb_) from 2.3 V/GB (at 850 °C) to 0.7 V/GB (at 900 °C). The variation in grain size and V_gb_ after pulse aging was likely similar to that in the initial state, but this study does not provide exact values for these parameters.

Nahm [179] identified the optimal sintering temperature for ZVM*E-based systems at 925 °C, as they yielded good varistor properties before and after pulse aging (E_B_ ≅ 2.4 kV/cm, α_1_ = 30, J_L_ = 0.20 mA/cm^2^ in the initial state, and E_B_ ≅ 2.2–2.3 kV/cm, α_1_ = 13.6–19.6, J_L_ = 0.30–0.36 mA/cm^2^ in the stressed state), as shown in Table 4. Despite this, the stability slightly decreased with the increase in the pulse current from 10 A to 25 A, applied five times in both cases [179]. The ZVM*E systems sintered at 850 °C yielded the best impulse clamping characteristics (K = 2.22–2.88) for a pulse current of 1–25 A, indicating a good impulse absorption capability [179]. This behavior was caused by the more significant nonlinear coefficient in the high-current region (α_2_ = 13.6) than in the low-current region (α_1_ = 4.6). However, these ceramic varistors exhibited the highest leakage current density of 0.63 mA/cm^2^ in both the initial and stressed states, indicating poor electrical behavior.

The effect of 0.05–0.25 mol.% Er_2_O_3_ doping on the E–J characteristics and impulse clamping properties of ZVM* systems sintered at 900 °C for 3 h was investigated in [184]. This study found that ZVM*E systems exhibited good varistor properties in the nonlinear region when the Er_2_O_3_ content was 0.05–0.1 mol.% (E_B_ ≅ 1.2–1.8 kV/cm, α_1_ = 24.2–30, J_L_ = 0.11–0.21 mA/cm^2^) (Table 4). The highest Er_2_O_3_ content (0.25 mol.%) caused a significant decrease in nonlinearity (α_1_ = 4.8) and a high increase in leakage current density (J_L_ = 0.60 mA/cm^2^), indicating poor electrical behavior. The increase in E_B_ with increasing Er_2_O_3_ content was attributed to an increase in N_gb_, resulting from a decrease in ZnO grain size (refer to Table S1). The nonlinear coefficient in the high-current region (α_2_) gradually increased from 4.8 to 12.8 with increasing Er_2_O_3_ content. Conversely, the clamping voltage ratio (K) decreased from 2.68 to 2.01 at a pulse current of 1 A, and from 4.35 to 2.41 at a pulse current of 10 A, as the Er_2_O_3_ content increased. Therefore, varistors doped with 0.25 mol.% Er_2_O_3_, with the highest α_2_ (12.8), exhibited a good impulse absorption capability.

The clamping and pulse aging characteristics of ZVMN systems doped with 2 mol.% MnO_2_ and 0.1 mol.% Nb_2_O_5_ and sintered at 875–950 °C were investigated in [60]. This study revealed behavior similar to that observed in ZVM* systems. In the low-current region, the E_B_ decreased from 6.83 kV/cm to 0.97 kV/cm, and the J_L_ decreased from 0.19 mA/cm^2^ to 0.08 mA/cm^2^, with the increasing sintering temperature from 875 °C to 950 °C.

Table 5 demonstrates that ZnO-V_2_O_5_ systems with lower amounts of MO additives exhibit comparable or even superior electrical characteristics to the selected ZnO-Bi_2_O_3_-based systems when sintered at lower temperatures. This suggests that manufacturers can use less MO dopants and lower sintering temperatures to produce high-performance ZnO-V_2_O_5_-based varistors, leading to savings in raw materials and energy costs [59].

#### 5.3.2. Dielectric Properties of MOVs from ZnO-V_2_O_5_ Systems

The dielectric properties of MOV ceramics are typically measured using an impedance–capacitance–resistance (LCR) meter across a wide frequency range, from a few hundred Hz to several MHz. The properties, such as the apparent dielectric constant ($\varepsilon_{APP}^{\prime }$) and dissipation factor (tanδ), are usually reported at 1 kHz, as shown in Table 6.

ZnO-based varistors exhibit significant capacitive behavior below the threshold or breakdown voltage (V_B_) at a current of 1 mA. The dielectric properties of MOVs are primarily generated by depletion layers with a small width of around 100 nm at the GBs [56].

The apparent dielectric constant ($\varepsilon_{APP}^{\prime }$) and dissipation factor (tanδ) are highly influenced by the frequency and dopant content, as well as by the sintering temperature, as shown in Table 6. The ratio between the mean ZnO grain size (d) and the depletion layer width (t) on both sides of the GBs mainly affects the variation in $\varepsilon_{APP}^{\prime }$ of ZnO-V_2_O_5_ based systems. This finding is consistent with Equation (33) [56,184], where ε_g_ represents the dielectric constant of ZnO (ε_g_ = 8.5):

(33) $$\varepsilon_{APP}^{\prime }=\varepsilon_{g}\cdot d/t$$

The dissipation factor or dielectric loss tangent (tanδ) is determined as the ratio between the imaginary part ($\varepsilon_{r}^{\prime \prime }$) and the real part ($\varepsilon_{r}^{\prime }$) of the relative permittivity ε_r_:

(34) $$\tan\delta =\varepsilon_{r}^{\prime \prime }/\varepsilon_{r}^{\prime }$$

**Figure 22:**
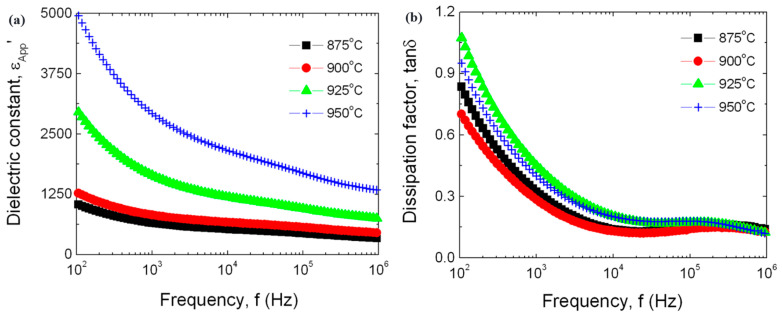
Variation in (**a**) the dielectric constant ($\varepsilon_{APP}^{\prime })$ and (**b**) dissipation factor (tanδ) with the frequency of MOVs from the ZVMND systems sintered in air between 875 °C and 950 °C. Reprinted from reference [181], published under an open access Creative Commons Attribution (CC BY 3.0) license (https://creativecommons.org/licenses/by-nc/3.0/ (accessed on 18 October 2022)). Copyright © 2015 KIEEME.

The apparent dielectric constant ($\varepsilon_{APP}^{\prime }$) typically decreases as the frequency increases from 100 Hz to 2 MHz [27,181,184]. However, the variation rate differs due to the dielectric polarization phenomenon, as dipole rotation is dependent on frequency. Figure 22 [181] provides examples of plots illustrating the variations in the dielectric properties ($\varepsilon_{APP}^{\prime }$ and tanδ) with the frequency of MOVs from the ZnO-V_2_O_5_-based systems.

According to Equation (33), an increase in the d/t ratio between the mean ZnO grain size (d) and the depletion layer width (t) of both sides at the GBs leads to an increase in the $\varepsilon_{APP}^{\prime }$. A similar trend as for the $\varepsilon_{APP}^{\prime }$ was observed for the dissipation factor (tanδ), which decreased over a broad frequency range from 100 Hz to around 10–30 kHz [27,181,184]. However, a dielectric absorbance peak typically occurs in the 200–400 kHz frequency range. The behavior of tanδ can be explained by the dielectric loss due to Joule heating caused by the leakage current and the friction heating caused by dipole rotation.

The ZnO-V_2_O_5_-based systems doped with Mn_3_O_4_ and Er_2_O_3_ [179] or Nb_2_O_5_ [50], as well as the ZVMN systems [180] doped with Dy_2_O_3_ [181] or Bi_2_O_3_ + Co_3_O_4_ + Dy_2_O_3_ [183], exhibited an increase in the $\varepsilon_{APP}^{\prime }$ at 1 kHz with an increase in the sintering temperature from 800 °C to 925 °C. This phenomenon is a consequence of the polarization of dielectric materials due to the decrease in the number of dipoles. The ZVMND and ZVMNBCD systems [181,183] sintered at lower temperatures (800–900 °C) yielded lower tanδ values at 1 kHz than similar MOVs sintered at higher temperatures (925–950 °C) [36] (Table 6).

The addition of 0.05 mol.% Dy_2_O_3_ to the ZVMN systems resulted in a change in the dielectric properties of all varistors sintered between 875 °C and 950 °C [181] (Figure 22). The $\varepsilon_{APP}^{\prime }$ at 1 kHz of the ZVMND systems increased from 658.6 to 2062.6 with an increase in the sintering temperature from 875 °C to 950 °C [181], exhibiting similar behavior to undoped ZVMN varistors [180]. The tanδ at 1 kHz of the ZVMND systems varied from 0.284 to 0.45 with an increase in the sintering temperature from 900 °C to 925 °C.

The ZVMNBCD systems [183] yielded an increase in the $\varepsilon_{APP}^{\prime }\;$at 1 kHz from 330.6 to 1391.4 with an increase in the sintering temperature from 800 °C to 925 °C, whereas the tanδ at 1 kHz varied within a narrow range (0.203–0.238). The introduction of 0.05 mol.% Bi_2_O_3_ and 0.5 mol.% Co_3_O_4_ dopants into the ZVMBCD systems [183] led to lower values of the dielectric characteristics at 1 kHz in the ZVMBCD systems [183] as compared to those in the ZVMND systems [181].

The dielectric properties of 0.05 mol.% Nb_2_O_5_-doped ZVM* [50] and 0.1 mol.% Nb_2_O_5_-doped ZVM* systems [180] sintered between 875 °C and 950 °C exhibited fluctuating behavior with the change in the sintering temperature. The varistors sintered at 900 °C and 925 °C showed similar behavior, with the $\varepsilon_{APP}^{\prime }$ at 1 kHz of 1054.9 [50] and 961.8 [180], which are comparable to the $\varepsilon_{APP}^{\prime }$ of about 1000 reported by Eda [56] for a typical ZnO-Bi_2_O_3_-based varistor. Additionally, these MOVs yielded the lowest tanδ at 1 kHz of 0.145 [50] and 0.134 [180], respectively, suggesting that these temperatures are optimal.

Nahm [179] reported that the dielectric properties of ZVM*-based systems doped with 0.025 mol.% Er_2_O_3_ changed with varying sintering temperatures (850–925 °C). The $\varepsilon_{APP}^{\prime }$ at 1 kHz increased from 1223.8 to 2239.8 as the sintering temperature increased from 850 °C to 900 °C, but decreased to 1489.3 for the MOVs sintered at 925 °C, possibly due to an increase in the depletion layer width. The increase in sintering temperature caused a decrease in the tanδ at 1 kHz from 0.543 to 0.273 (Table 6), which is comparable to the J_L_ behavior. A sintering temperature of 925 °C was found to be optimal for achieving good dielectric characteristics similar to those of commercial ZnO-Bi_2_O_3_-based varistors.

The $\varepsilon_{APP}^{\prime }$ at 1 kHz of 0.05–0.25 mol.% Er_2_O_3_-doped ZVM* systems sintered in air at a temperature of 900 °C decreased from 2104.2 (undoped MOVs) to 1314.1 (Er_2_O_3_-doped MOVs) [184] with increasing Er_2_O_3_ content. The reduction in $\varepsilon_{APP}^{\prime }$ was caused by a decrease in the number of dipoles. Similarly, 0.025–0.25 mol.% Yb_2_O_3_-doped ZVM* systems sintered in the same conditions yielded analogous behavior [27]. The $\varepsilon_{APP}^{\prime }$ at 1 kHz decreased from 2438.3 (undoped MOVs) to 1061.7 (Yb_2_O_3_-doped MOVs) with increasing Yb_2_O_3_ content. The MOVs doped with 0.25 mol.% Yb_2_O_3_ yielded the lowest $\varepsilon_{APP}^{\prime }$ at 1 kHz (1061.7), which corresponds to the $\varepsilon_{APP}^{\prime }$ (~1000) reported by Eda [56] for a commercial ZnO-Bi_2_O_3_-based varistor. Nevertheless, MOVs doped with 0.1 mol.% Yb_2_O_3_ showed the highest stability against DC aging stress.

The undoped ZVM* systems sintered in air at 900 °C for 3 h exhibited different values of the dissipation factor at 1 kHz, with a tanδ of 0.424 in reference [27] and 0.354 in reference [184], due to variations in the physical and electrical characteristics of the synthesized MOVs. Furthermore, the tanδ at 1 kHz increased with the REO content increase, from 0.297 to 0.468 [184] for 0.05–0.25 mol.% Er_2_O_3_-doped MOVs, and from 0.309 to 0.482 [27] for 0.025–0.25 mol.% Yb_2_O_3_-doped MOVs (Table 6). However, the undoped ZVM* systems sintered at a lower temperature (825 °C) yielded a higher tanδ (0.506) and lower $\varepsilon_{APP}^{\prime }$ (1324.5) at 1 kHz [124] compared to analogous ZVM* systems sintered at 900 °C [27,184].

Nahm [26] investigated the effect of Yb_2_O_3_ doping on the dielectric properties of 0.05–0.25 mol.% Yb_2_O_3_-doped ZVMN systems sintered at 900 °C. The $\varepsilon_{APP}^{\prime }$ at 1 kHz of the ZVMNY systems showed a slight variation in the range of 549.1 to 592.7 as the Yb_2_O_3_ content increased, with a similar trend corresponding to the change in the ZnO grain size. However, the dissipation factor (tanδ) increased from 0.209 to 0.313 with the increase in dopant content. The change in tanδ was related to the increase in leakage current density, which increased from 0.057 μA/cm^2^ to 0.252 μA/cm^2^ with the increase in Yb_2_O_3_ content.

In another study by Nahm [24], the influence of the Dy_2_O_3_ content on the dielectric properties of ZVMN systems sintered at 900 °C was investigated. The $\varepsilon_{APP}^{\prime }$ value at 1 kHz of the ZVMN systems varied from 713.1 to 917.6, while the dissipation factor (tanδ) ranged between 0.205 and 0.355, with the highest tanδ corresponding to the most increased leakage current density (J_L_) of 0.42 mA/cm^2^. The lowest $\varepsilon_{APP}^{\prime }$ and tanδ values at 1 kHz were observed for 0.1 mol.% Dy_2_O_3_-doped MOVs, indicating the best dielectric characteristics for this formulation of MOVs, similar to other REO doping ZVMN systems [26].

Additionally, reference [124] investigated the effect of 0.025–0.1 mol.% Bi_2_O_3_ doping on the dielectric properties of the ZVM* systems sintered at 825 °C for 3 h. The undoped MOVs exhibited higher values of the dielectric characteristics at 1 kHz ($\varepsilon_{APP}^{\prime }$ = 1324.5, and tanδ = 0.506) than those of the Bi_2_O_3_-doped MOVs ($\varepsilon_{APP}^{\prime }$ = 528.3–734.4, and tanδ = 0.176–0.225). The lowest dielectric constant at 1 kHz ($\varepsilon_{APP}^{\prime }$ = 528.3) was observed for 0.025 mol.% Bi_2_O_3_-doped MOVs, while the lowest dissipation factor at 1 kHz (0.176) was achieved for 0.05 mol.% Bi_2_O_3_-doped MOVs.

Nahm [36] investigated the stability of dielectric properties of ZnO-based MOVs containing 0.5 mol.% V_2_O_5_ and 2 mol.% MnO_2_ (referred to as ZVM systems) sintered in air at temperatures ranging from 800 °C to 950 °C. The measurements were performed between 100 Hz and 2 MHz on both initial and stressed MOVs after conducting DC-accelerated aging (0.85 × E_B_ at 85 °C for 24 h). The values of the Δ$\varepsilon_{APP}^{\prime }$/$\varepsilon_{APP}^{\prime }$ (%) and Δtanδ/tanδ (%) were determined using Equations (35) and (36), as shown in Table 7:

(35) $$\Delta \varepsilon_{APP}^{\prime }/\varepsilon_{APP}^{\prime }=[({\varepsilon_{APP}^{\prime }}_{\mathrm{stressed}}-{\varepsilon_{APP}^{\prime }}_{\mathrm{initial}})/{\varepsilon_{APP}^{\prime }}_{\mathrm{initial}}]\times 100$$

(36) $$\Delta \tan\delta /\tan\delta =[({\tan\delta }_{\mathrm{stressed}}-{\tan\delta }_{\mathrm{initial}})/{\tan\delta }_{\mathrm{initial}}]\times 100$$

The ZVM-based varistors sintered in air at 800–850 °C showed lower $\varepsilon_{APP}^{\prime }$ and tanδ values compared to those of the MOVs sintered at higher temperatures (900–950 °C) [36] (Table 7). The $\varepsilon_{APP}^{\prime }$ of all MOVs decreased as the frequency increased, which is consistent with the polarization phenomenon observed in dielectric materials. Additionally, the $\varepsilon_{APP}^{\prime }$ increased as the sintering temperature increased from 800 °C to 900 °C due to a decrease in the number of dipoles.

The ZVM systems sintered at 900 °C exhibited high stability, with the lowest variation in the dissipation factor (Δtanδ/tanδ = 21.8% at 1 kHz) (Table 7). Minor changes were noticed in $\varepsilon_{APP}^{\prime }$ at 1 kHz (1163.5 in the initial state, and 1160 in the stressed state) and in tanδ at 1 kHz (0.316 in the initial state, and 0.385 in the stressed state), because the ZnO grain size and depletion layer width were not modified after conducting DC aging stress [36]. This indicates that the optimal sintering temperature for achieving good dielectric stability in MOVs is 900 °C.

### 5.4. Factors Inducing the Failure Modes of ZnO-Based Varistors for Surge Arresters

Gapless MO surge arresters (MOSAs) are prone to depreciation and damage over time due to different causes, such as high energy stresses, housing deterioration, and the gradual degradation of MOV discs that are subjected to the operating voltage, impulse voltage, and environmental stresses [75,191,192]. 

One significant type of damage to MOV discs is thermal cracking, which can occur as a result of multiple short or long-lasting temporary overvoltages (TOVs), either during or after clearing lightning strikes or high-current surges, or due to the development of mechanical fractures from thermal runaway [72].

Flashover events between the MOV discs and the side walls of the porcelain housing can lead to electrical puncture or physical crackdown and, subsequently, to a short-circuit inside the housing. This can result in a high ground fault current through it, which can increase the internal temperature and the pressure of the housing [75,192]. Insufficient contact between the circular surfaces of MOV discs can lead to localized losses and discharging, whereas housing deterioration or pollution negatively impacts the voltage distribution along the stack of MOV discs.

Inadequate sealing of the housing can result in moisture ingress inside the surge arresters [191]. The concentration of the surge current at the edge of the metal electrodes can also cause the failure of MOSAs. Deterioration or damage to MOVs results in an increase in the resistive leakage current and a decrease in the dielectric strength of MOVs, which affects the performance of surge arresters equipped with noncompliant MOVs. Therefore, it is imperative to develop reliable MOVs for use in electric power systems and in industrial and consumer electronics and to ensure the adequate monitoring of the condition of MOVs and surge arresters for the safe operation of equipment protected against temporary overvoltages and current surges [193].

The geometry of MOVs has a significant impact on their failure due to high-current pulse-induced fracture. Lengauer et al. [194] identified an optimal thickness to diameter ratio of approximately 0.9 for MOV discs to minimize tensile stresses, which are more likely to cause failure than compressive stresses when the disc thickness is much greater than the disc diameter. Therefore, designing a suitable aspect ratio and increasing the strength of MOVs is necessary since MOVs can fail in service due to pulse-induced fracture, electrical puncture, or the long-term deterioration of the electrical properties [3,194].

There have been several experimental studies aimed at determining the failure modes of ZnO-based varistors used in surge arresters under relevant environmental testing conditions involving multiple lightning strikes or radiation damage. However, these studies lack sufficient information on technical characteristics such as the chemical composition and size of MOV discs, as well as the suppliers of commercial ZnO-based varistors [71,195,196,197,198]. On the other hand, these studies provide relevant findings on the failure mechanisms of MOVs subjected to multiple lightning strikes or radiation exposure (e.g., radium (Ra)-226 or californium (Cf)-252 and neutron/gamma radiation, etc.).

For MOSAs subjected to multiple lightning strikes, it was observed that after the impulse heat was absorbed, there was a temperature increase (gradient) in the local area, resulting in thermal damage and deterioration in the GB structure due to the applied thermal stress caused by repetitive lightning impulse currents [195,196]. 

For MOVs subjected to Cf-252 and neutron and gamma (n + γ) radiation, or gas-filled surge arresters (GFSAs) subjected to γ radiation with the Ra source, it was demonstrated that MOV devices are sensitive to n + γ radiation. As a result, electrical characteristics such as the breakdown voltage (V_B_) and nonlinear coefficient (α), as well as the radiation protection characteristics of MOV devices, can degrade due to a decrease in electrical conductivity and an increase in the local electric field in the vicinity of dislocations [197]. Nevertheless, several commercial GFSAs were found to be effective against γ radiation under certain conditions [198].

### 5.5. Strategies to Improve the Characteristics of MOVs from ZnO-V_2_O_5_-Based Systems

Based on the reviewed literature reports, the electrical characteristics, GB-related parameters, and dielectric characteristics of MOVs from the ZnO-V_2_O_5_-based systems varied depending on the type and content of the V_2_O_5_ and MO dopants used in these systems. The study results also showed that the pressing pressure, sintering temperature, and dwell time, as well as the formation of secondary phases and their content, had a significant effect on the microstructures and properties of ZnO-V_2_O_5_-based varistors. Properly selecting the powder metallurgy (PM) parameters can lead to superior microstructural features, which can have positive effects on the performance of MOVs.

The size of grains and the number of grain boundaries (GBs) in the microstructures of MOVs, along with the intergranular effects, play a crucial role in altering the varistor properties, according to several reviewed literature reports [63,80,199]. However, controlling the grain size and grain size distribution in multicomponent ZnO-based varistors is a challenging task due to the many interdependent MO dopants needed to achieve the desired varistor characteristics [59].

The ZnO grain size determines the electrical breakdown (E_B_) of MOVs for a specific formulation and thickness of the MOV disc [80]. The number of GBs across the thickness of an MOV disc increases with the reduction in grain size during sintering, which improves the E_B_. Therefore, these factors are essential to consider when designing MOVs using ZnO-based systems. For example, if a MOV device requires a switching voltage of 300 V, approximately 100 grains of ZnO need to be connected in series, taking into account a switching voltage of about 3 V for a single GB [3]. If the mean ZnO grain size is around 20 μm, then the corresponding MOV disc thickness is 100 × 20 μm = 2000 μm = 2 mm [3].

The optimum ratio between dopant content (DC) and GB area (S) is a key performance indicator (KPI) in evaluating the electrical properties of MOVs. However, determining the optimal DC/S ratio usually requires experimentation. When the sintering temperature or dwell time is inadequate, the mean ZnO grain size (d_ZnO_) decreases, leading to an increase in the GB area, while an increase in d_ZnO_ leads to a decrease in the GB area.

An efficient strategy for improving the non-ohmic behavior of multiternary ZnO-based systems consists of developing novel compositions with appropriate types and content of VFO and MO dopants, using adequate PM processing parameters. A better microstructure can be achieved by reducing the ZnO grain size, obtaining a narrow ZnO grain size distribution, and improving the uniformity and long-term stability of electrical potential barriers (DSBs) across the GBs [33,80,83,200]. The use of IB-forming dopants can tailor the microstructure and the electronic and optical characteristics of ZnO-based systems, benefiting from enhanced functional properties at reduced costs [59].

When selecting the optimal sintering conditions for the manufacturing of ZnO-V_2_O_5_-based varistors, it is crucial to consider the effect of V_2_O_5_ volatility. This factor can significantly impact various characteristics, including the microstructure, the growth of the ZnO grain size, secondary phase formation, sintered density, and GB parameters.

The concentration of metal ions in the MO dopants is a critical factor that determines the formation of GB barriers (DSBs) in ZnO-based varistors [147,160]. A suitable concentration of dopants distributed uniformly along the GBs can enhance the varistor performance. Conversely, an uneven or excessive concentration of dopants may destroy the GB barriers and structure, leading to a decline in varistor performance. The precise control of the MO dopant content is essential to achieving the desired properties in MOVs.

Defect engineering, such as self-doping with specific point defects or doping with transition, rare element (RE), or other metal ions is a practical approach to develop high-performance ZnO-based varistors with enhanced electrical properties and prolonged stability [33,138]. By introducing controlled imperfections into the crystal lattice of ZnO from MOV compositions, the band gap and electronic arrangement can be tuned, and the content and mobility (µ) of charge carriers can be improved by restraining mobile Z_ni_ and simultaneously increasing the V_O_ density.

The degradation rate of the MOVs from the ZnO-based systems can be reduced when the Zn_i_ density is lower, since Zn_i_ represents the major intrinsic point defect species that generates the electrical degradation of MOVs under DC or AC electrical and thermal stresses [159,201]. Additionally, the Zn_i_ density is an effective parameter for investigating the electrical degradation of ZnO-based varistors at a macroscopic level. Therefore, microstructural aspects, such as the grain size, number of GBs, electrical potential barriers (DSBs), and defects in the ZnO crystal structure, are considered in the degradation and failure mechanisms of MOVs to explain their protective roles [199].

## 6. Conclusions

In conclusion, this review provides an overview of the progress that has been made in metal oxide varistors (MOVs) from ZnO-V_2_O_5_ systems that are doped with 1–5 MOs. The study highlights the use of powder metallurgy (PM) techniques for preparing advanced ceramic materials for MOVs from the ZnO-V_2_O_5_ systems with comparable or superior properties to ZnO-Bi_2_O_3_ systems but with less dopants. Most research works have been conducted on small-sized MOV discs with a diameter × height of Ø8–20 mm × 1–4 mm, obtained from ball-milled MOV powders via the PM route for ceramic materials. Although extensive research has been done on the microstructure and structural and E–J characteristics of ZnO-V_2_O_5_-based varistors, there is still an insufficient correlation between the properties of MOV powders and the resulting MOV discs. Additionally, the dielectric characteristics and aging behavior are less often investigated. Nonetheless, the ZnO-V_2_O_5_-based systems with 0.25–2 mol.% V_2_O_5_ and MO additives sintered in air over 800 °C contain a primary phase of ZnO with a hexagonal wurtzite structure and several secondary phases (e.g., Zn_3_(VO_4_)_2_, ZnV_2_O_4_, Zn_2_V_2_O_7_, Zn_4_V_2_O_9_, RE-related phases, etc.) that influence the ZnO grain growth behavior and MOV performance. The microstructures and properties of the MOVs from the ZnO-based systems doped with 0.5–2 mol.% V_2_O_5_, 2 mol.% MnO_2_ or 0.5 mol.% Mn_3_O_4_, and 0.025–0.5 mol.% MOs (Bi_2_O_3_, In_2_O_3_, Sb_2_O_3_, TEOs, and REOs) have been shown to exhibit microstructure homogeneity, high density, and superior varistor properties. Refining and homogenizing the microstructures of MOVs can improve their electrical properties and stability. This can be achieved by doping MOVs with a small amount (0.025–0.5 mol.%) of grain growth inhibitors (REOs, Bi_2_O_3_, and In_2_O_3_) and consolidating them under suitable PM conditions. This process increases the number of GBs across the thickness of the MOV disc, which improves its behavior by increasing the Schottky barrier height (Φ_B_) in the GBs. The resulting MOVs exhibit satisfactory electrical properties, such as a low leakage current density (J_L_) of 0.02–0.2 mA/cm^2^, a high nonlinear coefficient (α) of 22–153, and a high breakdown field (E_B_) of 2–14 kV/cm. Studies on the grain growth kinetics during the LPS of ZnO-V_2_O_5_ systems have shown that doped MOVs exhibit a higher kinetic grain growth exponent and apparent activation energy than undoped ZnO ceramics. Furthermore, several studies have revealed the underlying mechanisms of sintering and aging, as well as the degradation processes, in ZnO-V_2_O_5_-based varistors. These findings suggest that high-performance MOVs from ZnO-V_2_O_5_-based systems have great potential for practical applications.

## 7. Future Research Directions

The current focus of studies on ZnO-V_2_O_5_-based systems is on the preparation of small-sized MOV discs (Ø8–20 mm × 1–4 mm) for use in voltage SPDs. The most commonly used method for obtaining these MOV discs is through PM techniques from ball-milled MOV powders with nanosized particles.

However, there is a need for research on disc-shaped MOVs of larger sizes (diameter × height of Ø20–120 mm × 20–50 mm) for several potential applications, such as surge arresters for electric power systems, high-voltage equipment (transformers and switchgear), and consumer electronics (computers, televisions, and home appliances).

To achieve large-sized MOV discs, a large amount of MOV powder needs to be prepared, and appropriate investigation methods need to be employed. Testing MOV discs in laboratory conditions and in an industrial environment during their service in surge arresters is necessary to identify suitable MOVs for practical applications. Moreover, a certain number and size of MOV discs are required to create a stack of MOVs for use in the design of gapless MO surge arresters (MOSAs).

The benefits of using large-sized ZnO-V_2_O_5_-based varistors include (i) improved performance, as large-sized MOVs can handle higher surge currents and energies, providing superior protection to equipment compared to smaller MOVs; (ii) increased reliability, as adequately designed and manufactured large-sized MOVs can ensure greater longevity and reduce the need for maintenance and replacement; and (iii) cost savings, as the use of large-sized MOVs in electrical equipment can decrease the need for additional protective measures, avoiding costly equipment failures due to voltage surges and transients.

The overall conclusions drawn from the studies on small-sized MOV discs from doped ZnO-V_2_O_5_ systems can provide valuable guidance for researchers and MOV manufacturers aiming to develop and standardize novel formulations for large-sized MOVs with appropriate selection of the constitutive elements and well-defined processing parameters. Furthermore, the main challenge is achieving superior properties of MOVs at comparable or lower production costs compared to the commercial ZnO-Bi_2_O_3_-based varistors, given the differences in size and properties between small- and large-sized MOVs.

It is crucial to develop high-performance disc-shaped ZnO-V_2_O_5_-based varistors for use in SPDs, MOSAs, and GFSAs, and to properly monitor the condition of the MOV discs to ensure the safe and adequate operation of MOVs and related equipment protected by MOVs. The achievement of more reliable and cost-effective large-sized MOVs from ZnO-V_2_O_5_-based systems with fewer additives can ensure their continued effectiveness in protecting equipment from overvoltage and reducing downtime and maintenance costs.

Besides experimental works, additional research studies involving modeling and simulation are necessary to predict the microstructures and various properties of MOVs. These studies can lead to more reliable MOVs and validated technologies for industrial applications. Further collaboration between researchers and industry professionals is crucial to ensure that research is translated into practical application. By working together, researchers and industry professionals can identify and address the most important challenges in the development and implementation of MOV technology, and accelerate the adoption of these critical safety devices. The growing demand for MOVs with enhanced properties for use in industrial equipment and consumer electronics, along with the expansion of the global market for MOV discs, underscores the great potential for the development of novel advanced ceramic materials for MOVs.

## Figures and Tables

**Table 1:** Critical parameters and functions of MOV devices (adapted from [65]).

Parameter	Function	Comments	Reference
Nonlinear coefficient (α)	Protective level	Typical α values in the low-current region are 20–70. The increase in temperature and pressure to which the MOV device is subjected in service causes a decrease in α values.	[57,65]
Nonlinear voltage (V)	Voltage rating	It is the threshold or breakdown voltage (V_B_) at a current of 1 mA. Typical E_B_ values are in the range of 2–5 kV/cm.	[8,65]
Leakage current (I_L_)	Watt loss/operating voltage	DC I_L_ values ≤ 100 μA for small-sized MOV discs, and ≤200 μA for large-sized MOV discs equipping electric power and telecommunication SPDs; AC I_L_ = I_R_ + I_C_.	[54,65,66]
Lifetime	Stability	Generated power (P_G_) < dissipated power (P_D_).	[65]
Energy absorption capability (E)	Survival of the electrical components	E depends on the size of MOV discs; a high surface-to-volume ratio of the MOV discs leads to a high E.	[65,66]

**Table 2:** Preparation methods of EMO and MOV powders, along with their advantages and disadvantages [54,70,85,86,87,88,89,90,91,92].

Preparation Methods of EMO and MOV Powders	Advantages	Disadvantages
(i) Solid-phase methods: ▪ Mechanical homogenization/milling of constitutive powders by using tumbling ball/rod mills, low- or high-energy planetary ball mills, high-speed vibration ball mills, attrition mills, or cryogenic mills	✓ Extensive use on a laboratory and industrial scale; ✓ Relatively inexpensive production costs; ✓ Broad range of nano- or microparticle sizes and MO suppliers	➢ Difficulties in obtaining a uniform and narrow grain size distribution; ➢ Inadequate compositional homogeneity of powder mixtures; ➢ Powder contamination from the milling tools, atmosphere, among others; ➢ High milling time
(ii) Liquid-phase methods: ▪ Chemical methods (sol–gel, combustion synthesis, precipitation or coprecipitation, and subsequent calcination of powders); ▪ Solvent vaporization (atomization and spray drying, flame spray pyrolysis, freeze-drying); ▪ Metal–organic polymeric methods, microemulsion (iii) Gas-phase methods: Chemical vapor oxidation methods	✓ Some methods involve industrial scalability; ✓ Reasonable control of shape and chemical composition; ✓ High purity of the synthesized powders; ✓ Adequate homogeneity of composite powders	➢ Expensive production costs; ➢ Challenges in the mass production of powders at low costs; ➢ Wet-chemical methods require large volumes of liquid; ➢ Distinct methods need a significant number of additional stages; ➢ Occurrence of powder agglomeration in some conditions; ➢ Difficulties in controlling evaporation rate and grain growth rate; ➢ Specific equipment for synthesis

**Table 3:** Variation in the electrical properties (E_B_, α, and J_L_) of selected ZnO-0.5 mol.% V_2_O_5_-based systems before and after conducting DC-accelerated aging stress (0.85 × E_B_ at 85 °C for 24 h).

Content of MO Additives of ZnO-0.5% V_2_O_5_ Systems (mol.%) (MOV System Type)	Sintering Temperature (t = 3 h)	Breakdown Field, E_B_ (kV/cm)			Nonlinear Exponent (α) in the Low-Current Region			Leakage Current Density, J_L_ (mA/cm^2^)			Degradation Rate Coefficient K_T_ (µA h^−1/2^)	Ref.
		Initial	Stressed	ΔE_B_/E_B_ (%)	Initial	Stressed	Δα/α (%)	Initial	Stressed	ΔJ_L_/J_L_ (%)		
0.5% Mn_3_O_4_ (ZVM*)	900 °C	1.072	0.960	**−10.4**	20	11	**−45.0**	0.17	0.48	**182.4**	12.0	[144]
2% Mn_3_O_4_ (ZVM*)		4.444	2.556	**−42.5**	6	3	**−50.0**	0.58	0.74	**27.6**	thermal runaway	
0.5% MnO_2_ (ZVM)		0.722	0.502	**−30.5**	21	5	**−76.2**	0.18	0.62	**244.4**	35.3	
2% MnO_2_ (ZVM)		0.999	0.978	**−2.1**	27	20	**−25.9**	0.042	0.21	**400.0**	3.8	
2% MnO_2_ (ZVM)	800 °C 850 °C 900 °C 950 °C	17.640 7.881 0.992 2.430	~5.3 ~6.1 0.998 ~2.2	**−** **70.0** **−** **22.6** **0.6** **−** **9.5**	38.1 17.0 27.2 32.0	2.8 ~8.5 20.1 ~22	**−** **92.7** **−** **50.0** **−26.1** **−** **31.3**	0.14 0.27 0.17 0.11	− − − −	**−** **−** **−** **−**	− − 3.8 9.0	[36]
0.5% Mn_3_O_4_ (ZVM*)	900 °C	0.922	0.880	**−4.6**	20.7	14.3	**−30.9**	0.31	−	**−**	9.4	[27]
0.5% Mn_3_O_4_ + 0.025% Yb_2_O_3_		1.025	0.943	**−8.0**	29.2	14.9	**−49.0**	0.13	−	**−**	16.0	
0.5% Mn_3_O_4_ + 0.1% Yb_2_O_3_		1.637	1.648	**0.7**	26.3	25.2	**−4.2**	0.23	−	**−**	5.5	
0.5% Mn_3_O_4_+ 0.25% Yb_2_O_3_ (ZVM*Y)		3.774	2.833	**−24.9**	5.7	3.8	**−33.3**	0.60	−	**−**	− 19.4	
0.5% Mn_3_O_4_ +0.05% Nb_2_O_5_ (ZVM*N)	875 °C 900 °C 925 °C 950 °C	5.671 3.967 2.489 1.443	0.675 3.498 0.703 1.449	**−** **88.1** **−** **11.8** **−** **71.8** **0.4**	37.9 47.0 24.2 17.8	1.2 16.0 1.8 17.1	**−** **96.8** **−** **66.0** **−** **92.6** **−** **0.6**	0.20 0.14 0.32 0.09	0.81 1.00 0.77 0.11	**305.0** **614.3** **140.6** **22.2**	− 19.6 − 3.5	[50]
2% MnO_2_ + 0.1% Nb_2_O_5_ (ZVMN)	875 °C 900 °C 925 °C 950 °C	6.991 4.800 2.241 0.943	4.305 4.389 2.274 0.603	**−38.4** **−8.6** **1.5** **−36.1**	44 50 38 25	4 20 43 5	**−90.9** **−60.0** **13.2** **−80.0**	0.2013 0.0949 0.0258 0.1737	0.7208 0.2824 0.0548 0.5095	**258.1** **197.6** **112.4** **193.3**	failure 27.6 0.38 20.8	[180]
2% MnO_2_ + 0.1% Nb_2_O_5_ + 0.5% Co_3_O_4_ + 0.1% Dy_2_O_3_ +0.05% Bi_2_O_3_ (ZVMNCDB)	850 °C 875 °C 900 °C 925 °C	8.016 4.522 2.351 1.715	7.533 4.265 2.370 1.343	**−6.0** **−5.7** **0.8** **−21.7**	44 59 45 38	21 24 45 8	**−52.3** **−59.3** **0** **−78.9**	0.0390 0.0362 0.0600 0.1006	0.1869 0.2390 0.1661 0.5457	**379.2** **560.2** **176.8** **442.4**	− − − −	[182]
0.5% Mn_3_O_4_ + 0.1% Nb_2_O_5_ + 0.5% Co_3_O_4_ + 0.1% Dy_2_O_3_ + 0.05% Bi_2_O_3_ (ZVM*NCDB)	850 °C 875 °C 900 °C 925 °C	5.919 3.370 1.871 1.465	5.798 3.249 1.640 1.204	**−2.0** **−3.6** **−12.3** **−17.8**	39.1 43.6 37.2 33.3	30.0 27.1 13.2 9.6	**−23.3** **−37.8** **−64.5** **−71.2**	0.0357 0.0282 0.0630 0.0741	0.1229 0.1592 0.4019 0.4507	**244.3** **464.5** **537.9** **508.2**	1.38 2.17 5.03 12.0	[183]

**Table 4:** Electrical properties (E_B_, α, and J_L_) of MOVs from selected ZnO-0.5 mol.% V_2_O_5_-based systems before and after pulse aging by applying a multi-pulse surge current (I_s_) of 10–200 A with an 8/20 μs waveform.

Content of MO Additives of ZnO-0.5% V_2_O_5_ Systems (mol.%) (MOV System Type)	Sintering Temperature (t = 3 h)	Aging Stress State	Breakdown Field, E_B_ (kV/cm)			Nonlinear Exponent (α) in the Low-Current Region			Leakage Current Density, J_L_ (mA/cm^2^)			Reference
			Initial	Stressed	ΔE_B_/E_B_ (%)	Initial	Stressed	Δα/α (%)	Initial	Stressed	ΔJ_L_/J_L_ (%)	
0.5% Mn_3_O_4_ + 0.025% Er_2_O_3_ (ZVM*E)	850 °C 875 °C 900 °C 925 °C	I_s_ = 10 A (5 times)	3.856 1.385 0.922 2.352	3.560 1.161 0.743 2.275	**−7.7** **−16.2** **−19.4** **−3.3**	4.6 17.9 21.3 30.0	4.4 7.9 7.3 19.6	**−4.3** **−55.9** **−65.7** **−34.7**	0.63 0.24 0.13 0.20	0.63 0.47 0.47 0.30	**0** **95.8** **261.5** **50.0**	[179]
0.5% Mn_3_O_4_ + 0.025% Er_2_O_3_ (ZVM*E)	850 °C 875 °C 900 °C 925 °C	I_s_ = 25 A (5 times)	3.856 1.385 0.922 2.352	3.225 1.066 0.601 2.150	**−16.4** **−23.0** **−34.8** **−8.6**	4.6 17.9 21.3 30.0	4.1 6.1 4.5 13.6	**−10.9** **−65.9** **−78.9** **−54.7**	0.63 0.24 0.13 0.20	0.63 0.50 0.56 0.36	**0** **108.3** **330.8** **80.0**	[179]
0.5% Mn_3_O_4_ + 0.05% Nb_2_O_5_ + 0.05% Er_2_O_3_ (ZVM*NE)	875 °C 900 °C 925 °C 950 °C	I_s_ = 25 A (3 times)	5.909 4.175 1.995 1.028	5.403 3.702 1.965 0.839	**−8.6** **−11.3** **−1.5** **−18.4**	36.4 45.6 14.5 17.8	16.3 13.7 13.2 7.7	**−55.2** **−69.9** **−9.0** **−56.7**	0.28 0.24 0.39 0.40	0.28 0.31 0.39 0.39	**0** **29.2** **0** **−2.5**	[161]
0.5% Mn_3_O_4_ + 0.05% Nb_2_O_5_ + 0.05% Er_2_O_3_ (ZVM*NE)	875 °C 900 °C 925 °C 950 °C	I_s_ = 200 A (3 times)	5.909 4.175 1.995 1.028	− − 1.784 −	**−** **−** **−10.6** **−**	36.4 45.6 14.5 17.8	− − 9.1 −	**−** **−** **−37.2** **−**	0.28 0.24 0.39 0.40	− − ~0.43 −	**−** **−** **10.2** **−**	[161]
2% MnO_2_ + 0.1% Nb_2_O_5_ (ZVMN)	875 °C 900 °C 925 °C 950 °C	I_s_ = 10 A (3 times)	6.830 4.791 2.292 0.968	6.643 4.689 2.250 0.887	**−2.7** **−2.1** **−1.8** **−8.4**	35.4 49.5 39.5 25.3	24.0 27.1 28.8 15.7	**−32.2** **−45.3** **−27.1** **−37.9**	0.19 0.11 0.03 0.08	0.21 0.17 0.11 0.21	**10.5** **54.5** **266.7** **162.5**	[60]
2% MnO_2_ + 0.1% Nb_2_O_5_ (ZVMN)	875 °C 900 °C 925 °C 950 °C	I_s_ = 25 A (3 times)	6.830 4.791 2.292 0.968	6.400 4.524 2.068 0.827	**−6.3** **−5.6** **−9.8** **−14.6**	35.4 49.5 39.5 25.3	18.7 20.2 14.7 11.2	**−47.2** **−59.2** **−62.8** **−55.7**	0.19 0.11 0.03 0.08	0.24 0.19 0.18 0.21	**26.3** **72.7** **500.0** **162.5**	[60]
2% MnO_2_ + 0.1% Nb_2_O_5_ (ZVMN)	875 °C 900 °C 925 °C 950 °C	I_s_ = 50 A (3 times)	6.830 4.791 2.292 0.968	6.205 4.292 1.999 0.778	**−9.2** **−10.4** **−12.8** **−19.6**	35.4 49.5 39.5 25.3	16.0 15.4 12.4 9.0	**−54.8** **−68.9** **−68.6** **−64.4**	0.19 0.11 0.03 0.08	0.26 0.22 0.21 0.33	**36.8** **100.0** **600.0** **312.5**	[60]
2% MnO_2_ + 0.1% Nb_2_O_5_ (ZVMN)	875 °C 900 °C 925 °C 950 °C	I_s_ = 100 A (3 times)	6.830 4.791 2.292 0.968	5.861 4.089 1.798 0.697	**−14.2** **−14.7** **−21.6** **−28.0**	35.4 49.5 39.5 25.3	13.0 13.0 8.8 6.9	**−63.3** **−73.7** **−77.7** **−72.7**	0.19 0.11 0.03 0.08	0.30 0.26 0.29 0.39	**57.9** **136.4** **866.7** **387.5**	[60]

**Table 5:** Critical electrical parameters of MOV devices from several ZnO-V_2_O_5_-based systems with good varistor properties compared to those of some relevant ZnO-Bi_2_O_3_-based systems.

MOV System Type (mol.%)	State	P_p_, T_s_/DT (PM Process)	E_B_ (kV/cm)	α	I_L_ (μA)	J_L_ (mA/cm^2^)	MOV Producers	Reference
**ZnO-V_2_O_5_-based systems**								
ZnO-0.5% V_2_O_5_-0.5% Mn_3_O_4_	Initial	80 MPa, 900 °C/ 3 h (SSS)	1.07	20	−	0.170	Semiconductor Ceramics Laboratory, Department of Electrical Engineering, Dongeui University, Republic of Korea	[144]
	Stressed		0.96	11	−	0.480		
ZnO-0.5% V_2_O_5_-0.5% Mn_3_O_4_	Initial	80 MPa, 900 °C/ 3 h (SSS)	0.72	27	−	0.042		[144]
	Stressed		0.99	20	−	0.210		
ZnO-0.5% V_2_O_5_-0.5% Mn_3_O_4_-0.1% Yb_2_O_3_	Initial	100 MPa, 900 °C/ 3 h (SSS)	1.64	26.3	−	0.230		[27]
	Stressed		1.65	25.2	−	−		
ZnO-0.5% V_2_O_5_-1% Nb_2_O_5_-0.1% In_2_O_3_	Initial	500 MPa, 875 °C/1 h (SSS)	14.2 ± 0.1	153 ± 7	−	0.062	Variable Energy Cyclotron Centre and Homi Bhabha National Institute, India	[44]
	Initial	500 MPa, 900 °C/1 h (SSS)	11.6 ± 0.2	132 ± 8	−	0.083		
ZnO-0.5% V_2_O_5_-0.5% Mn_3_O_4_-0.05% Bi_2_O_3_	Initial	100 MPa, 825 °C/3 h (SSS)	6.027	31	−	0.043	Semiconductor Ceramics Laboratory, Department of Electrical Engineering, Dongeui University, Republic of Korea	[124]
ZnO-0.5% V_2_O_5_-0.5% Mn_3_O_4_-0.1% Bi_2_O_3_	Initial		3.357	24.9	−	0.052		
**ZnO-Bi_2_O_3_-based systems**								
ZnO-1% Bi_2_O_3_-1% Sb_2_O_3_-0.75% MnO_2_-1% Co_2_O_3_-0.5% Cr_2_O_3_-1% Sb_2_O_3_, 1.2% SiO_2_-0.2% Al_2_O_3_-0.005-0.02% In_2_O_3_	Initial	~39 MPa 1200 °C/2 h (SSS)	3.56–4.34	52–60	−	0.002–0.004	State Key Laboratory of Control and Simulation of Power System and Generation Equipment, Department of Electrical Engineering, Tsinghua University, China	[43]
ZnO-0.5% Bi_2_O_3_-1% Sb_2_O_3_-0.5% CoO-0.5% MnO_2_-0.5% Cr_2_O_3_	Initial	~33 MPa 1350 °C (SSS)	1.35	50	−	−	Matsushita Electric Industrial Co., Japan (actual Panasonic)	[6]
	Initial	1100 °C/1 min and 850 °C/1 h (MW-TSS)	10.7	40	58	−	PPG-CEM, Federal University of São Carlos, Brazil	[100]
Commercial ZNR 10K 270 (ZnO-Bi_2_O_3_-MO dopants)	Initial	NA	0.22	≤11	−	0.070	Matsushita Electric Industrial Co., Japan (actual Panasonic)	[186]
Commercial ZNR 10K 470 (ZnO-Bi_2_O_3_-MO dopants)	Initial	NA	0.42	≤25	−	0.013		
Commercial GEMOV 27Z 1 (ZnO-Bi_2_O_3_-MO dopants)	Initial	NA	0.27	≤16	−	0.035	General Electric Co., USA	
Commercial (ZnO-Bi_2_O_3_ + MO dopants)	Initial	1095 °C/2.5 h (SSS)	1.85	27	17	−	Unknown producer	[120]
ZnO-Bi_2_O_3_-Sb_2_O_3_-Co_3_O_4_-Mn_2_O_3_-NiO-B_2_O_3_-Al_2_O_3_	Initial	95 MPa, 1115 °C/5 h (SSS)	1.79	56	3.4	−	Tridelta Parafoudres S.A. and UMR CNRS-UPSINP, France	[120]

Note: P_p_ = pressing pressure, T_s_ = sintering temperature, DT = dwell time, PM = powder metallurgy, SSS = single-stage sintering, MW-TSS = microwave two-stage sintering, E_B_ = breakdown field, α = α_1_ = nonlinear exponent in the low-current region, I_L_ = leakage current, J_L_ = leakage current density.

**Table 6:** Dielectric properties ($\varepsilon_{APP}^{\prime }$ and tanδ) at 1 kHz of MOVs from the selected ZnO-0.5 mol.% V_2_O_5_ systems sintered in air between 825 °C and 950 °C for a dwell time of 3 h.

Content of MO Additives of ZnO-0.5% V_2_O_5_ Systems (mol.%)	MOV System Type	Sintering Temperature (t = 3 h)	Apparent Dielectric $\mathrm{Constant}\;(\varepsilon_{APP}^{\prime })$ at 1 kHz	Dissipation Factor (tanδ) at 1 kHz	Reference
0.5% Mn_3_O_4_	ZVM*	825 °C	1324.5	0.506	[124]
0.5% Mn_3_O_4_ + 0.025% Bi_2_O_3_	ZVM*B		528.3	0.225	
0.5% Mn_3_O_4_ + 0.05% Bi_2_O_3_			544.7	0.176	
0.5% Mn_3_O_4_ + 0.1% Bi_2_O_3_			734.4	0.212	
0.5% Mn_3_O_4_	ZVM*	900 °C	2438.3	0.424	[27]
0.5% Mn_3_O_4_ + 0.025% Yb_2_O_3_	ZVM*Y		2126.8	0.309	
0.5% Mn_3_O_4_ + 0.1% Yb_2_O_3_			1635.1	0.404	
0.5% Mn_3_O_4_ + 0.25% Yb_2_O_3_			1061.7	0.482	
0.5% Mn_3_O_4_ + 0.025% Er_2_O_3_	ZVM*E	850 °C 875 °C 900 °C 925 °C	1223.8 1626.9 2239.8 1489.3	0.543 0.403 0.332 0.273	[179]
0.5% Mn_3_O_4_	ZVM*	900 °C	2104.2	0.354	[184]
0.5% Mn_3_O_4_ + 0.05% Er_2_O_3_	ZVM*E		1845.3	0.297	
0.5% Mn_3_O_4_ + 0.1% Er_2_O_3_			1445.0	0.395	
0.5% Mn_3_O_4_ + 0.25% Er_2_O_3_			1314.1	0.468	
0.5% Mn_3_O_4_ + 0.05% Nb_2_O_5_	ZVM*N	875 °C 900 °C 925 °C 950 °C	779.2 1054.9 1631.8 1470.2	0.205 0.145 0.235 0.279	[50]
2% MnO_2_ + 0.1% Nb_2_O_5_	ZVMN	875 °C 900 °C 925 °C 950 °C	443.2 604.2 961.8 2062.6	0.281 0.228 0.134 0.287	[180]
2% MnO_2_ + 0.1% Nb_2_O_5_	ZVMN	900 °C	592.7	0.209	[26]
2% MnO_2_ + 0.1% Nb_2_O_5_ + 0.05% Yb_2_O_3_	ZVMNY		559.8	0.233	
2% MnO_2_ + 0.1% Nb_2_O_5_ + 0.1% Yb_2_O_3_			549.1	0.248	
2% MnO_2_ + 0.1% Nb_2_O_5_ + 0.25% Yb_2_O_3_			568.6	0.313	
2% MnO_2_ + 0.1% Nb_2_O_5_ + 0.05% Dy_2_O_3_	ZVMND	875 °C 900 °C 925 °C 950 °C	658.6 823.2 1644.3 2928.8	0.324 0.284 0.450 0.400	[181]
2% MnO_2_ + 0.1% Nb_2_O_5_	ZVMN	900 °C	822.0	0.261	[24]
2% MnO_2_ + 0.1% Nb_2_O_5_ + 0.05% Dy_2_O_3_	ZVMND		770.6	0.241	
2% MnO_2_ + 0.1% Nb_2_O_5_ + 0.1% Dy_2_O_3_			713.1	0.205	
2% MnO_2_ + 0.1% Nb_2_O_5_ + 0.25% Dy_2_O_3_			917.6	0.355	
2% MnO_2_ + 0.1% Nb_2_O_5_ + 0.05% Bi_2_O_3_ + 0.5% Co_3_O_4_ + 0.1% Dy_2_O_3_	ZVMNBCD	800 °C 875 °C 900 °C 925 °C	330.6 512.7 985.8 1391.4	0.211 0.203 0.206 0.238	[183]

**Table 7:** Dielectric properties ($\varepsilon_{APP}^{\prime }$ and tanδ) at 1 kHz of the ZnO-0.5 mol.% V_2_O_5_-2 mol.% MnO_2_ varistors before and after conducting DC-accelerated aging stress (0.85 × E_B_ at 85 °C for 24 h) [36].

Sintering Temperature (t = 3 h)	Apparent Dielectric Constant $\left(\varepsilon_{APP}^{\prime }\right)$ at 1 kHz			Dissipation Factor (tanδ) at 1 kHz		
	Initial	Stressed	$\Delta \varepsilon_{APP}^{\prime }$$/\varepsilon_{APP}^{\prime }$(%)	Initial	Stressed	Δtanδ/tanδ (%)
800 °C 850 °C 900 °C 950 °C	135.2 299.8 1163.5 800.9	~170 ~350 1160 ~880	25.7 16.7 −0.3 9.9	0.0781 0.1838 0.3160 0.2090	~1.125 ~0.610 0.385 ~0.338	1340.5 231.9 21.8 61.7

## Data Availability

Not applicable.

## Supplementary Materials

The following supporting information can be downloaded at: https://www.mdpi.com/article/10.3390/ma16103725/s1, Table S1: Processing parameters employing single-stage sintering (SSS) in air and main physical, structural, and electrical properties of MOVs from the selected ZnO-V_2_O_5_-based systems; Table S2: Processing parameters employing two-stage sintering (TSS) in air and main physical, structural and electrical properties of MOVs from the selected ZnO-V_2_O_5_-based systems.
