# Supplementary material for: Effects of Dopants and Processing Parameters on the Properties of ZnO-V2O5-Based Varistors Prepared by Powder Metallurgy: A Review
Source: Materials (Basel). 2023 May 14;16(10):3725. doi: 10.3390/ma16103725 (PMC10221863; doi:10.3390/ma16103725)
Supplement: Supplementary file 1 [file materials-16-03725-s001.zip › materials-2354847-supplementary.pdf]

materials

MDPI

Supplementary

Effects of Dopants and Processing Parameters on the Properties of ZnO-V_2O_5-Based Varistors Prepared by Powder Metallurgy: A Review

Magdalena Valentina Lungu *

Metallic, Composite and Polymeric Materials Department, National Institute for Research and Development in Electrical Engineering ICPE-CA, 030138 Bucharest, Romania
* Correspondence: magdalena.lungu@icpe-ca.ro (M.V.L); Tel.: +40-723-686-334

Table S1. Processing parameters employing single stage sintering (SSS) in air and main physical, structural and electrical properties of MOVs from the selected ZnO-V_2O_5-based systems.

Table S2. Processing parameters employing two stage sintering (TSS) in air and main physical, structural and electrical properties of MOVs from the selected ZnO-V_2O_5-based systems.

Table S1. Processing parameters employing single stage sintering (SSS) in air and main physical, structural and electrical properties of MOVs from the selected ZnO-V_2O_5-based systems.

Composition of MOV systems (mol. %)	Ball milling parameters [1] and preparation of MOV powders for PM processing	PM processing parameters [2]		Relative density (RD) of MOVs (% of TD)	Mean ZnO grain size (μm)	Crystalline phases		Breakdown field, E_B (kV/cm)	Non-linear exponent (α)	Leakage current density, J_L (mA/cm²)	Reference
		Pressing pressure; D × h of green compacts	Sintering temp. / Dwell time Heating rate / Cooling rate			Primary phase	Secondary phases				
99 % ZnO,0.5 % V_2O_5, 0.5 % Mn_3O_4	MD = 31 h (acetone); drying (120 °C, 12 h); mixing of MOV powders with 0.8 wt.% PVA; sieving (≤ 149 μm)	P_p = 9500 psi (65.5 MPa); Ø12 mm × 1.5 mm	825 °C / 3 h	~94.3	20.55	ZnO	$Zn_3(VO_4)_2$, ZnV_2O_4, VO_2 (the last two phases disappeared at 950°C)	2.110	14.91	0.304	[1]
			850 °C / 3 h	~94.0	21.61			1.710	6.71	0.328	
			875 °C / 3 h	~93.1	27.20			1.610	19.81	0.261	
			900 °C / 3 h	~92.5	23.84			1.536	13.97	0.290	
			950 °C / 3 h	~91.7	24.23			1.427	10.33	0.308	
99 % ZnO, 0.5 % V_2O_5, 0.5 % Mn_3O_4	MD = 24 h (acetone); GM = PP bottle and zirconia balls; drying (120 °C, 12 h); mixing of MOV powders with acetone + 0.8 wt.% PVB binder & sieving (≤ 149 μm)	P_p = 100 MPa; Ø10 mm × 1.5 mm	825 °C / 3 h	96.0	5.6	ZnO	$Zn_3(VO_4)_2$, ZnV_2O_4, VO_2	4.835	5.4	0.611	[2]
98.975 % ZnO, 0.5 % V_2O_5, 0.5 % Mn_3O_4, 0.025 % Bi_2O_3				95.3	4.4	ZnO	$Zn_3(VO_4)_2$, ZnV_2O_4, VO_2, $BiVO_4$	10.317	46.6	0.098	[2]
98.95 % ZnO, 0.5 % V_2O_5, 0.5 % Mn_3O_4, 0.05 % Bi_2O_3				95.0	3.9			6.027	31	0.043	[2]
98.9 % ZnO, 0.5 % V_2O_5, 0.5 % Mn_3O_4, 0.1 % Bi_2O_3				94.5	4.3			3.357	24.9	0.052	[2]
99 % ZnO, 0.5 % V_2O_5, 0.5 % Mn_3O_4		P_p = 80 MPa; Ø10 mm × 1.5 mm	900 °C / 3 h	95.3	5.2	ZnO	$Zn_3(VO_4)_2$	1.072	20	0.17	[3]
97.5 % ZnO, 0.5 % V_2O_5, 2 % Mn_3O_4				96.0	4.5	ZnO	$Zn_3(VO_4)_2$, Mn-rich	4.444	6	0.58	[3]
99 % ZnO, 0.5 % V_2O_5, 0.5 % MnO_2				94.6	8.4	ZnO	$Zn_3(VO_4)_2$	0.722	21	0.18	[3]
97.5 % ZnO, 0.5 % V_2O_5, 2 % MnO_2				95.3	5.2			0.999	27	0.042	[3]
98.95% ZnO, 0.5 % V_2O_5, 0.5 % Mn_3O_4, 0.05 % Nb_2O_5		P_p = 100 MPa; Ø10 mm × 1.5 mm	875 °C / 3 h	95.8	5.9	ZnO	$Zn_3(VO_4)_2$, ZnV_2O_4, VO_2	5.671	37.9	0.20	[4]
			900 °C / 3 h	95.2	6.9			3.967	47.0	0.14	
			925 °C / 3 h	94.5	9.3			2.489	24.2	0.32	
			950 °C / 3 h	93.4	14.3			1.443	17.8	0.09	
98.975 % ZnO, 0.5 % V_2O_5, 0.5 % Mn_3O_4, 0.025 % Er_2O_3		P_p = 100 MPa; Ø10 mm × 1.5 mm	850 °C / 3 h	95.5	6.1	ZnO	$Zn_3(VO_4)_2$, $ErVO_4$, ZnV_2O_4, VO_2	3.856	4.6	0.63	[5]
			875 °C / 3 h	95.2	6.4			1.385	17.9	0.24	
			900 °C / 3 h	94.6	7.2			0.922	21.3	0.13	
			925 °C / 3 h	93.9	8.7			2.352	30.0	0.20	

Table S1. *Cont.*

Composition of MOV systems (mol. %)	Ball milling parameters [1] and preparation of MOV powders for PM processing	PM processing parameters [2] Pressing pressure; D × h of green compacts	Sintering temp. / Dwell time Heating rate / Cooling rate	Relative density (RD) of MOVs (% of TD)	Mean ZnO grain size (μm)	Crystalline phases Primary phase	Secondary phases	Break-down field, E_B (kV/cm)	Non-linear exponent (α)	Leakage current density, J_L (mA/cm²)	Refer-ence
98.9 % ZnO, 0.5 % V_2O_5, 0.5 % Mn_3O_4, 0.05 % Nb_2O_5 0.05 % Er_2O_3	MD = 24 h (acetone); GM = PP bottle and zirconia balls; drying (120 °C, 12 h); mixing of MOV powders with acetone + 0.8 wt.% PVB binder; sieving (≤ 149 μm)	P_p = 98 MPa; Ø10 mm × 1.5 mm	875 °C / 3 h	98.9	6.3	ZnO	$Zn_3(VO_4)_2$, $ErVO_4$, ZnV_2O_4, VO_2	5.909	36.4	0.280	[6]
			900 °C / 3 h	98.2	8.4			4.175	45.6	0.240	
			925 °C / 3 h	97.8	13.1			1.995	14.5	0.390	
			950 °C / 3 h	97.3	16.6			1.028	17.8	0.400	
			HR=CR = 4°C/min								
99 % ZnO, 0.5 % V_2O_5, 0.5 % Mn_3O_4		P_p = 100 MPa; Ø10 mm × 1.5 mm	900 °C / 3 h polished MOVs (Ø8 mm × 1 mm)	93.3	7.2	ZnO	$Zn_3(VO_4)_2$, ZnV_2O_4, VO_2	0.922	20.7	0.31	[7]
98.975 % ZnO, 0.5 % V_2O_5, 0.5 % Mn_3O_4, 0.025 % Yb_2O_3				94.1	7.1	ZnO	$Zn_3(VO_4)_2$, $YbVO_4$, ZnV_2O_4, VO_2	1.025	29.2	0.13	[7]
98 % ZnO, 0.5 % V_2O_5, 0.5 % Mn_3O_4, 0.1 % Yb_2O_3				95.0	6.4			1.637	26.3	0.23	[7]
98.75 % ZnO, 0.5 % V_2O_5, 0.5 % Mn_3O_4, 0.25 % Yb_2O_3				96.0	5.9			3.774	5.7	0.60	[7]
99 % ZnO, 0.5 % V_2O_5, 0.5 % Mn_3O_4	MD = 24 h (acetone); GM = PP bottle and zirconia balls; drying; (120 °C, 12 h); mixing of MOV powders with acetone + 0.8 wt.% PVB binder; sieving (≤ 149 μm)	P_p = 100 MPa; Ø10 mm ×1.5 mm	900 °C / 3 h HR = CR = 4 °C/min	94.6	7.2	ZnO	$Zn_3(VO_4)_2$, ZnV_2O_4, VO_2	1.016	22.0	0.21	[8]
98.95 % ZnO, 0.5 % V_2O_5, 0.5 % Mn_3O_4, 0.05 % Er_2O_3				94.8	6.9	ZnO	$Zn_3(VO_4)_2$, $ErVO_4$, ZnV_2O_4, VO_2	1.162	24.2	0.11	[8]
98.9 % ZnO, 0.5 % V_2O_5, 0.5 % Mn_3O_4, 0.1 % Er_2O_3				95.2	6.2			1.806	30.0	0.20	[8]
98.75 % ZnO, 0.5 % V_2O_5, 0.5 % Mn_3O_4, 0.25 % Er_2O_3				95.5	6.0			3.185	4.8	0.60	[8]
97.5 % ZnO, 0.5 % V_2O_5, 2 % MnO_2		Pp = 80 MPa; Ø10 mm × 1.5 mm	800 °C / 3 h	96.2	2.1	ZnO	$Zn_3(VO_4)_2$	17.640	38.1	0.14	[9]
			850 °C / 3 h	96.7	4.4			7.881	17.0	0.27	
			900 °C / 3 h	95.3	5.2			0.992	27.2	0.17	
			950 °C / 3 h	94.1	10.1			2.430	32.0	0.11	

Table S1. *Cont.*

Composition of MOV systems (mol. %)	Ball milling parameters [1] and preparation of MOV powders for PM processing	PM processing parameters [2]		Relative density (RD) of MOVs (% of TD)	Mean ZnO grain size (μm)	Crystalline phases		Break-down field, E_B (kV/cm)	Non-linear exponent (α)	Leakage current density, J_L (mA/cm²)	Refer-ence
		Pressing pressure; D × h of green compacts	Sintering temp. / Dwell time Heating rate / Cooling rate			Pri-mary phase	Secondary phases				
98.4 % ZnO, 0.5 % V_2O_5, 1 % Nb_2O_5, 0.1 % In_2O_3	RS = 300 rpm; MD = 35 h (dry); BPR = 16:1; GM = agate vial and agate balls	P_p = 500 MPa; Ø10 mm ×1.5 mm	850 °C / 1 h	98.8	1.0 ± 0.1	ZnO	$Zn_3(VO_4)_2$, $Zn_2V_2O_7$, $Zn(VO_3)_2$, $InVO_4$, $Zn_7I_2O_{10}$	11.8 ± 0.2	102 ± 8	0.090 ± 0.003	[10]
			875 °C / 1 h	~ 99.4	1.1 ± 0.1			14.2 ± 0.1	153 ± 7	0.062 ± 0.002	
			900 °C / 1 h	~ 99.8	1.6 ± 0.1			11.6 ± 0.2	132 ± 8	0.083 ± 0.001	
			925 °C / 1 h	~ 99.2	2.0 ± 0.1			8.6 ± 0.3	82 ± 6	0.118 ± 0.005	
			HR = CR = 5 °C/min								
99.4 % ZnO, 0.5 % V_2O_5, 0.1 % Nb_2O_5		Pp = 500 MPa; Ø10 mm × 3-4 mm	850 °C / 1 h	~ 98.40	2.83	ZnO	$Zn_3(VO_4)_2$, ZnV_2O_4	4.258	24	0.294	[11]
			875 °C / 1 h	~ 98.75	3.12			3.642	15	0.284	
			900 °C / 1 h	~ 98.90	3.87			3.158	12	0.337	
			925°C / 1 h	~ 98.90	4.60			3.375	13	0.256	
			950 °C / 1 h	~ 98.60	5.81			2.624	11	0.246	
			975 °C / 1 h	~ 97.30	7.17			1.440	9	0.204	
97.4 % ZnO, 0.5 % V_2O_5, 2 % MnO_2, 0.1 % Nb_2O_5	RS = 300 rpm; MD = 35 h (dry); BPR = 16:1; GM = agate vial and agate balls	Pp = 500 MPa; Ø10 mm × 1.5 mm	900 °C / 1 h	97.5	3.2	ZnO	$Zn_3(VO_4)_2$, $Zn_4V_2O_9$, V_2O_5, Mn-rich	3.566	~ 16.3	–	[12]
			1100 °C / 1 h	99.4	10.8			1.588	~ 16.4		
			1300 °C / 1 h	98.4	37.6			0.829	~ 14.0		
			HR= CR= 5 °C/min								
96.9 % ZnO, 0.5 % V_2O_5, 2 % MnO_2, 0.1 % Nb_2O_5, 0.5 % Er_2O_3		P_p = 500 MPa; Ø10 mm × 1.5 mm	900 °C / 1 h	80.8	0.5	ZnO	$Zn_3(VO_4)_2$, $Zn_4V_2O_9$, V_2O_5, $ErVO_4$, Mn-rich, Er-rich	–	–	–	[12]
			1100 °C / 1 h	97.4	7.2			2.585	26.0		
			1300 °C / 1 h	98.0	19.6			~ 1.1	~ 17.5		
			HR=CR=5 °C/min								
96.4 % ZnO,0.5 % V_2O_5, 2 % MnO_2,0.1 % Nb_2O_5, 1 % Er_2O_3	RS = 300 rpm; MD = 35 h (dry); BPR = 16:1; GM = agate vial and agate balls	P_p = 500 MPa; Ø10 mm ×1.5 mm	900 °C / 1 h	73.9	0.3	ZnO	$Zn_3(VO_4)_2$, $Zn_4V_2O_9$, V_2O_5, $ErVO_4$, Mn-rich, Er-rich	–	–	–	[12]
			1100 °C / 1 h	93.7	0.9			–	–		
			1300 °C / 1 h	96.3	3.6			~ 0.5	~ 3		
			HR=CR = 5 °C/min								
96.9 % ZnO,0.5 % V_2O_5, 2 % MnO_2, 0.1 % Nb_2O_5, 0.5 % Er_2O_3		P_p = 500 MPa; Ø10 mm ×3-4 mm	950 °C / 1 h	95.0	1.8	ZnO	$Zn_3(VO_4)_2$, $Zn_4V_2O_9$, $ErVO_4$, Mn-rich, Er-rich	10.286	150	0.180	[13]
			1000 °C / 1 h	~ 96.7	~ 3.2			~ 5.3	~ 54	~ 0.347	
			1050 °C / 1 h	~ 97.8	~ 4.2			~ 4.8	~ 32	~ 0.290	
			1100 °C / 1 h	98.0	7.2			2.584	26	~ 0.288	
			HR = 5 °C/min								

Table S1. *Cont.*

Composition of MOV systems (mol. %)	Ball milling parameters [1] and preparation of MOV powders for PM processing	PM processing parameters [2]		Relative density (RD) of MOVs (% of TD)	Mean ZnO grain size (μm)	Crystalline phases		Break-down field, E_B (kV/cm)	Non-linear exponent (α)	Leakage current density, J_L (mA/cm^2)	Refer-ence
		Pressing pressure; D × h of green compacts	Sintering temp. / Dwell time Heating rate / Cooling rate			Primary phase	Secondary phases				
97.4 % ZnO, 0.5 % V_2O_5, 2 % MnO_2, 0.1 % Nb_2O_5	RS = 300 rpm; MD = 35 h (dry); BPR = 16:1; GM = agate vial and agate balls	P_P = 500 MPa; Ø10 mm × 1.5 mm	1050 °C / 1 h HR= 15 °C/min	98.2 ± 0.2	9.3 ± 0.2	ZnO	$Zn_3(VO_4)_2$, $Zn_4V_2O_9$, V_2O_5, Mn-rich	1.8 ± 0.1	12 ± 1	0.45 ± 0.03	[12,14]
96.9 % ZnO, 0.5 % V_2O_5, 2 % MnO_2, 0.1 % Nb_2O_5, 0.5 % Er_2O_3				97.9 ± 0.2	5.5 ± 0.2	ZnO	$Zn_3(VO_4)_2$, $Zn_4V_2O_9$, V_2O_5, $ErVO_4$, Mn-rich, Er-rich	4.8 ± 0.1	32 ± 2	0.29 ± 0.03	[12,14]
97.4 % ZnO, 0.5 % V_2O_5, 2 % MnO_2, 0.1 % Nb_2O_5	MD = 24 h (acetone); GM = PP bottle and zirconia balls; drying (120 °C, 12 h), mixing of MOV powders with acetone + 0.8 wt.% PVB binder, drying; sieving (≤ 149 μm)	P_P = 100 MPa; Ø10 mm × 1.5 mm	875 °C / 3 h<br>900 °C / 3 h<br>925 °C / 3 h<br>950 °C / 3 h	95.5<br>95.3<br>94.5<br>94.1	4.4<br>5.5<br>7.2<br>9.6	ZnO	$Zn_3(VO_4)_2$, ZnV_2O_4, VO_2	6.991<br>4.800<br>2.241<br>0.943	44<br>50<br>38<br>25	0.2013<br>0.0949<br>0.0258<br>0.1737	[15]
97.35 % ZnO, 0.5 % V_2O_5, 2 % MnO_2, 0.1 % Nb_2O_5, 0.05 % Gd_2O_3		P_P = 100 MPa; Ø10 mm × 1.5 mm	875 °C / 3 h<br>900 °C / 3 h<br>925 °C / 3 h<br>950 °C / 3 h	95.8<br>95.0<br>94.6<br>93.8	4.1<br>5.3<br>8.1<br>11.7	ZnO	$Zn_3(VO_4)_2$, ZnV_2O_4, $GdVO_4$, VO_2	7.138<br>5.365<br>3.024<br>0.920	50.9<br>66.1<br>26.6<br>19.0	0.135<br>0.077<br>0.270<br>0.119	[16]
97.4 % ZnO, 0.5 % V_2O_5, 2 % MnO_2, 0.1 % Nb_2O_5		P_P = 100 MPa; Ø10 mm × 1.5 mm	875 °C / 3 h HR = CR = 4°C/min polished MOVs (Ø8 mm × 1 mm)	95.5	4.4	ZnO	$Zn_3(VO_4)_2$, ZnV_2O_4, V_2O_5, Mn-rich	6.991	44	0.201	[17]
97.35 % ZnO, 0.5 % V_2O_5, 2 % MnO_2, 0.1 % Nb_2O_5, 0.05 % Er_2O_3				96.2	4.3	ZnO	$Zn_3(VO_4)_2$, ZnV_2O_4, $ErVO_4$, V_2O_5, Mn-rich	7.095	50	0.094	[17]
97.3 % ZnO, 0.5 % V_2O_5, 2 % MnO_2, 0.1 % Nb_2O_5, 0.1 % Er_2O_3				96.4	4.2			7.408	55	0.128	[17]
97.15 % ZnO, 0.5 % V_2O_5, 2 % MnO_2, 0.1 % Nb_2O_5, 0.25 % Er_2O_3				96.7	4.6			6.108	14	0.384	[17]

Table S1. *Cont.*

Composition of MOV systems (mol. %)	Ball milling parameters [1] and preparation of MOV powders for PM processing	PM processing parameters [2]		Relative density (RD) of MOVs (% of TD)	Mean ZnO grain size (µm)	Crystalline phases		Break-down field, E_B (kV/cm)	Non-linear exponent (α)	Leakage current density, J_L (mA/cm²)	Refer-ence
		Pressing pressure; D × h of green compacts	Sintering temp. / Dwell time Heating rate / Cooling rate			Primary phase	Secondary phases				
99.5 % ZnO, 0.5 % V_2O_5	MD = 8 h; mixing of MOV powders with 2 % ethyl cellulose binder and ball milling for 1 h; sieving (≤ 149 µm)	P_p = 200 MPa; Ø20 mm × h (unspecified height)	1100 °C / 2 h	58-62 (green discs); unspeci-fied RD for sintered discs	17.1	ZnO	β-$Zn_3(VO_4)_2$, γ-$Zn_3(VO_4)_2$	0.943	3.1	–	[18]
98.5% ZnO, 0.5 % V_2O_5, 1 % MnO_2			1100 °C / 2 h		14.2	ZnO	β-$Zn_3(VO_4)_2$, γ-$Zn_3(VO_4)_2$	1.061	4.2	0.3749	[18]
99.0 % ZnO, 0.5 % V_2O_5 0.5 % PbO			1100 °C / 2 h		13.7	ZnO	β-$Zn_3(VO_4)_2$, γ-$Zn_3(VO_4)_2$	1.763	2.8	–	[18]
94.5% ZnO, 0.5 % V_2O_5, 1 % MnO_2, 2.3 % PbO, 1.7 % B_2O_3			1100 °C / 2 h		16.1	ZnO	β-$Zn_3(VO_4)_2$, γ-$Zn_3(VO_4)_2$, $Pb_{10}B_3O_{17}$	2.394	35.3	0.00272	[18]
99.5 % ZnO, 0.5 % V_2O_5	MD = 24 h (acetone); GM = PP bottle and zirconia balls; filtering; drying; calcination (650°C, 3 h); mixing of MOV powders with PVB binder; sieving (≤ 149 µm)	F_p = 90 kN unspecified Ø × h	900 °C / 3 h HR = CR = 3 °C/min annealing of MOVs discs (700 °C, 5 h)	96.84	8.4	ZnO	α-$Zn_3(VO_4)_2$, β-$Zn_3(VO_4)_2$, ZnV_2O_4	0.837	2.9	0.581	[19]
99.45 % ZnO, 0.5 % V_2O_5, 0.05 % Nb_2O_5				96.17	5.8	ZnO	α-$Zn_3(VO_4)_2$, γ-$Zn_3(VO_4)_2$, ZnV_2O_4	2.291	4.9	0.461	[19]
99.4 % ZnO, 0.5 % V_2O_5, 0.1 % Nb_2O_5				95.90	7.0	ZnO	α-$Zn_3(VO_4)_2$, β-$Zn_3(VO_4)_2$, γ-$Zn_3(VO_4)_2$, ZnV_2O_4	2.815	7.1	0.311	[19]
99.25 % ZnO, 0.5 % V_2O_5, 0.25 % Nb_2O_5				95.48	7.3			1.953	3.7	0.622	[19]
99 % ZnO, 0.5 % V_2O_5, 0.5 % Nb_2O_5				94.70	11.0			1.566	2.3	0.749	[19]
97.4 % ZnO, 0.5 % V_2O_5, 0.1 Nb_2O_5, 2 % $MnCO_3$				96.06	9.2	ZnO	α-$Zn_3(VO_4)_2$, γ-$Zn_3(VO_4)_2$, ZnV_2O_4	4.985	24.3	0.063	[19]
97.4 % ZnO, 0.5 % V_2O_5, 2 % MnO_2, 0.1 % Nb_2O_5	mixing, drying, and sieving of MOV powders (≤ 149 µm)	P_p = 100 MPa; Ø10 mm × 1.5 mm	900 °C / 3 h polished MOVs (Ø8 mm × 1 mm)	~95.3	5.6	ZnO	$Zn_3(VO_4)_2$, ZnV_2O_4, VO_2	4.874	51	0.0568	[20]

Table S1. *Cont.*

Composition of MOV systems (mol. %)	Ball milling parameters [1] and preparation of MOV powders for PM processing	PM processing parameters [2]		Relative density (RD) of MOVs (% of TD)	Mean ZnO grain size (μm)	Crystalline phases		Break-down field, E_B (kV/cm)	Non-linear exponent (α)	Leakage current density, J_L (mA/cm^2)	Refer-ence
		Pressing pressure; D × h of green compacts	Sintering temp. / Dwell time Heating rate / Cooling rate			Primary phase	Secondary phases				
97.375 % ZnO, 0.5 % V_2O_5, 2 % MnO_2, 0.1 % Nb_2O_5, 0.025 % Bi_2O_3	mixing, drying, and sieving of MOV powders (≤ 149 μm)	P_p = 100 MPa; Ø10 mm × 1.5 mm	900 °C / 3 h polishing of MOV discs to Ø8 mm × 1 mm	~93.9	5.9	ZnO	$Zn_3(VO_4)_2$, ZnV_2O_4, VO_2, $BiVO_4$	4.355	60	0.0200	[20]
97.35 % ZnO, 0.5 % V_2O_5, 2 % MnO_2, 0.1 % Nb_2O_5, 0.05 % Bi_2O_3				~92.9	6.3			3.917	51	0.0419	[20]
97.3 % ZnO, 0.5 % V_2O_5, 2 % MnO_2, 0.1 % Nb_2O_5, 0.1 % Bi_2O_3				~93.3	6.9			2.972	39	0.0554	[20]
97.15 % ZnO, 0.5 % V_2O_5, 2 % MnO_2, 0.1 % Nb_2O_5, 0.25 % Bi_2O_3				~93.4	7.2			2.205	31	0.0453	[20]
96.75 % ZnO, 0.5 % V_2O_5, 2 % MnO_2, 0.1 % Nb_2O_5, 0.5 % Co_3O_4, 0.1 % Dy_2O_3, 0.05 % Bi_2O_3	MD = 24 h (acetone); GM = PP bottle and zirconia balls; drying; mixing of MOV powders with acetone + 0.8 wt.% PVB; sieving (≤ 149 μm)	P_p = 1000 kg/cm^2 (98 MPa); Ø10 mm × 1.5 mm binder burn out at 600 °C for 1 h	850 °C / 3 h	95.7	4.4	ZnO	$Zn_3(VO_4)_2$, ZnV_2O_4, $DyVO_4$, an unidentified phase	8.016	44	0.0390	[21]
			875 °C / 3 h	95.2	5.7			4.522	59	0.0362	
			900 °C / 3 h	94.3	7.5			2.351	45	0.0600	
			925 °C / 3 h	93.4	11.8			1.715	38	0.1006	
98.25 % ZnO, 0.5 % V_2O_5, 0.5 % Mn_3O_4, 0.1 % Nb_2O_5, 0.5 % Co_3O_4, 0.1 % Dy_2O_3, 0.05 % Bi_2O_3			850 °C / 3 h	95.8	4.6			5.919	39.1	0.0357	[22]
			875 °C / 3 h	94.8	6.1			3.370	43.6	0.0282	
			900 °C / 3 h	94.1	7.2			1.871	37.2	0.0630	
			925 °C / 3 h	93.8	8.7			1.465	33.3	0.0741	
97.4 % ZnO, 0.5 % V_2O_5, 2 % MnO_2, 0.1 % Nb_2O_5	MD = 24 h (acetone); GM = PP bottle and zirconia balls; drying (120 °C, 12 h); mixing of MOV pow-ders with acetone + 0.8 wt.% PVB binder; sieving (≤ 149 μm)	P_p = 100 MPa; Ø10 mm × 1.5 mm	900°C / 3 h HR = CR = 4°C/min lapping and polishing of MOV discs (Ø8 mm × 1 mm)	96.0	5.5	ZnO	$Zn_3(VO_4)_2$, ZnV_2O_4, VO_2	4.552	35.8	0.30	[23]
97.35 % ZnO, 0.5 % V_2O_5, 2 % MnO_2, 0.1 % Nb_2O_5, 0.05 % Dy_2O_3				95.3	5.4	ZnO	$Zn_3(VO_4)_2$, ZnV_2O_4, VO_2, $DyVO_4$	4.789	38.4	0.25	[23]

Table S1. *Cont.*

Composition of MOV systems (mol. %)	Ball milling parameters [1] and preparation of MOV powders for PM processing	PM processing parameters [2]: Pressing pressure; D × h of green compacts	PM processing parameters [2]: Sintering temp. / Dwell time Heating rate / Cooling rate	Relative density (RD) of MOVs (% of TD)	Mean ZnO grain size (μm)	Crystalline phases: Primary phase	Crystalline phases: Secondary phases	Break-down field, E_B (kV/cm)	Non-linear exponent (α)	Leakage current density, J_L (mA/cm²)	Refer-ence
97.3 % ZnO, 0.5 % V_2O_5, 2 % MnO_2, 0.1 % Nb_2O_5, 0.1 % Dy_2O_3	MD = 24 h (acetone); GM = PP bottle and zirconia balls; drying (120 °C, 12 h); mixing of MOV powders with acetone + 0.8 wt.% PVB binder; sieving (≤ 149 μm)	P_p = 100 MPa; Ø10 mm × 1.5 mm	900°C / 3 h HR = CR = 4°C/min lapping and polishing of MOV discs (Ø8 mm × 1 mm)	95.7	5.2	ZnO	$Zn_3(VO_4)_2$, ZnV_2O_4, VO_2, $DyVO_4$	5.117	53.3	0.21	[23]
97.15 % ZnO, 0.5 % V_2O_5, 2 % MnO_2, 0.1 % Nb_2O_5, 0.25 % Dy_2O_3				96.5	5.9			4.095	11.2	0.42	[23]
97.35 % ZnO, 0.5 % V_2O_5, 2 % MnO_2, 0.1 % Nb_2O_5, 0.05 % Dy_2O_3		P_p = 100 MPa; Ø10 mm × 1.3 mm	875 °C / 3 h<br>900 °C / 3 h<br>925 °C / 3 h<br>950 °C / 3 h	96.4<br>95.7<br>95.0<br>94.3	4.3<br>5.4<br>8.2<br>10.9	ZnO	$Zn_3(VO_4)_2$, ZnV_2O_4, VO_2, $DyVO_4$	6.095<br>4.927<br>1.972<br>0.996	27.6<br>39.2<br>9.9<br>14.6	0.32<br>0.24<br>0.49<br>0.36	[24]
97.4 % ZnO, 0.5 % V_2O_5, 2 % MnO_2, 0.1 % Nb_2O_5		P_p = 100 MPa; Ø10 mm × 1.5 mm	900 °C / 3 h HR = CR = 4 °C/min lapping and polishingof MOV discs (Ø8 mm × 1 mm)	94.1	5.6	ZnO	$Zn_3(VO_4)_2$, ZnV_2O_4, VO_2	4.818	47.3	0.0910	[24]
97.375 % ZnO, 0.5 % V_2O_5, 2 % MnO_2, 0.1 % Nb_2O_5, 0.025 % Tb_4O_7				95.0	5.4	ZnO	$Zn_3(VO_4)_2$, ZnV_2O_4, VO_2, $TbVO_4$	5.024	65.5	0.0721	[24]
97.35 % ZnO, 0.5 % V_2O_5, 2 % MnO_2, 0.1 % Nb_2O_5, 0.05 % Tb_4O_7				95.7	5.2			5.272	47.4	0.1525	[24]
97.3 % ZnO, 0.5 % V_2O_5, 2 % MnO_2, 0.1 % Nb_2O_5, 0.1 % Tb_4O_7				96.0	5.8			4.934	39.8	0.1722	[24]
97.4 % ZnO, 0.5 % V_2O_5, 2 % MnO_2, 0.1 % Nb_2O_5	MD = 35 h (dry); BPR = 16:1	Pp = 500 MPa; Ø10 mm × 3-4 mm	1100 °C / 0.5 h<br>1100 °C / 2 h<br>1100 °C / 4 h<br>1100 °C / 8 h<br>HR = CR = 5 °C/min	97-99	10.7<br>~ 16.1<br>~ 24.3<br>32.6	ZnO	$Zn_3(VO_4)_2$, $Zn_4V_2O_9$, V_2O_5, Zn_2MnO_4, Mn-rich	1.976<br>~ 1.250<br>~ 1.000<br>~ 0.810	17.5<br>~ 15<br>~ 13<br>~ 8	–	[25]

Table S1. *Cont.*

Composition of MOV systems (mol. %)	Ball milling parameters [1] and preparation of MOV powders for PM processing	PM processing parameters [2]		Relative density (RD) of MOVs (% of TD)	Mean ZnO grain size (μm)	Crystalline phases		Break-down field, E_B (kV/cm)	Non-linear exponent (α)	Leakage current density, J_L (mA/cm²)	Refer-ence
		Pressing pressure; D × h of green compacts	Sintering temp. / Dwell time Heating rate / Cooling rate			Primary phase	Secondary phases				
96.9 % ZnO, 0.5 % V_2O_5, 2 % MnO_2, 0.1 % Nb_2O_5, 0.5 % Er_2O_3	MD = 35 h (dry); BPR = 16:1	Pp = 500 MPa; Ø10 mm × 3-4 mm	1100 °C / 0.5 h<br>1100 °C / 2 h<br>1100 °C / 4 h<br>1100 °C / 8 h<br>HR= CR = 5 °C/min	97-99	6.3<br>~ 7.8<br>~ 9.6<br>10.8	ZnO	$Zn_3(VO_4)_2$, $Zn_4V_2O_9$, Zn_2MnO_4, V_2O_5, $ErVO_4$, Mn-rich, Er-rich	3.880<br>~ 3.060<br>~ 2.530<br>~ 1.810	27<br>~ 17<br>~ 16<br>~ 12.5	–	[25]
97.4 % ZnO, 0.5 % V_2O_5, 2 % $MnCO_3$, 0.1 % Nb_2O_5	MD = 12 h (ethanol); GM = PP bottle and zirconia balls; mixing of dried MOV powders with 5 wt.% PVA binder; sieving (≤ 149 μm)	P_p = 100 MPa; Ø12 mm × 1.3 mm; binder burnout at 500 °C for 1 h	930 °C / 3 h<br>HR = 4 °C/min	95.1	6.38 ± 0.01	ZnO	$Zn_3(VO_4)_2$, $ZnMn_2O_4$, ZnV_2O_4	2.960	33.6	0.042	[26]
97.35 % ZnO, 0.5 % V_2O_5, 2 % $MnCO_3$, 0.1 % Nb_2O_5, 0.05 % Y_2O_3				96.4	6.40 ± 0.01	ZnO	$Zn_3(VO_4)_2$, $ZnMn_2O_4$, ZnV_2O_4, YVO_4	2.970	36.5	0.042	[26]
97.35 % ZnO, 0.5 % V_2O_5, 2 % $MnCO_3$, 0.1 % Nb_2O_5, 0.05 % Nd_2O_3				96.7	6.38 ± 0.01	ZnO	$Zn_3(VO_4)_2$, $ZnMn_2O_4$, ZnV_2O_4, $NdVO_4$	3.010	25.9	0.188	[26]
97.35 % ZnO, 0.5 % V_2O_5, 2 % $MnCO_3$, 0.1 % Nb_2O_5, 0.05 % Sm_2O_3				95.5	6.40 ± 0.01	ZnO	$Zn_3(VO_4)_2$, $ZnMn_2O_4$, ZnV_2O_4, $SmVO_4$	2.760	36.4	0.039	[26]
97.4 % ZnO, 0.5 % V_2O_5, 2 % $MnCO_3$, 0.1 % Nb_2O_5	MD = 12 h (ethanol); GM = PP bottle and zirconia balls; drying; mixing of MOV powders with 5 wt.% PVA binder and sieving (≤ 149 μm)	P_p = 100 MPa; Ø12 mm × 1.3 mm	875 °C / 2 h<br>875 °C / 3 h<br>875 °C / 4 h<br>875 °C / 5 h<br>850 °C / 3 h<br>900 °C / 3 h<br>925 °C / 3 h<br>HR = 4 °C/min	~92.65<br>~92.65<br>~92.70<br>~92.70<br>~92.40<br>~92.50<br>~96.60	4.3<br>5.6<br>6.2<br>6.4<br>4.3<br>6.4<br>7.4	ZnO	$Zn_3(VO_4)_2$, ZnV_2O_4, $ZnMn_2O_4$	6.370<br>5.260<br>4.950<br>4.420<br>7.560<br>3.320<br>3.100	32.3<br>37.8<br>35.7<br>31.0<br>36.0<br>37.5<br>20.4	0.174<br>0.187<br>0.185<br>0.292<br>0.162<br>0.115<br>0.347	[27]

Table S1. *Cont.*

Composition of MOV systems (mol. %)	Ball milling parameters [1] and preparation of MOV powders for PM processing	PM processing parameters [2]		Relative density (RD) of MOVs (% of TD)	Mean ZnO grain size (μm)	Crystalline phases		Break-down field, E_B (kV/cm)	Non-linear exponent (α)	Leakage current density, J_L (mA/cm²)	Refer-ence
		Pressing pressure; D × h of green compacts	Sintering temp. / Dwell time Heating rate / Cooling rate			Primary phase	Secondary phases				
97.4 % ZnO, 0.5 % V_2O_5, 2 % $MnCO_3$, 0.1 % Nb_2O_5, 0.1 % Ce–La	MD = 12 h (ethanol); GM = PP bottle and zirconia balls; drying; mixing of MOV powders with 5 wt.% PVA binder and sieving (≤ 149 μm)	P_P = 100 MPa; Ø12 mm × 1.3 mm	875 °C / 2 h<br>875 °C / 3 h<br>875 °C / 4 h<br>875 °C / 5 h<br>850 °C / 3 h<br>900 °C / 3 h<br>925 °C / 3 h<br>HR = 4 °C/min	~92.90<br>~93.00<br>~92.80<br>~92.60<br>~92.50<br>~97.60<br>~97.50	3.8<br>5.1<br>5.2<br>5.2<br>3.8<br>5.2<br>7.3	ZnO	$Zn_3(VO_4)_2$, ZnV_2O_4, $ZnMn_2O_4$, Ce(La)VO_4	7.420<br>5.540<br>5.430<br>4.820<br>8.580<br>3.630<br>3.130	37.5<br>48.4<br>37.9<br>32.0<br>37.0<br>45.0<br>26.0	0.180<br>0.120<br>0.131<br>0.273<br>0.096<br>0.118<br>0.277	[27]
99.4 % ZnO, 0.5 % V_2O_5, 0.1 % Nb_2O_5	MD = 24 h (acetone); GM = PP bottle and zirconia balls; filtering, drying, calcination (650 °C, 3 h); mixing of MOV powders with PVB binder and sieving (≤ 149 μm)	P_P = 150 MPa; Ø12.5 mm × 1.25 mm	950 °C / 3 h HR = CR = 3 °C/min	95.76	10.11	ZnO	α-$Zn_3(VO_4)_2$, β-$Zn_3(VO_4)_2$, ZnV_2O_4, $Zn_3Nb_2O_8$	1.2874	4.2	0.386	[28]
97.9 % ZnO, 0.5 % V_2O_5, 0.1 % Nb_2O_5, 1.5 % $MnCO_3$				96.26	16.16	ZnO	α-$Zn_3(VO_4)_2$, γ-$Zn_3(VO_4)_2$, ZnV_2O_4, $MnZn_2Nb_2O_8$	1.2145	11.6	0.125	[28]
97.4 % ZnO, 0.5 % V_2O_5, 0.1 % Nb_2O_5, 2 % $MnCO_3$				95.97	16.78			1.6556	18.9	0.130	[28]
96.9 % ZnO, 0.5 % V_2O_5, 0.1 % Nb_2O_5, 2.5 % $MnCO_3$				96.81	15.87			1.9147	21.6	0.036	[28]
96.4 % ZnO, 0.5 % V_2O_5, 0.1 % Nb_2O_5, 3 % $MnCO_3$				96.70	18.53			1.3699	9.9	0.359	[28]
97.4 % ZnO, 0.5 % V_2O_5, 2 % MnO_2, 0.1 % Nb_2O_5	MD = 35 h (dry); GM = agate vial and balls; BPR = 16:1	P_P = 500 MPa; Ø10 mm ×3-4 mm	950 °C / 1 h<br>1100 °C / 0.5 h<br>1100 °C / 1 h<br>1250 °C / 1 h	~97<br>~99.8<br>~99.5<br>~98	~ 7<br>~10.8<br>11.3<br>~36	ZnO	$Zn_3(VO_4)_2$, $Zn_4V_2O_9$, Zn_2MnO_4, V_2O_5, Mn-rich	–<br>–<br>1.588<br>–	–	–	[29]
96.9 % ZnO, 0.5 % V_2O_5, 2 % MnO_2, 0.1 % Nb_2O_5, 0.5 % Er_2O_3			950 °C / 1 h<br>1100 °C / 0.5 h<br>1100 °C / 1 h<br>HR = CR = 5 °C/min	–<br>~99.2<br>~98.5	–<br>~6.3<br>~ 7.2	ZnO	$Zn_3(VO_4)_2$, $Zn_4V_2O_9$, Zn_2MnO_4, V_2O_5, $ErVO_4$, Mn-rich, Er-rich	–<br>3.880<br>2.584	–	–	[29]

Table S1. *Cont.*

Composition of MOV systems (mol. %)	Ball milling parameters [1] and preparation of MOV powders for PM processing	PM processing parameters [2]: Pressing pressure; D × h of green compacts	PM processing parameters [2]: Sintering temp. / Dwell time Heating rate / Cooling rate	Relative density (RD) of MOVs (% of TD)	Mean ZnO grain size (μm)	Crystalline phases: Primary phase	Crystalline phases: Secondary phases	Break-down field, E_B (kV/cm)	Non-linear exponent (α)	Leakage current density, J_L (mA/cm²)	Refer-ence
96.4 % ZnO, 0.5 % V_2O_5, 2 % MnO_2, 0.1 % Nb_2O_5,1 % Er_2O_3	MD = 35 h (dry); GM = agate vial and balls; BPR = 16:1	P_P = 500 MPa; Ø10 mm × 3-4 mm	1100 °C / 0.5 h HR=CR=5 °C/min	~90.5	~0.90	ZnO	$Zn_3(VO_4)_2$, $Zn_4V_2O_9$, Zn_2MnO_4, V_2O_5, $ErVO_4$, Mn-rich, Er-rich	–	–	–	[29]
95.4 % ZnO, 0.5 % V_2O_5, 2 % MnO_2, 0.1 % Nb_2O_5,2 % Er_2O_3			950 °C / 1 h<br>1100 °C / 0.5 h<br>1100 °C / 1 h<br>1250 °C / 1 h	72<br>~84.8<br>~85<br>~93	0.75<br>~0.82<br>0.82<br>~2.10	ZnO		–	–	–	[29]
98.65 % ZnO, 1 % V_2O_5, 0.35 % Cr_2O_3	MD = 24 h (absolute alcohol); GM = PP bottle and zirconia balls; drying (80 °C, 24 h); mixing of MOV powders with 5 wt.% PVA binder; sieving (≤ 149 μm)	P_P = 130 MPa; Ø12 mm × 1 mm binder burnout (500 °C, 1 h)	875 °C / 4 h HR = 4°C/min	95.1	7.19	ZnO	α-$Zn_3(VO_4)_2$, $Zn_4V_2O_9$, $ZnCr_2O_4$,	3.649	7.2	–	[30]
98.4 % ZnO, 1 % V_2O_5, 0.35 % Cr_2O_3, 0.25 % $PrMnO_3$				94.8	5.94	ZnO	α-$Zn_3(VO_4)_2$, $Zn_4V_2O_9$, $ZnCr_2O_4$, $PrVO_4$	3.632	7.7	–	[30]
98.15 % ZnO, 1 % V_2O_5, 0.35 % Cr_2O_3, 0.5 % $PrMnO_3$				95.1	5.81			3.726	7.8	–	[30]
97.9 % ZnO, 1 % V_2O_5, 0.35 % Cr_2O_3, 0.75 % $PrMnO_3$				95.7	5.80			4.620	8.9	–	[30]
99.75 % ZnO,0.25 % V_2O_5	MD = 24 h (deionized water); GM = zirconia balls; drying; sieving (≤ 149 μm)	unspecified P_P and Ø × h	900 °C / 4 h	–	7.7	ZnO	β-$Zn_3(VO_4)_2$	1.050	5.0	0.50	[31]
98.75 % ZnO, 0.25 % V_2O_5, 1 % MnO_2				–	5.4	ZnO	γ-$Zn_3(VO_4)_2$	1.100	16.7	0.08	[31]
98.5 % ZnO, 0.5 % V_2O_5, 1 % MnO_2				–	4.6	ZnO	γ-$Zn_3(VO_4)_2$	1.350	18.6	0.02	[31]
98 % ZnO, 1 % V_2O_5, 1 % MnO_2				–	6.5	ZnO	β-$Zn_3(VO_4)_2$, γ-$Zn_3(VO_4)_2$	0.600	12.3	0.14	[31]
97 % ZnO, 2 % V_2O_5, 1 % MnO_2				–	7.1			0.700	7.4	0.20	[31]
98.75 % ZnO, 0.25 % V_2O_5, 1 % Co_3O_4				–	8.4	ZnO	α-$Zn_3(VO_4)_2$	0.550	6.4	0.25	[31]
97.75 % ZnO, 0.25 % V_2O_5, 2 % Sb_2O_3			1200 °C / 4 h	–	2.6	ZnO	$Zn_7Sb_2O_{12}$	1.000	8.0	0.40	[31]
95.75 % ZnO, 0.25 % V_2O_5, 2 % Sb_2O_3, 1 % MnO_2, 1 % Co_3O_4				–	3.3			2.000	18.9	0.05	[31]

[1] RS = rotational speed, MD = milling duration, BPR = ball-to-powder ratio, GM = grinding media;
[2] P_P = pressing pressure, D x h of GC = diameter x height of green compacts, HR = heating rate, CR = cooling rate;
~ values approximated from the graphs published in the referenced publication; - values not determined in the referenced publication

Table S2. Processing parameters employing two stage sintering (TSS) in air and main physical, structural and electrical properties of MOVs from the selected ZnO-V_2O_5-based systems.

Composition of MOV systems (mol. %)	Ball milling parameters [1]	PM processing parameters [2]			Relative density (RD) of MOVs (% of TD)	Mean ZnO grain size (μm)	Crystalline phases		Breakdown field, E_B (kV/cm)	Nonlinear exponent (α)	Leakage current density, J_L (mA/cm^2)	Reference
		Pressing pressure; D × h of green compacts; Heating rate / cooling rate	Sintering temp. T_1 / Dwell time t_1	Sintering temp. T_2 / Dwell time t_2			Primary phase	Secondary phases				
97.4 % ZnO, 0.5 % V_2O_5, 2 % MnO_2, 0.1 % Nb_2O_5	RS = 300 rpm; MD = 35 h (dry); BPR = 16:1; GM = agate vial and agate balls	P_P = 500 MPa; Ø10 mm × 1.5 mm; HR from RT to T_1 = 15 °C/min CR from T_1 to T_2 = 30°C/min	1050 °C / 1/6 h	750 °C / 2.5 h	97.7 ± 0.2	3.5 ± 0.2	ZnO	$Zn_3(VO_4)_2$, $Zn_4V_2O_9$, ZnV_2O_4, V_2O_5, Zn_2MnO_4	3.8 ± 0.3	12 ± 2	0.37 ± 0.03	[14]
				750 °C / 10 h	97.8 ± 0.2	3.8 ± 0.2			4.4 ± 0.4	12 ± 1	0.37 ± 0.03	
				750 °C / 20 h	98.2 ± 0.2	4.2 ± 0.2			5.9 ± 0.4	42 ± 3	0.24 ± 0.03	
				750 °C / 40 h	98.5 ± 0.2	4.3 ± 0.2			6.2 ± 0.4	53 ± 3	0.20 ± 0.03	
			1050 °C / 1/6 h	800 °C / 2.5 h	98.1 ± 0.2	3.6 ± 0.2			3.6 ± 0.2	12 ± 1	0.38 ± 0.01	[14]
				800 °C / 10 h	98.4 ± 0.2	4.1 ± 0.2			3.8 ± 0.3	13 ± 1	0.34 ± 0.01	
				800 °C / 20 h	98.5 ± 0.2	4.5 ± 0.2			4.3 ± 0.4	15 ± 1	0.31 ± 0.01	
				800 °C / 40 h	98.7 ± 0.2	4.8 ± 0.2			4.4 ± 0.4	31 ± 2	0.26 ± 0.03	
96.9 % ZnO, 0.5 % V_2O_5, 2 % MnO_2, 0.1 % Nb_2O_5, 0.5 % Er_2O_3	RS = 300 rpm; MD = 35 h (dry); BPR = 16:1; GM = agate vial and agate balls	P_P = 500 MPa; Ø10 mm × 1.5 mm; HR from RT to T_1 = 15 °C/min CR from T_1 to T_2 = 30°C/min	1050 °C / 1/6 h	750 °C / 2.5 h	96.2 ± 0.2	0.9 ± 0.1	ZnO	$Zn_3(VO_4)_2$, $Zn_4V_2O_9$, ZnV_2O_4, V_2O_5, Zn_2MnO_4, $ErVO_4$	9.2 ± 0.8	106 ± 8	0.24 ± 0.03	[14]
				750 °C / 10 h	96.3 ± 0.2	1.3 ± 0.1			10.3 ± 0.9	134 ± 15	0.20 ± 0.03	
				750 °C / 20 h	97.0 ± 0.2	1.6 ± 0.1			11.1 ± 0.9	139 ± 16	0.17 ± 0.02	
				750 °C / 40 h	97.5 ± 0.2	1.7 ± 0.1			15.2 ± 1.1	154 ± 18	0.13 ± 0.02	
			1050 °C / 1/6 h	800 °C / 2.5 h	97.0 ± 0.2	1.1 ± 0.1			9.1 ± 0.7	75 ± 3	0.26 ± 0.03	[14]
				800 °C / 10 h	97.6 ± 0.2	1.4 ± 0.1			10.2 ± 0.9	80 ± 4	0.25 ± 0.03	
				800 °C / 20 h	97.6 ± 0.2	1.8 ± 0.2			11.0 ± 0.9	107 ± 8	0.17 ± 0.02	
				800 °C / 40 h	97.7 ± 0.2	2.1 ± 0.2			11.1 ± 0.9	146 ± 17	0.15 ± 0.02	

[1] RS = rotational speed, MD = milling duration, BPR = ball-to-powder ratio, GM = grinding media
[2] P_P = pressing pressure, D × h of GC = diameter × height of green compacts, HR = heating rate, CR = cooling rate

References

1. El-Rabaie, S.; Khafagy, A.H.; Dawoud, M.T.; Attia, M.T. Mechanical, microstructure and electrical properties of ternary ZnO–V_2O_5–Mn_3O_4 varistor with sintering temperature. *Bull. Mater. Sci.* **2015**, *38*, 773–781. https://doi.org/10.1007/s12034-015-0903-2.
2. Nahm, C.-W. Effect of Bi_2O_3 doping on microstructure and electrical properties of ZnO–V_2O_5–Mn_3O_4 semiconducting ceramics. *J. Mater. Sci. Mater. Electron.* **2017**, *28*, 903–908. https://doi.org/10.1007/s10854-016-5605-z.
3. Nahm, C.-W. Effect of Mn doping on electrical properties and accelerated ageing behaviours of ternary ZVM varistors. *Bull. Mater. Sci.* **2011**, *34*, 1385–1391. https://doi.org/10.1007/s12034-011-0333-8.
4. Park, J.-H.; Nahm, C.-W. Sintering effect on electrical properties and aging behavior of quaternary ZnO–V_2O_5–Mn_3O_4–Nb_2O_5 ceramics. *J. Mater. Sci. Mater. Electron.* **2015**, *26*, 168–175. https://doi.org/10.1007/s10854-014-2379-z.
5. Nahm, C.-W. Sintering effect on electrical properties and pulse aging behavior of (V_2O_5-Mn_3O_4-Er_2O_3)-doped zinc oxide varistor ceramics. *J. Rare Earths* **2014**, *32*, 29–36. https://doi.org/10.1016/S1002-0721(14)60030-2.
6. Nahm, C.-W. Effect of sintering temperature on nonlinearity and surge degradation characteristics of Mn_3O_4/Nb_2O_5/Er_2O_3-doped ZnO–V_2O_5-based varistors. *J. Korean Ceram. Soc.* **2020**, *57*, 65–72. https://doi.org/10.1007/s43207-019-00009-9.
7. Nahm, C.-W. Effect of Yb_2O_3 addition on varistor properties and aging characteristics of ZnO–V_2O_5–Mn_3O_4 system. *J. Mater. Sci. Mater. Electron* **2018**, *29*, 2958–2965. https://doi.org/10.1007/s10854-017-8226-2.
8. Nahm, C.-W. Microstructure, electrical and dielectric properties, and impulse clamping characteristics of ZnO–V_2O_5–Mn_3O_4 semiconducting ceramics modified with Er_2O_3. *J. Mater. Sci. Mater. Electron.* **2013**, *24*, 4129–4136. https://doi.org/10.1007/s10854-013-1371-3.
9. Nahm, C.-W. Effect of sintering temperature on varistor properties and aging characteristics of ZnO–V_2O_5–MnO_2 ceramics. *Ceram. Int.* **2009**, *35*, 2679–2685. https://doi.org/10.1016/j.ceramint.2009.03.011.
10. Roy, T.K. High nonlinearity in 0.1 mol.% In_2O_3 added ZnO–V_2O_5 based varistors prepared at different sintering temperatures. *Ceram. Int.* **2021**, *47*, 35152–35159. https://doi.org/10.1016/j.ceramint.2021.09.058.
11. Roy, T.K.; Bhattacharyya, T.K.; Thakur, S.K. Role of sintering temperature on microstructure and nonlinear electrical properties of 0.1 mol.% Nb_2O_5 added ZnO–V_2O_5 varistor ceramics. *J. Mater. Sci. Mater. Electron.* **2019**, *30*, 5640–5651. https://doi.org/10.1007/s10854-019-00857-5.
12. Roy, S.; Das, D.; Roy, T.K. Nonlinear electrical properties of ZnO-V_2O_5 based rare earth (Er_2O_3) added varistors. *J. Electron. Mater.* **2019**, *48*, 5650–5661. https://doi.org/10.1007/s11664-019-07394-1.
13. Roy, S.; Das, D.; Roy, T.K. Influence of sintering temperature on microstructure and electrical properties of Er_2O_3 added ZnO-V_2O_5-MnO_2-Nb_2O_5 varistor ceramics. *J. Alloys Compd.* **2018**, *749*, 687–696. https://doi.org/10.1016/j.jallcom.2018.03.302.
14. Roy, S.; Das, D.; Roy, T.K. Two stage sintering behaviour of Er_2O_3 doped high performance ZnO varistors. *J. Eur. Ceram. Soc.* **2021**, *41*, 5184–5192. https://doi.org/10.1016/j.jeurceramsoc.2021.04.009.
15. Nahm, C.-W. Effect of sintering process on electrical properties and ageing behavior of ZnO–V_2O_5–MnO_2–Nb_2O_5 varistor ceramics. *J. Mater. Sci. Mater. Electron.* **2012**, *23*, 457–463. https://doi.org/10.1007/s10854-011-0512-9.
16. Nahm, C.W. Effect of small changes in sintering temperature on varistor properties and degradation behavior of V–Mn–Nb–Gd co-doped zinc oxide ceramics. *Trans. Nonferrous Met. Soc. China* **2015**, *25*, 1176–1184. https://doi.org/10.1016/S1003-6326(15)63713-X.
17. Nahm, C.-W. Electrical and dielectric characteristics of erbium-added ZnO–V_2O_5-based varistor ceramics. *Ceram. Int.* **2012**, *38*, 6651–6658. https://doi.org/10.1016/j.ceramint.2012.05.052.
18. Wu, J.; Li, T.; Qi, T.; Qin, Q.; Li, G.; Zhu, B.; Wu, R.; Xie, C. Influence of dopants on electrical properties of ZnO-V_2O_5 varistors deduced from AC impedance and variable-temperature dielectric spectroscopy. *J. Electron. Mater.* **2012**, *41*, 1970–1977. https://doi.org/10.1007/s11664-012-1935-7.
19. Pandey, S.; Kumar, D.; Parkash, O. Investigation of the electrical properties of liquid-phase sintered ZnO–V_2O_5 based varistor ceramics using impedance and dielectric spectroscopy. *J. Mater. Sci. Mater. Electron.* **2016**, *27*, 3748–3758. https://doi.org/10.1007/s10854-015-4218-2.
20. Nahm, C.-W. Influence of Bi_2O_3 doping on microstructure and electrical properties of ZnO-V_2O_5-MnO_2-Nb_2O_5 varistor ceramics. *J. Am. Ceram. Soc.* **2012**, *95*, 2093–2095. https://doi.org/10.1111/j.1551-2916.2012.05230.x.
21. Nahm, C.-W. Effects of low-temperature sintering on varistor properties and stability of VMCDNB-doped zinc oxide ceramics. *J. Korean Ceram. Soc.* **2019**, *56*, 84–90. https://doi.org/10.4191/kcers.2019.56.1.11.
22. Nahm, C.-W. Effect of low-temperature sintering on electrical properties and aging behavior of ZVMNBCD varistor ceramics. *Korean. J. Mater. Res.* **2020**, *30*, 502–508. https://doi.org/10.3740/MRSK.2020.30.10.502.
23. Nahm, C.-W. Effect of Dy_2O_3 doping on microstructure, electrical and dielectric properties of ZnO–V_2O_5-based varistor ceramics. *J. Mater. Sci. Mater. Electron.* **2015**, *26*, 10217–10224. https://doi.org/10.1007/s10854-015-3874-6.
24. Nahm, C.-W. Microstructure and varistor properties of ZVMND ceramics with sintering temperature. *Trans. Electr. Electron. Mater.* **2015**, *16*, 221–225. https://doi.org/10.4313/TEEM.2015.16.4.221.
25. Roy, S.; Roy, T.K.; Das, D. Microstructure and current-voltage characteristics of erbium oxide doped multicomponent zinc oxide varistors. *IOP Conf. Ser. Mater. Sci. Eng.* **2018**, *338*, 012046. https://doi.org/10.1088/1757-899X/338/1/012046.
26. Zhao, M.; Li, X.; Li, T.; Shi, Y.; Li, B. Effect of Y_2O_3, Nd_2O_3 or Sm_2O_3 on the microstructure and electrical properties of ZnVMnNbO varistor ceramics. *J. Mater. Sci. Mater. Electron.* **2019**, *30*, 450–456. https://doi.org/10.1007/s10854-018-0309-1.

27. Zhao, M.; Wang, Y.-H.; Li, X.; Song, H.-H.; Sun, T.-T. Sintering mechanism and properties of ZnVMnNbO varistor ceramic and their evolutions by Ce–La doping. *Ceram. Int.* **2020**, *46*, 20923–20931. https://doi.org/10.1016/j.ceramint.2020.05.148.
28. Pandey, S.; Kumar, D.; Parkash, O. Electrical impedance spectroscopy and structural characterization of liquid-phase sintered ZnO–V_2O_5–Nb_2O_5 varistor ceramics doped with MnO. *Ceram. Int.* **2016**, *42*, 9686–9696. https://doi.org/10.1016/j.ceramint.2016.03.057.
29. Roy, S.; Kundu Roy, T.; Das, D. Sintering of nanocrystalline multicomponent zinc oxide varistor powders prepared by ball milling. *Mater. Today: Proc.* **2018**, *5*, 9899–9909. https://doi.org/10.1016/j.matpr.2017.10.185.
30. Xu, M.; Cai, C.; Shi, Y.; Xie, M.; Wu, Y.; Liu, Y.; Peng, J.; Bao, J.; An, S. The grain growth control of ZnO-V_2O_5 based varistors by $PrMnO_3$ addition. *Micromachines* **2022**, *13*, 214. https://doi.org/10.3390/mi13020214.
31. Hng, H.-H.; Knowles, K.M. Microstructure and current-voltage characteristics of multicomponent vanadium-doped zinc oxide varistors. *J. Am. Ceram. Soc.* **2004**, *83*, 2455–2462. https://doi.org/10.1111/j.1151-2916.2000.tb01576.x.
